# Asymmetric BODIPY Dyes Enabling Triplet–Triplet Annihilation Upconversion

**DOI:** 10.1021/acsaom.4c00285

**Published:** 2024-07-13

**Authors:** Daniel Álvarez-Gutiérrez, Diego Sampedro, M. Consuelo Jiménez, Raúl Pérez-Ruiz

**Affiliations:** † Departamento de Química, Universitat Politècnica de València, Camino de Vera S/N, 46022 Valencia, Spain; ‡ Departamento de Química, Instituto de Investigación en Química de la Universidad de La Rioja (IQUR), Universidad de La Rioja, Madre de Dios 53, 26006 Logroño, Spain

**Keywords:** photon upconversion, triplet−triplet annihilation, BODIPYs, asymmetric, visible light

## Abstract

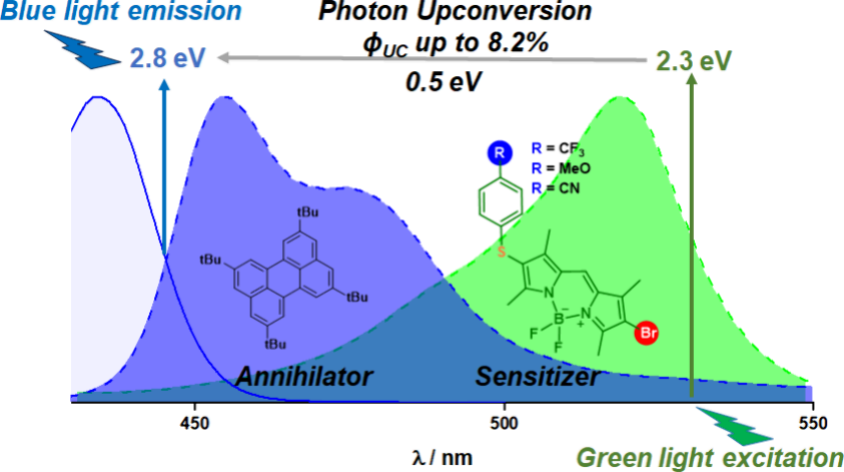

The construction of triplet–triplet annihilation upconversion (TTA-UC) systems with upconversion (UC) emission efficiency at low power densities is still under continuing exploration. From an environmental point of view, the utilization of purely organic pairs is more beneficial than the involvement of transition-metal complexes. In this context, 4,4-difluoro-4-bora-3a,4a-diaza-*s*-indacene (BODIPY) dyes, which can be found in a wide range of applications, have been previously used as suitable sensitizers in TTA-UC systems. The versatility of these scaffolds makes them magnificent objectives for designing and synthesizing potential entities with different target abilities. Herein, we prepared several asymmetric BODIPY dyes with excellent optical properties to be applied to a bimolecular TTA-UC system. In the presence of 2,5,8,11-tetra-*tert*-butylperylene (**TBPe**) as a suitable annihilator, a green-to-blue light conversion was clearly observed by means of detailed spectroscopic investigations. The results revealed a high UC emission efficiency (η_UC_) of ∼8%, together with a low threshold intensity (*I*_th_) of ∼40–50 mW/cm^2^. All data indicated that these asymmetric BODIPY dyes were ideal sensitizers for TTA-UC, providing a particular design for further investigations.

## 1 Introduction

The so-called photon upconversion (UC) technology permits the conversion of long wavelengths (photons with low energy) to shorter wavelengths (photons with higher energy).^1^ Thanks to the optical characteristics of UC, it has found application in different fields such as materials science,^2^ biology,^3^ and chemistry,^4^ among others. Several mechanisms can operate through UC: second harmonic generation,^5^ multistep excitation of lanthanides,^6^ two-photon absorption,^7^ and triplet–triplet annihilation (TTA).^8^ In this study, we have focused our attention on TTA-based upconversion (TTA-UC) because this phenomenon is particularly promising due to its ability to have a high UC efficiency (η_UC_), a low excitation intensity (such as solar irradiance), and tunable absorption and emission wavelengths.

**Figure 1:**
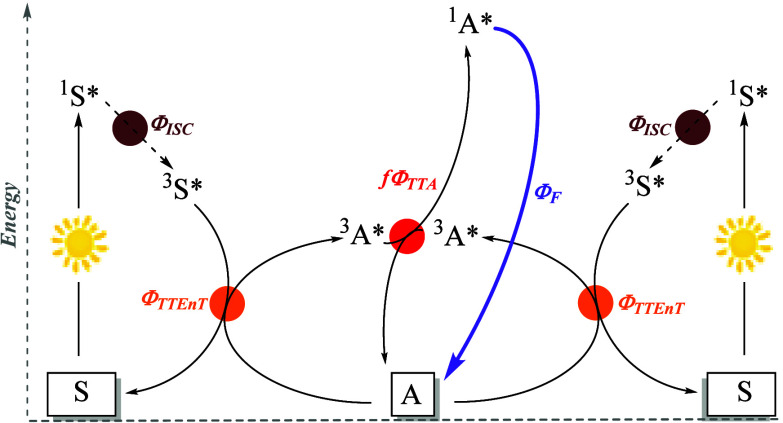
Scheme of the TTA-UC mechanism together with the parameters related to the UC efficiency (η_UC_). S = sensitizer; A = annihilator; ISC = intersystem crossing; TTEnT = triplet–triplet energy transfer; TTA = triplet–triplet annihilation; F = fluorescence; f = spin statistical factor.


Usually, a TTA-UC system comprises two chromophores: a sensitizer (also called a donor) and an annihilator (also known as an acceptor or emitter). Thus, the appropriate selection of a sensitizer/annihilator couple is crucial to optimizing the η_UC_ value. After selective irradiation of the sensitizer at high wavelength, its excited singlet state ^1^[S]* intersystem crosses the corresponding triplet state ^3^[S]*. Then, rapid and efficient energy transfer to A generates the corresponding triplet state ^3^[A]*. Two such intermediates can undergo TTA, resulting in a delayed fluorescence from ^1^[A]* that occurs at a higher frequency than that of the exciting light, i.e., upconverted fluorescence (Figure 1). One of the most important parameters that characterizes the TTA-UC process is η_UC_, which is defined as the product of the efficiency of each process, as shown in eq 1 (Figure 1).^9^ Because of the two-to-one photon nature of TTA-UC, the maximum theoretical value of η_UC_ is 0.5 or 50%. This value is sometimes multiplied by two or normalized to 1.0 or 100%. It is worth mentioning that a sensitizer must possess intense absorption, a high triplet state efficiency, and a long triplet lifetime. Then, the triplet–triplet energy-transfer efficiency (ϕ_TTET_) could be considerably improved with a high triplet state efficiency and long triplet lifetime.^10^ In addition, the 2-fold T_1_ energy levels of the annihilator might be greater than its S_1_ energy level (2T_1_ > S_1_), enabling the TTA process of triplet annihilators. The spin statistical factor (*f*) is the singlet production probability by TTA.^11^ Hence, a favorable impact will be found when a 2-fold T_1_ energy level of the annihilator would be lower than its T_2_ energy level (2T_1_ < T_2_).^12^ Finally, η_UC_ will be enhanced by the high fluorescence quantum yield of the annihilator (ϕ_f_).^13^

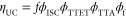
1

Regarding annihilators, they are organic chromophores, usually polycyclic aromatic hydrocarbons.^14^ The S_1_ and T_1_ energy levels of the annihilators can be adjusted by performing chemical modifications on the molecular structures; subsequent matching with the appropriate sensitizer will allow enhancement of η_UC_.^15^ The most important requisites for annihilators, among others, are high ϕ_f_ in the UV or UV–near-visible region, long triplet lifetimes, and a large energy band gap between the singlet and triplet excited states.

In the case of sensitizers, metal complexes (Pt, Ru, Ir, and Re complexes),^8,16^ heavy-atom free sensitizers,^17^ inorganic sensitizers such as metal clusters,^18^ nanocrystal quantum dots,^19^ perovskites,^20^ lanthanide complexes,^21^ complexes with more earth-abundant metals,^22^ or even molecules showing thermally activated delayed fluorescence^23^ have been successfully employed in recent years. However, these systems exhibit some drawbacks, which include complex and expensive synthetic protocols, structural instability, low photostability, or a restricted functional range of solvent conditions, including the toxicity of transition metals. Therefore, it appears interesting to use more accessible organic sensitizers that accomplish the following needs: high molar absorption coefficients with insensitivity to solvent environments and high photostability. Although a number of organic sensitizers have been previously described, which all have their strengths and challenges,^24^ 4,4-difluoro-4-bora-3a,4a-diaza-*s*-indacene (BODIPY) derivatives fulfill the above-mentioned requirements and can be employed either as an annihilator or as a sensitizer.^25^

Herein, we focus on the capacity of BODIPY dyes to act as sensitizers. In particular, halogenated BODIPY dyes have the undoubted advantage of large molar absorption coefficients, maximizing the probability of generating the singlet excited state and high photostability, reducing side photoreactions.^26^ The presence of halogen atoms as substituents (heavy-atom effect)^27^ increases the intersystem crossing quantum yield (ϕ_ISC_) due to the increased spin–orbit coupling and decreases the energy band gap between the singlet and triplet excited states to boost triplet production.^28^ Previous studies of BODIPY-based sensitizers in bimolecular TTA-UC systems can be found in the literature.^29^ We have very recently investigated an asymmetric BODIPY that exhibited excellent photophysical properties, which makes it an excellent candidate to be used as a sensitizer in a bimolecular TTA-UC system.^30^ Its combination with 2,5,8,11-tetra-*tert*-butylperylene (**TBPe**) allowed the conversion of green to blue light with η_UC_ of 2.7%, which was found to be lower than those from quantum-yield-optimized systems. To enhance the η_UC_ value, the design and development of new sensitizers are highly desired.

**Chart 1:**
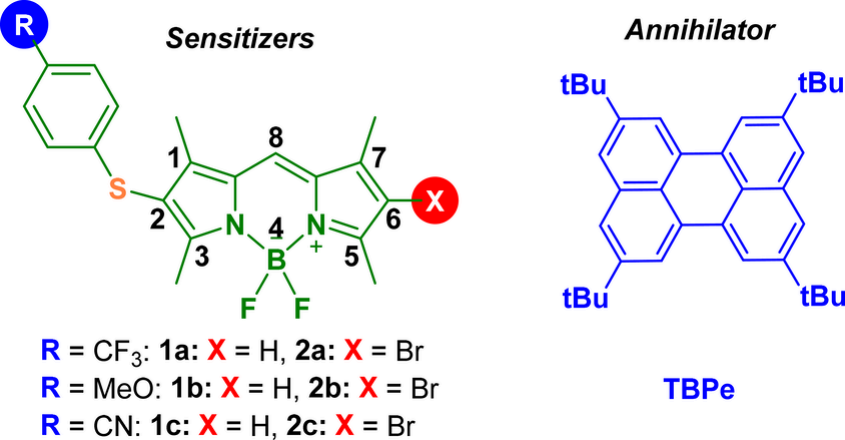
Molecular Structures of the New Asymmetric BODIPY Dyes and **TBPe**

Because of the versatile derivatization of the BODIPY chromophore, we describe herein our endeavors toward the synthesis of new asymmetric BODIPY dyes (**2a**–**2c**) with special optical properties to be used as appropriate sensitizers in TTA-UC systems (Chart 1). Note that the introduction of both a para-substituted thiophenol and a bromine at the 2 and 6 positions, respectively, of the indacene core could not only alter the triplet energy value but also shift the UV–vis absorption band to the green region. This specific molecular design permits a low energetic light input (λ_exc_ = 530 nm) and avoids the presence of iodine, which frequently results in photolabile dyes. The role of these new asymmetric BODIPY dyes as efficient sensitizers, with **TBPe** (Chart 1) as the triplet energy annihilator, is fully demonstrated through spectroscopic methods.

## 2 Results and Discussion

### 2.1 Synthesis of Asymmetric BODIPY Dyes

Encouraged by the ongoing progress in obtaining new BODIPY dyes, the principal aim of this study was to synthesize new BODIPY-like sensitizers to check whether these entities could play a key role in a bimolecular system for TTA-UC purposes. Remarkably, these new scaffolds presented an asymmetric structure: a 4-substituted phenyl sulfide group and a bromine atom attached at positions 2 and 6, respectively, of the indacene core (Chart 1). Therefore, a particular electron density distribution was conferred to the whole chromophore to display strong absorption of visible light, an efficient intersystem crossing (ISC), and a long-lived triplet state.

**Scheme 1:**
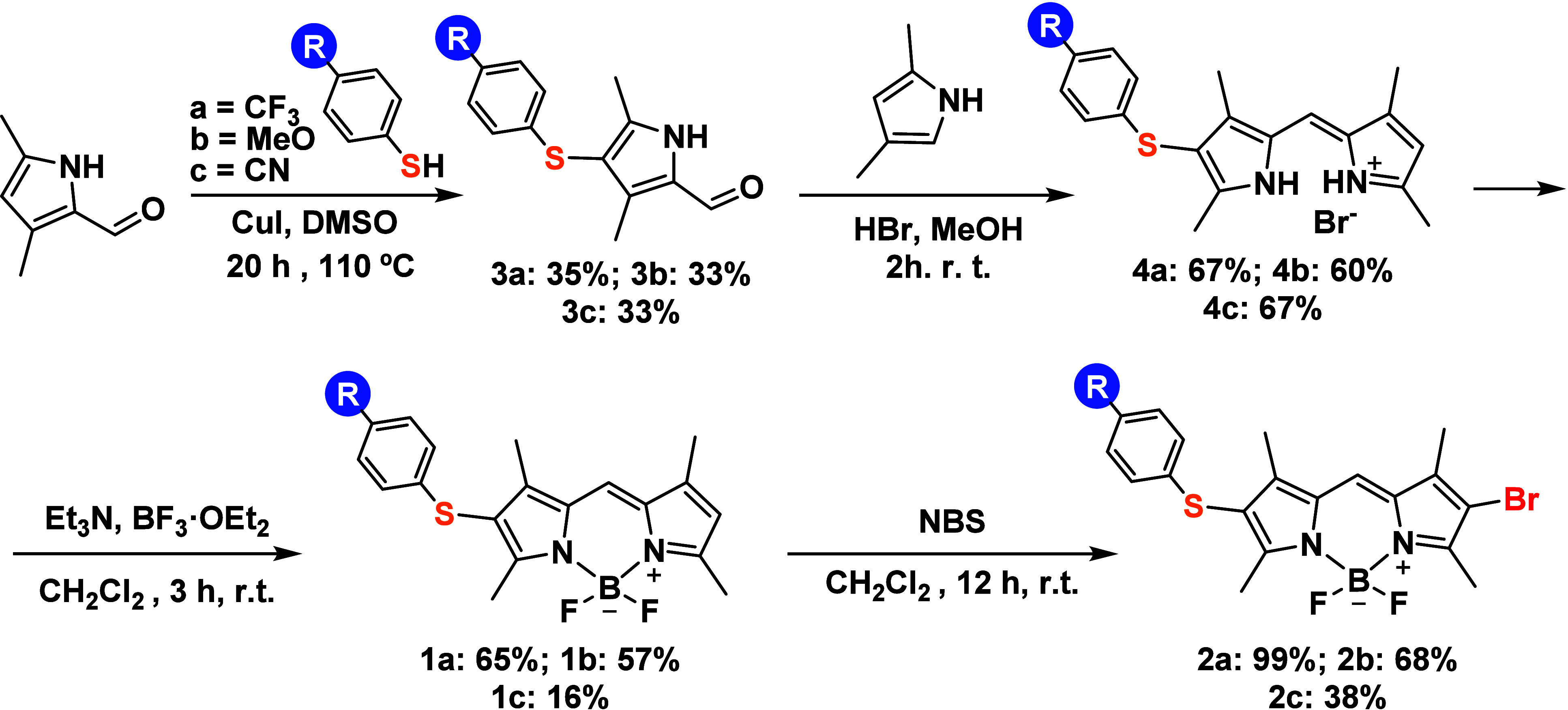
Synthetic Route for the Formation of BODIPY Dyes For more details on the synthetic procedures, see the Experimental Section.

The synthetic procedure (Scheme 1) started with the addition of the corresponding 4-substituted thiophenol to the starting material 3,5-dimethylpyrrole-2-carboxaldehyde, catalyzed by copper iodide.^31^ Isolated yields of the corresponding products **3a**–**3c** were 35%, 33% and 33%, respectively. The next step was crucial to building the asymmetrical structure. Thus, condensation of **3a**–**3c** with 2,4-dimethylpyrrole gave rise to the formation of intermediate dipyrromethanes **4a**–**4c** with 67%, 60%, and 67% yields, respectively. Then, compounds **4a**–**4c** were treated with triethylamine as the base, followed by the addition of boron trifluoride diethyl etherate,^32^ obtaining the corresponding BODIPY dyes **1a**–**1c** in 65%, 57%, and 16% isolated yields. In the last step, bromine was incorporated into **1a**–**1c** by treatment with *N*-bromosuccinimide (NBS), leading to target compounds **2a**–**2c** with 99%, 68%, and 38% isolated yields, respectively. Their structures were fully characterized by spectroscopic techniques [see the Experimental Section and Supporting Information (SI) for details]. The characteristic hydrogen present at position 8 of the indacene core was unambiguously evidenced by NMR, which supported the location of bromine at position 6. As a matter of fact, the design of **2a**–**2c** could influence the ISC quantum yield. The presence of the 4-substituted phenyl sulfide group would cause a weakly emissive twisted intramolecular charge-transfer excited state (TICT)^33^ between the sulfur’s lone electron pair and an excited electron-deficient BODIPY moiety. Besides, the presence of heavy atoms such as bromine would directly affect the triplet-state lifetime of the molecule, favoring the ISC process.^34^ Therefore, BODIPY dyes **2a**–**2c** are excellent candidates to incorporate in the design of new TTA-UC systems.

### 2.2 Absorption and Fluorescence

Once the target compounds **2a**–**2c** were isolated, the next step was to investigate the optical properties, which include recording the absorption/emission bands and determining the molar absorption coefficients, Stokes shifts, fluorescence quantum yields, emission rate constants, singlet energies, and lifetimes in different solvents (Table 1). Thus, compounds **2a**–**2c** presented absorption bands with wavelength maxima (λ_max,abs_) in the green region (519–531 nm) in all solvents, whereas dependence on the solvent polarity was found to be weak (Table 1 and Figure S2). Regarding molar absorption coefficients (ε), Stokes shifts (Δν̅), and fluorescence lifetimes, they showed outputs on the same order of magnitude as those previously found for similar BODIPYs.^33^ Interestingly, lower absolute fluorescence quantum yields (ϕ_F_) were, in general, obtained in more polar solvents, suggesting that the ISC efficiency from the lowest singlet excited state to the triplet would be enhanced by the formation of a TICT state. The incorporation of bromine at position 6 of the indacene core affected importantly ϕ_F_ values in comparison with the results obtained in the absence of the heavy atom. In addition, the phosphorescence spectra of **2a**–**2c** were recorded in an ethanol matrix at 77 K (Figure S4), where λ_max_ at ∼770 nm was found in all cases, which was in agreement with the values previously reported for other halogen-substituted BODIPYs.^35^ Furthermore, the lowest triplet energies (*E*_T_) were estimated as 1.71, 1.71, and 1.70 eV for **2a**–**2c**, respectively, being higher than that of **TBPe** (*E*_T_ = 1.53 eV).^36^ Thus, energy transfer from the T_1_ of **2a**–**2c** to the T_1_ of **TBPe** is thermodynamically allowed.

**Table 1:** Optical Properties of the BODIPY Dyes **1a**–**1c** and **2a**–**2c** in Different Solvents

	**1a**/**2a**							
solvent	λ_max,abs_^a^	λ_max,em_^a^	ε^b^	Δν̅^c^	*E*_s_^d^	ϕ_F_^e^	*τ*_f_^f^	*k*_f_^g^
*n*-hexane	511/528	557/572	75453/73668	1616/1457	2.38/2.30	95/50	5.02/3.42	1.9 × 10^8^/1.5 × 10^8^
DCM	510/527	578/590	78743/66190	2307/2026	2.36/2.29	60/30	4.07/2.76	1.4 × 10^8^/1.1 × 10^8^
MeCN	503/519	584/597	71962/66922	2757/2517	2.38/2.31	30/17	3.49/2.10	8.6 × 10^7^/8.3 × 10^7^
MeOH	503/520	576/588	72390/53539	2520/2224	2.38/2.31	38/19	3.47/1.95	1.1 × 10^8^/9.6 × 10^7^
DMSO	505/521	587/601	76254/59774	2766/2555	2.37/2.30	38/14	2.93/1.46	1.3 × 10^8^/9.7 × 10^7^

	**1b**/**2b**							
solvent	λ_max,abs_^a^	λ_max,em_^a^	ε^b^	Δν̅^c^	*E*_s_^d^	Φ_f_^e^	*τ*_f_^f^	*k*_f_^g^
*n*-hexane	516/531	592/610	68096/69776	2488/2439	2.27/2.20	44/23	4.37/2.96	1.0 × 10^8^/7.5 × 10^7^
DCM	514/530	641/669	68660/65660	3855/3920	2.29/2.19	5/1	0.59/0.39	9.4 × 10^7^/4.1 × 10^7^
MeCN	507/522	n.d./538	66331/62109	0/570	2.45/2.34	0.42/0.16	–/–	–/–
MeOH	508/523	n.d./578	69449/60870	0/1819	2.44/2.31	0.78/0.13	–/–	–/–
DMSO	509/524	n.d./543	68807/59212	115/668	2.43/2.34	0.06/0.10	–/–	–/–

	**1c**/**2c**							
solvent	λ_max,abs_^a^	λ_max,em_^a^	ε^b^	Δν̅^c^	*E*_s_^d^	Φ_f_^e^	*τ*_f_^f^	*k*_f_^g^
*n*-hexane	510/528	551/568	50880/90894	1459/1334	2.38/2.30	96/58	4.98/3.15	1.9 × 10^8^/1.8 × 10^8^
DCM	509/526	568/583	57018/102559	2041/1859	2.37/2.30	71/37	4.05/2.73	1.8 × 10^8^/1.4 × 10^8^
MeCN	502/519	575/589	51242/91841	2529/2290	2.38/2.31	42/20	2.76/1.59	1.5 × 10^8^/1.3 × 10^8^
MeOH	503/519	569/582	47151/96669	2306/2086	2.41/2.32	44/26	3.46/1.94	1.3 × 10^8^/1.4 × 10^8^
DMSO	504/521	582/597	49813/67753	2659/2443	2.38/2.29	44/18	3.17/1.86	1.4 × 10^8^/9.7 × 10^7^

a Maximum absorption/emission wavelengths (in nm).

b Molar absorption coefficients (in M^–1^ cm^–1^).

c Stokes shifts (in cm^–1^).

d Singlet energies (in eV).

e Absolute fluorescence quantum yields (in %).

f Singlet lifetimes (in ns).

g Fluorescence rate constant (in s^–1^). DCM = dichloromethane; MeCN = acetonitrile; MeOH = methanol; DMSO = dimethyl sulfoxide.

Compounds **2a**–**2c** were found to be stable in an acetonitrile (MeCN) solution (*c* = 10^–6^ M) in the dark at room temperature. In order to study the inherent photostability of **2a**–**2c**, solutions of the corresponding BODIPY dyes in deaerated MeCN (*c* = 10^–6^ M) were submitted to direct irradiation in a multilamp photoreactor, using 5 W lamps (10×) emitting at λ_max_ = 522 nm. The reactions were followed by UV–vis absorption spectroscopy, wherein a decrease of the absorption band was practically negligible; meanwhile, new absorption bands were not observed, revealing an outstanding photostability of **2a**–**2c** after 3 h (Figure S13).

### 2.3 ISC Quantum Yield (ϕ_ISC_)

**Figure 2:**
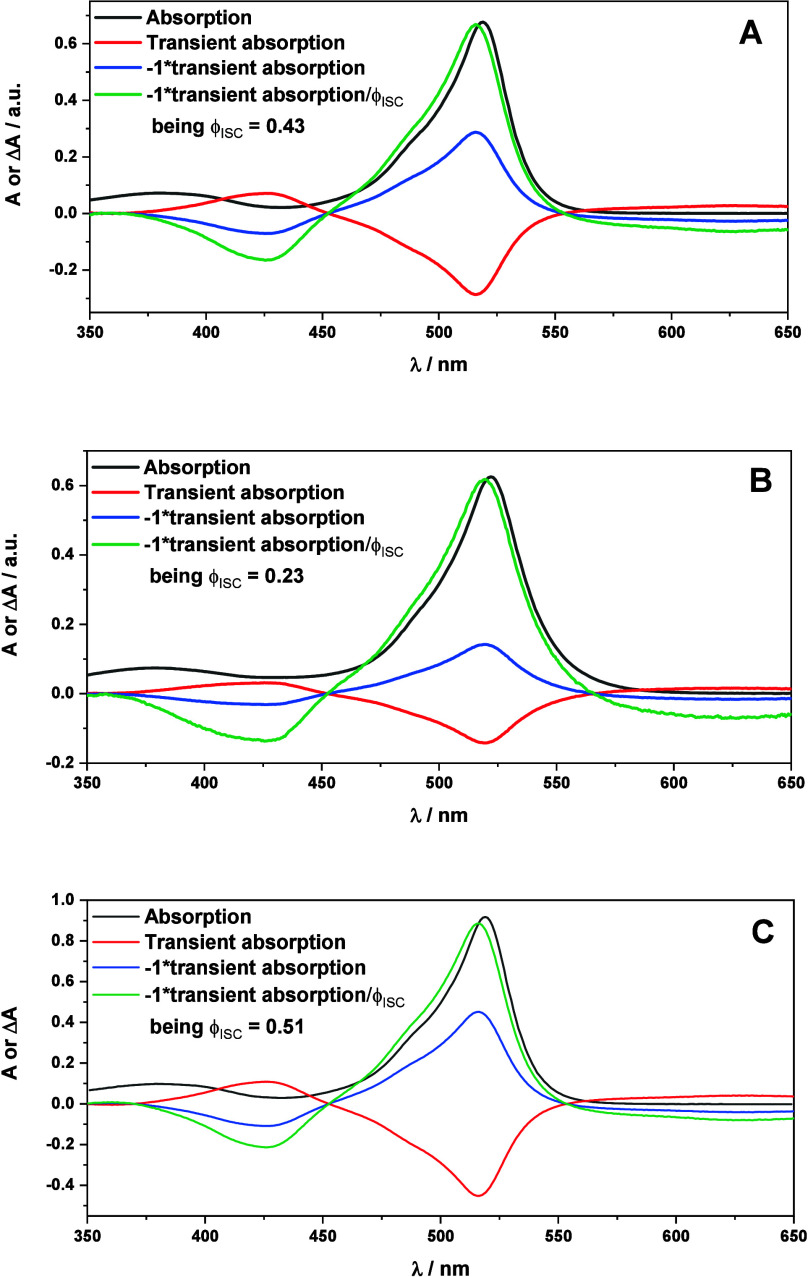
Graphical representation for the calculation of ϕ_ISC_ of **2a** (A), **2b** (B), and **2c** (C). For further details, see the SI.

Estimation of the ISC quantum yield (ϕ_ISC_) of the sensitizer is one of the key parameters to determine the TTA-UC efficiency, η_UC_. Frequently, an indirect measurement through the quantum yield of singlet oxygen generation has been employed for determining ϕ_ISC_ in BODIPY derivatives.^37^ To gain more reliable results in a direct fashion, we here determine the ϕ_ISC_ value of BODIPY dyes **2a**–**2c** following a previously reported procedure that makes use of nanosecond transient absorption measurements.^38^ Thus, spectroscopic data leading to ϕ_ISC_ determination are presented in Figure 2: absorption spectrum [*A*(λ), black line], transient spectrum [*A*^transient^(λ), red line], transient spectrum multiplied by −1 [(−1)*A*^transient^(λ), blue line], and transient spectrum multiplied by −1 and divided by ϕ_ISC_ [(−*A*^transient^(λ)/ϕ_ISC_, green line] for a given ϕ_ISC_ value (further details are given in the SI). Thus, the ϕ_ISC_ values were found to be 0.43, 0.23, and 0.51 for **2a**–**2c**, respectively, in agreement with the reported data for other BODIPY dyes.^33,38^*A priori*, in the case of **2b**, the methoxy group could somehow influence the efficiency of triplet production; however, the presence of bromine attached directly to the chromophoric core substantially enhanced the ISC in comparison with unsubstituted BODIPY, which exhibited negligible ISC.^39^ Indeed, dyes **1a**–**1c** presented, in general, higher fluorescence quantum yields and longer singlet lifetimes compared to dyes **2a**–**2c** (Table 1), which allows one to attribute the increment of ϕ_ISC_ mainly to the heavy-atom effect of brmine rather than to the substituted phenyl sulfide moiety. The high molar absorption coefficient in the green region for **2a**–**2c**, together with their high photostability, clearly pointed out that these entities are suitable for employment as sensitizers for triplet-energy-transfer-driven chemistry.

### 2.4 Triplet–Triplet Energy Transfer (TTEnT)

As mentioned above, the energies of the triplet excited states of **2a**–**2c** were estimated as 1.71, 1.71, and 1.70 eV, respectively. Due to the rather low triplet-state energy of **TBPe** (1.53 eV),^36^ the high singlet-state energy of 2.81 eV, and the absence of unwanted excimer formation^40^ (due to the presence of *tert*-butyl groups), **TBPe** was chosen as an adequate annihilator. Experimental evidence for the TTEnT process was demonstrated by means of laser flash photolysis (LFP) experiments in the nanosecond-to-microsecond domain.

**Figure 3:**
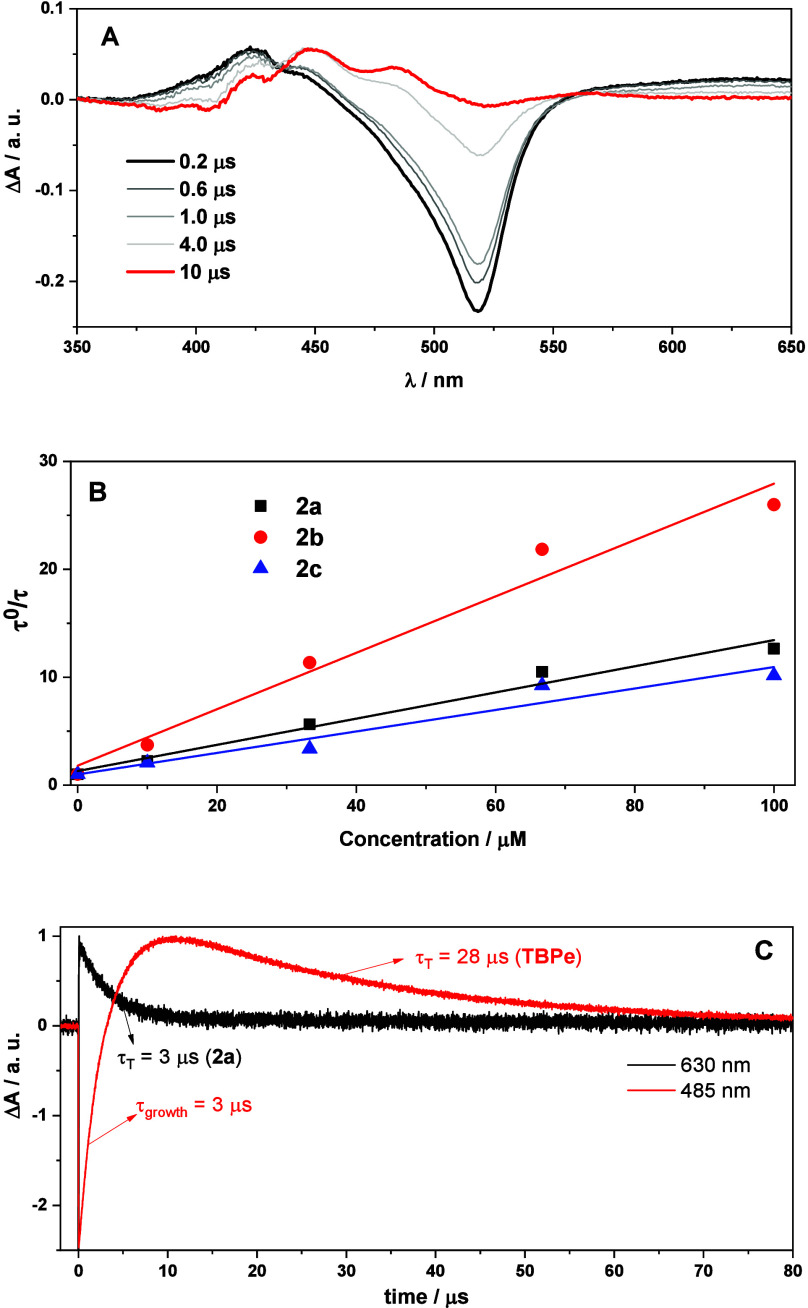
(A) Transient spectra obtained upon LFP (λ_exc_ = 532 nm, 685 mW/cm^2^) of a mixture of **2a** (10 μM) and **TBPe** (100 μM) in degassed MeCN were recorded at different times after laser pulse. (B) Stern–Volmer plot used for calculation of the corresponding quenching rate constants. (C) Decay kinetics monitored at 630 nm (black) and 485 nm (red) of **2a** (10 μM) in the presence of **TBPe** (100 μM) in degassed MeCN after 532 nm LFP.

Thus, LFP experiments (λ_exc_ = 532 nm; ∼10 ns pulse duration) were performed on a mixture of **2a** and **TBPe**. After 200 ns of laser pulse, the band obtained exhibited a pronounced ground-state bleaching and a broad absorption with maxima at ca. 630 and 455 nm), which was safely assigned to the triplet excited state (Figure 3A, black line).^30^ The same trend was observed in the case of **2b**/**TBPe** and **2c**/**TBPe** mixtures (Figure S15). The triplet lifetimes (τ_T_) fitted perfectly to a monoexponential decay and were determined as 47, 57, and 31 μs for **2a**–**2c**, respectively. Hence, τ_T_ gradually decreased upon the addition of increasing amounts of **TBPe** as a quencher (Figure S16). Figure 3B shows the Stern–Volmer relationship,^41^ where the TTEnT rate constants were obtained as 3.5 × 10^9^ M^–1^ s^–1^ (**2a**), 2.6 × 10^9^ M^–1^ s^–1^ (**2b**), and 3.1 × 10^9^ M^–1^ s^–1^ (**2c**), indicating that TTEnT was very efficient. In the presence of **TBPe**, new intense signals appeared at 485 and 455 nm, 10 μs after the laser pulse, which were safely ascribed to the triplet state of **TBPe** (Figure 3A, red line).^40^ Indeed, the **2a** triplet decay at 630 nm (τ_T_ = 3 μs) was concomitant with generation of the **TBPe** triplet at 485 nm (τ_growth_ = 3 μs), unambiguously demonstrating that quenching and photoproduct formation occurred with identical rates (Figures 3C and S17).

### 2.5 Triplet–Triplet Annihilation (TTA)

**Figure 4:**
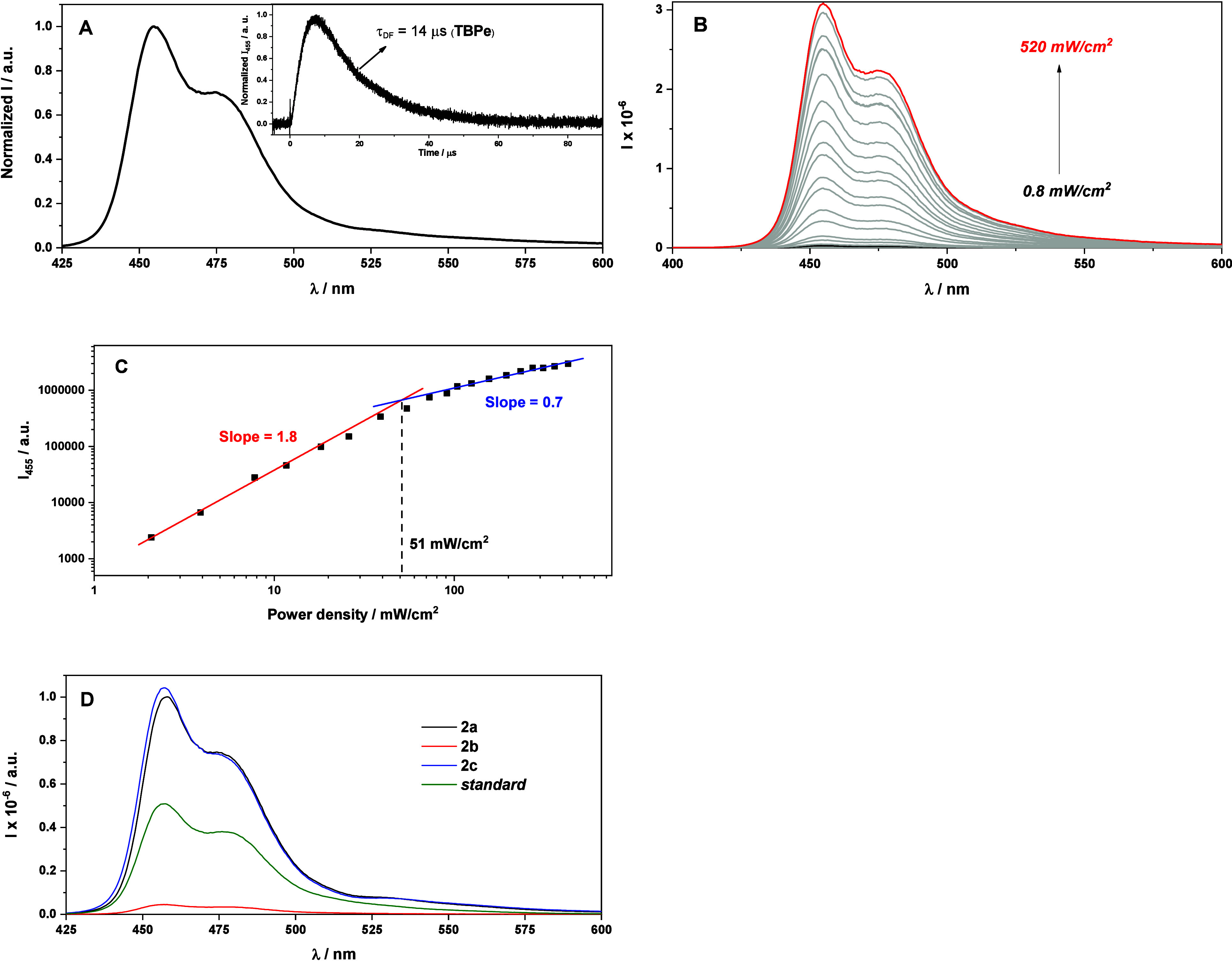
(A) Emission spectrum (λ_exc_ = 532 nm) of a mixture of **2a** (10 μM) and **TBPe** (100 μM) in N_2_/MeCN recorded at 1 μs after laser pulse. Inset: Kinetic decay of delayed ^**1**^TBPe* at 455 nm. (B) Emission spectra (λ_exc_ = 532 nm) of a mixture of **2a** (10 μM) and **TBPe** (100 μM) in N_2_/MeCN recorded at 1 μs after laser pulse with increasing laser intensity. (C) Double-logarithmic plot of the delayed fluorescence intensity as a function of the excitation power for the case of the **2a**/**TBPe** pair. (D) Emission spectra (λ_exc_ = 532 nm) of a mixture of **2**/**TBPe** (∼100 μM) (black, red and blue lines) in N_2_/MeCN recorded at 1 μs after laser pulse. The green line corresponds to the standard.

**Figure 5:**
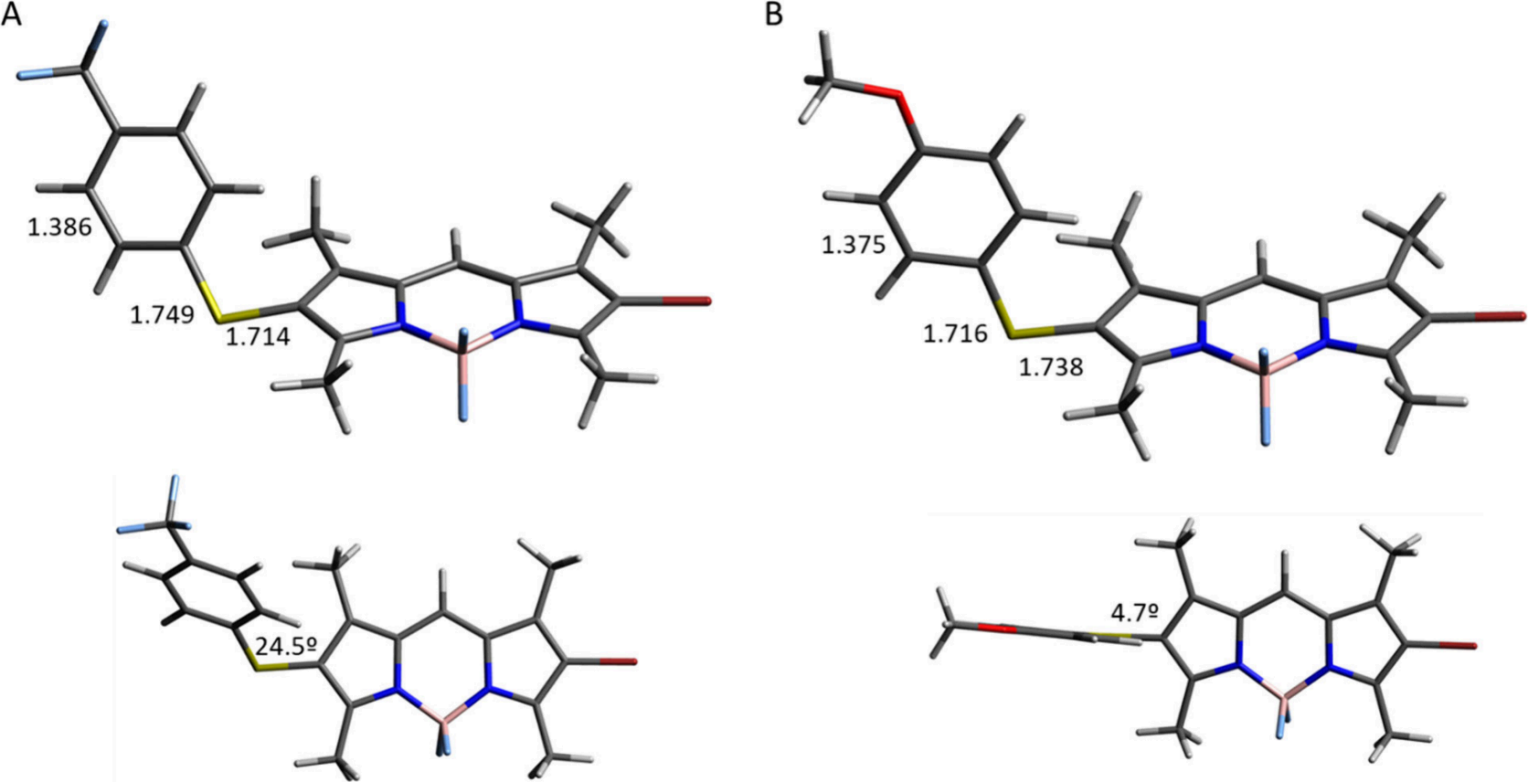
Computed S_1_ minima obtained using the B2PLYP functional and def2-TZVP basis set. (A) Side and top views of **2a**. (B) Side and top views of **2b**. Distances are in angstroms and angles in degrees.

Once the TTEnT process was well-established, we focused our attention on the annihilation step. To observe the resultant formation of the **TBPe** delayed fluorescence (^**1**^TBPe*), a deaerated MeCN solution of a mixture of **2**/**TBPe** was submitted to LFP with an excitation of 532 nm. The upconverted ^**1**^TBPe* was detected by the presence of an emission band with a maximum at 455 nm when **2a** was used as a sensitizer (Figure 4A). Similar results were found for **2b**/**TBPe** and **2c**/**TBPe** couples (Figure S18). Gratifyingly, estimation of the upconverted ^**1**^TBPe* lifetime was obtained from a monoexponential fit of the temporal profile at 455 nm. The value was 14 μs (inset of Figure 5A). By definition,^42^ a p-type delayed fluorescence depends on the TTA event where two triplet excited states (i.e., ^**3**^TBPe* + ^**3**^TBPe*) interact in order to populate the delayed emission (i.e., ^**1**^TBPe*). Accordingly, its lifetime should be approximately half that of its precursor (the triplet excited state τ_T_ = 28 μs for **TBPe**; Figure 4C) to warrant that the overall process is biphotonic. From these data, it was clear that **2**/**TBPe** pairs fulfilled all of the above-mentioned criteria.

To further analyze the TTA-UC characteristics, measurements of both the UC intensity and UC emission efficiency (η_UC_) were performed as a function of the incident laser power density. The UC intensity of **2a**–**2c** increased with increasing laser power (Figures 4B and S19). At low excitation power, the UC intensity of the sensitizers showed a quadratic dependence on the excitation power, which was adjusted to a linear fit with an increase in the excitation power. A typical characteristic of the TTA-UC system shows a slope factor (photonic order) of about 2 in the nonsaturated regime, which approaches 1 upon saturation. The region where the TTA process is dominant could be determined by the threshold excitation intensity (*I*_th_), which was estimated from the crossing point of the extrapolated slopes between the quadratic and linear range of Figure 4C (see also Figure S20). The *I*_th_ values of **2a** and **2c** were 51 and 34 mW/cm^2^, respectively (in the case of **2b**, it could not be determined). These results confirmed that the long triplet lifetime of **2a** and **2c** contributed to the small *I*_th_ because *I*_th_ is inversely proportional to ϕ_TTET_.^43^

η_UC_ was determined using eq 2:
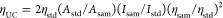
2where η, *I*, *A*, and *n* are the UC emission efficiency, integrated upconverted emission intensities, absorbance of the triplet sensitizers at 532 nm, and solvent refractive index, respectively. The subscripts std and sam indicate the standard and sample, respectively. A TTA-UC system based on a BODIPY derivative as the sensitizer and **TBPe** as the annihilator in MeCN was used as the standard (η_std_ = 2.7%).^30^ Therefore, the η_UC_ values were found to be 8.2%, 0.4%, and 7.9% for **2a**–**2c**, respectively, at an excitation power of 250 mW/cm^2^ (Figure 4D). The presence of electron-withdrawing groups such as CF_3_ or CN in the thiophenol moiety could enhance the induction effect, positively affecting the efficiency of the UC process. In addition, the triplet population of sensitizers **2a** and **2c** was higher than that of **2b** (*vide supra*), increasing the probability of interacting with the annihilator before triplet energy loss.

### 2.6 Computational Studies

Despite the fact that BODIPY dyes have been extensively used in many applications and studied through different computational methodologies, a poor agreement between the experimental data and most of the computational approaches has been found.^44^*Ab initio* approaches have been more successful,^45^ although their extended use is prevented by a significant increase in computational cost. In this context, recent reports^46^ have introduced alternative methods to obtain a reasonable agreement with the experiment at a fraction of the cost.

To provide a rationale for the above-mentioned data, a series of theoretical calculations within the framework of the (time-dependent) densty functional theory methodology were performed. We thus explored the properties of **2a** and **2b**, which presented the most different behavior. From the observed experimental data, we first aimed to investigate the performance of some of the previously reported functionals such as the optical properties of these new BODIPYs. For this, we computed the absorption spectrum of **2a** in hexane using the ωB97XD, CAM-B3LYP, B3LYP, B2PLYP, and PBE0 functionals. Not surprisingly, only a qualitative agreement between our calculations and the experimental values was found (Table S1). The best results were obtained with the B2PLYP functional and def2-TZVP basis set. Within this functional, we also computed **2b** and checked the solvent effect by exchanging hexane with dimethyl sulfoxide (DMSO; Table S2). In agreement with the experimental results, a small red shift was found when moving from **2a** to **2b** and a blue shift when exchanging hexane with DMSO in both compounds. Therefore, relative trends were well reproduced despite some discrepancies between the experimental and computed optical properties.

Related to the observed differences in η_UC_, we explored the distributions of both singlets and triplets in the Franck–Condon region. A similar picture was displayed for both compounds (Table S3). The experimental ϕ_ISC_ values found for **2a** and **2b** (see section 2.3) in combination with the ϕ_F_ values reported in Table 1 suggested different deactivation channels for **2a** and **2b**. The emission was affected by the solvent in both cases, but clearly **2b** possessed a less emissive behavior, pointing out a nonradiative decay channel operating mainly in this compound. To check this hypothesis, the excited-state minima for both compounds were computed. The corresponding geometries are shown in Figure 5. Interestingly, a different disposition of the thiophenol moiety was observed in both compounds. As a matter of fact, the particular donating ability of the substituent in the phenyl moiety (CF_3_ in **2a** and MeO in **2b**) caused a slight but significant change in the geometry computed for the minimum in S_1_. In the case of **2b**, a more pronounced quinoid structure was found in the phenyl moiety and an almost perpendicular disposition of the phenyl and BODIPY moieties (dihedral angle of 4.7°). In contrast, **2a** features a dihedral angle of 24.5°, in agreement with the higher double-bond character of the S–C_BODIPY_ bond (1.714 Å in **2a** vs 1.738 Å in **2b**). This increment of the conformational freedom between the phenyl and BODIPY moieties found for **2b** may cause a “loose bolt” effect,^47^ which contributed to an efficient internal conversion because flexible groups contribute to a nonradiative decay. This unproductive pathway has already been reported in other BODIPY derivatives.^48^ In addition, the Min in S_1_ for **2a** was located 2.19 eV above the ground state, whereas the energy gap was 1.99 eV for **2b**, suggesting again a more efficient nonradiative internal conversion in the case of **2b**. This hypothesis was in agreement with the experimental results of **2b** because both smaller η_UC_ and ϕ_F_ values were found in comparison to those of **2a**.

## 3 Conclusions

In conclusion, a new class of triplet-state sensitizers based on BODIPY was prepared. These new compounds presented an asymmetric substitution, with particular optical properties that were studied using steady-state UV–vis absorption spectroscopy, fluorescence spectroscopy, and nanosecond time-resolved transient absorption spectroscopy. Thus, they showed absorbance maxima in the range of 519–530 nm, which could be beneficial for working with low-price and durable green light sources. Due to the internal heavy-atom effect, the fluorescence quantum yields and emission lifetimes of the bromoBODIPY dyes were lower than those of the unsubstituted BODIPY chromophore. Population of long-lived triplet states of the corresponding bromoBODIPY dyes was found to be very efficient. These new asymmetric sensitizers were used for TTA-UC, using **TBPe** as an annihilator. Intermolecular TTEnT was confirmed experimentally by convenient quenching experiments with high rate constants. The resulting delayed fluorescence of **TBPe** from the TTA-UC process was clearly observed using these new BODIPY dyes as sensitizers. The efficiency of the UC process resulted in a higher value of 8% for the **2a**/**TBPe** pair, which, in principle, would be sufficient to apply this system as a photocatalyst to drive challenging photoredox reactions. Computational data allowed one to provide a potential explanation of the observed results. Overall, this study is useful for the development of new organic triplet photosensitizers based on BODIPY dyes. To further enhance the UC efficiency, ongoing investigations aimed at constructing novel triplet sensitizers are in progress, whereas a new pathway of annihilator design and synthesis is still in urgent need, but this issue is beyond the current investigation.

## 4 Experimental Section

### 4.1 Synthetic Procedure

Target compounds **2a**–**2c** were synthesized following a modified methodology described in ref (30). First, an oxidative addition of the corresponding thiophenol to the starting 3,5-dimethylpyrrole-2-carboxaldehyde was carried out. Then, condensation of the corresponding product with 2,4-dimethylpyrrole in the presence of HBr as a catalyst in methanol (MeOH) was performed. The next step consisted of formation of the corresponding difluoroborate complexes. Finally, NBS treatment led to bromine addition. The different reaction steps are explained as follows:

#### Synthesis of Compounds **3**

The corresponding thiophenol (1 mmol, 1 equiv), 3,5-dimethylpyrrole-2-carboxaldehyde (338.0 mg, 2.5 mmol, 2.5 equiv), and CuI (476.1 mg, 2.5 mmol, 2.5 equiv) were mixed in 2 mL of DMSO and placed in a round-bottomed flask under argon at 110 °C for 20 h. Water was added to precipitate the products, and the mixture was filtered. Next, the solid was dissolved in dichloromethane (DCM) and again filtered to remove the insoluble CuI. The organic phase was washed with brine, dried over anhydrous MgSO_4_, filtered, and concentrated in vacuum. The crude was then purified via silica gel flash column chromatography using *n*-hexane/ethyl acetate as an eluent. Isolated yields were found to be 35%, 33%, and 33% for **3a**–**3c**, respectively, as brown solids.

##### 3,5-Dimethyl-4-[[4-(trifluoromethyl)phenyl]thio]-1*H*-pyrrole-2-carbaldehyde (**3a**)

^1^H NMR (400 MHz, CDCl_3_): δ 10.08 (s, 1H), 9.61 (s, 1H), 7.44 (d, *J* = 8.3 Hz, 2H), 7.05 (d, *J* = 8.2 Hz, 2H), 2.35 (s, 3H), 2.30 (s, 3H). ^13^C NMR (101 MHz, CDCl_3_): δ 177.0, 143.9, 143.2, 138.0, 129.1, 127.2 (q, *J* = 32.6 Hz), 125.9 (q, *J* = 3.9 Hz), 125.0, 124.3 (q, *J* = 271.4 Hz), 109.2, 12.1, 9.4. ^19^F NMR (377 MHz, CDCl_3_): δ −62.3.

##### 4-[(4-Methoxyphenyl)thio]-3,5-dimethyl-1*H*-pyrrole-2-carbaldehyde (**3b**)

^1^H NMR (400 MHz, CDCl_3_): δ 9.97 (s, 1H), 9.55 (s, 1H), 6.99 (d, *J* = 8.9 Hz, 2H), 6.78 (d, *J* = 9.0 Hz, 2H), 3.76 (s, 3H), 2.36 (s, 3H), 2.32 (s, 3H). ^13^C NMR (101 MHz, CDCl_3_): δ 176.8, 157.9, 142.9, 138.0, 128.9, 128.7, 128.1, 114.8, 112.4, 55.5, 12.2, 9.6.

##### 4-[(5-Formyl-2,4-dimethyl-1*H*-pyrrol-3-yl)thio]benzonitrile (**3c**)

^1^H NMR (400 MHz, CDCl_3_): δ 10.46 (s, 1H), 9.61 (s, 1H), 7.47 (d, *J* = 8.6 Hz, 2H), 7.03 (d, *J* = 8.5 Hz, 2H), 2.36 (s, 3H), 2.29 (s, 3H). ^13^C NMR (101 MHz, CDCl_3_): δ 177.0, 146.0, 143.5, 138.1, 132.4, 129.0, 125.1, 118.9, 108.2, 108.0, 12.0, 9.3.

#### Synthesis of Compounds **4**

To a solution of 36 μL of 2,4-dimethylpyrrole (0.35 mmol, 1 equiv) and the corresponding **3** in 5 mL of MeOH was added 0.5 mL of HBr(conc.). The mixture was stirred for 2 h at room temperature; the precipitate was filtered off, washed with cold MeOH, and dried with a vacuum at room temperature. Isolated yields were found to be 67%, 60%, and 67% for **4a**–**4c**, respectively, as orange solids.

##### (*Z*)-2-[[3,5-Dimethyl-4-[[4-(trifluoromethyl)phenyl]thio]-1*H*-pyrrol-2-yl]methylene]-3,5-dimethyl-2*H*-pyrrol-1-ium Bromide (**4a**)

^1^H NMR (300 MHz, CDCl_3_): δ 13.59 (s, 1H), 13.53 (s, 1H), 7.46 (d, *J* = 8.3 Hz, 2H), 7.21 (s, 1H), 7.05 (d, *J* = 8.0 Hz, 2H), 2.76 (s, 3H), 2.69 (s, 3H), 2.40 (s, 3H), 2.36 (s, 3H). ^13^C NMR (101 MHz, CDCl_3_): δ 159.8, 157.4, 149.1, 148.7, 142.1, 128.2, 127.8 (q, *J* = 33.5 Hz), 126.15 (q, *J* = 3.8 Hz), 125.7, 125.5, 124.2 (q, *J* = 272.2 Hz), 120.9, 119.2, 115.32, 15.0, 13.1, 12.4, 11.3. ^19^F NMR (282 MHz, CDCl_3_): δ −62.4.

##### (*Z*)-2-[[4-[(4-Methoxyphenyl)thio]-3,5-dimethyl-1*H*-pyrrol-2-yl]methylene]-3,5-dimethyl-2*H*-pyrrol-1-ium Bromide (**4b**)

^1^H NMR (300 MHz, CDCl_3_): δ 13.42 (s, 1H), 13.33 (s, 1H), 7.16 (s, 1H), 7.02 (d, *J* = 8.9 Hz, 2H), 6.78 (d, *J* = 8.9 Hz, 2H), 6.22 (s, 1H), 2.71 (s, 3H), 2.69 (s, 3H), 2.38 (s, 3H), 2.37 (s, 3H). ^13^C NMR (75 MHz, CDCl_3_): δ 158.5, 158.0, 148.8, 147.6, 129.3, 127.6, 126.9, 125.8, 120.6, 119.5, 118.5, 115.0, 55.5, 14.8, 13.4, 12.4, 11.3.

##### (*Z*)-2-[[4-[(4-Cyanophenyl)thio]-3,5-dimethyl-1*H*-pyrrol-2-yl]methylene]-3,5-dimethyl-2*H*-pyrrol-1-ium Bromide (**4c**)

^1^H NMR (400 MHz, CDCl_3_): δ 13.61 (s, 1H), 13.55 (s, 1H), 7.48 (d, *J* = 8.6 Hz, 2H), 7.22 (s, 1H), 7.01 (d, *J* = 8.6 Hz, 2H), 6.29 (s, 1H), 2.76 (s, 3H), 2.67 (s, 3H), 2.41 (s, 3H), 2.35 (s, 3H). ^13^C NMR (101 MHz, CDCl_3_): δ 160.3, 156.9, 149.0, 148.8, 144.3, 132.7, 128.4, 125.6, 125.5, 121.0, 119.4, 118.8, 114.2, 108.8, 15.0, 13.1, 12.5, 11.2.

#### Synthesis of Compounds **1**

A solution of 0.2 mmol (1 equiv) of **4** in 5 mL of DCM was stirred at room temperature. Then, 277 μL (2 mmol, 10 equiv) of triethylamine was added, and immediately 247 μL of boron trifluoride etherate (2 mmol, 10 equiv) was also added. The mixture was stirred for 3 h and then washed three times with water and one time with brine. The organic layer was dried over anhydrous MgSO_4_, filtered, and evaporated to dryness on a rotary evaporator at a reduced pressure. The solid residue was purified by silica gel flash column chromatography using *n*-hexane/DCM as an eluent. Isolated yields were found to be 65%, 57%, and 16% for **1a**–**1c**, respectively, as orange solids.

##### 2-(4-Trifluoromethylphenyl)thio-1,3,5,7-tetramethyl-4,4′-difluoro-4-bora-3a,4a-diaza-*s*-indacene (**1a**)

^1^H NMR (400 MHz, CDCl_3_): δ 7.44 (d, *J* = 8.3 Hz, 2H), 7.17 (s, 1H), 7.09 (d, *J* = 8.2 Hz, 2H), 6.16 (s, 1H), 2.59 (s, 3H), 2.54 (s, 3H), 2.30 (s, 3H), 2.25 (s, 3H). ^13^C NMR (101 MHz, CDCl_3_): δ 161.0, 158.0, 144.3, 144.1, 143.4, 143.4, 135.1, 131.8, 127.2 (q, *J* = 32.7 Hz), 125.93 (q, *J* = 3.8 Hz), 125.3, 124.3 (q, *J* = 271.7 Hz), 120.9, 115.3, 15.2, 12.8, 11.5, 10.6. ^11^B NMR (96 MHz, CDCl_3_): δ 0.82 (t, *J* = 33.3 Hz). ^19^F NMR (282 MHz, CDCl_3_): δ −62.27, −146.21 (q, *J* = 32.6 Hz). HRMS (EI). Calcd for C_20_H_18_BF_5_N_2_S [(M + H)^+^]: *m*/*z* 425.1277. Found: *m*/*z* 425.1264.

##### 2-(4-Methoxyphenyl)thio-1,3,5,7-tetramethyl-4,4′-difluoro-4-bora-3a,4a-diaza-*s*-indacene (**1b**)

^1^H NMR (400 MHz, CDCl_3_): δ 7.12 (s, 1H), 7.04 (d, *J* = 9.0 Hz, 2H), 6.78 (d, *J* = 8.8 Hz, 2H), 6.11 (s, 1H), 3.76 (s, 3H), 2.56 (s, 6H), 2.27 (s, 6H). ^13^C NMR (101 MHz, CDCl_3_): δ 159.4, 158.8, 158.1, 144.2, 143.2, 134.5, 131.9, 128.7, 128.2, 120.7, 120.2, 119.3, 114.9, 55.5, 15.0, 13.1, 11.5, 10.7. ^11^B NMR (96 MHz, CDCl_3_): δ 0.82 (t, *J* = 32.3 Hz). ^19^F NMR (377 MHz, CDCl_3_): δ −146.30 (q, *J* = 32.7 Hz). HRMS (EI). Calcd for C_20_H_21_BF_2_N_2_OS [(M + H)^+^]: *m*/*z* 387.1508, found: *m*/*z* 387.1493.

##### 2-(4-Cyanophenyl)thio-1,3,5,7-tetramethyl-4,4′-difluoro-4-bora-3a,4a-diaza-*s*-indacene (**1c**)

^1^H NMR (400 MHz, CDCl_3_): δ 7.47 (d, *J* = 8.1 Hz, 2H), 7.18 (s, 1H), 7.07 (d, *J* = 8.1 Hz, 2H), 6.18 (s, 1H), 2.59 (s, 3H), 2.52 (s, 3H), 2.30 (s, 3H), 2.24 (s, 3H). ^13^C NMR (101 MHz, CDCl_3_) :δ 161.6, 157.5, 145.7, 144.6, 143.8, 135.3, 132.6, 131.7, 125.4, 121.2, 121.0, 119.0, 114.2, 108.3, 15.2, 12.8, 11.6, 10.6. ^11^B NMR (96 MHz, CDCl_3_): δ 0.80 (t, *J* = 32.6 Hz). ^19^F NMR (377 MHz, CDCl_3_): δ −146.19 (q, *J* = 32.4 Hz). HRMS (EI). Calcd for C_20_H_18_BF_2_N_3_S [(M + H)^+^]: *m*/*z* 382.1355. Found: *m*/*z* 382.1333.

#### Synthesis of Compounds **2**

To a solution of 0.05 mmol (1 equiv) of compound **1** in DCM (2 mL), which was being stirred at room temperature, 10.7 mg (0.06 mmol, 1.2 equiv) of NBS was slowly added. The solution was then stirred under a light bulb for 4 h at room temperature. The reaction crude was washed with 10% Na_2_S_2_O_4_, saturated NaHCO_3_, water, and brine. The organic phase was dried over anhydrous MgSO_4_ and filtered, and the solvent was evaporated using a rotary evaporator at reduced pressure. The residue was purified by silica gel column chromatography using *n*-hexane/DCM as an eluent. Isolated yields were found to be 99%, 68%, and 38% for **2a**–**2c**, respectively, as red solids.

##### 6-Bromo-2-(4-trifluoromethylphenyl)thio-1,3,5,7-tetramethyl-4,4-difluoro-4-bora-3a,4a-diaza-*s*-indacene (**2a**)

^1^H NMR (400 MHz, CDCl_3_): δ 7.46 (d, *J* = 8.1 Hz, 2H), 7.20 (s, 1H), 7.10 (d, *J* = 8.1 Hz, 2H), 2.61 (s, 3H), 2.55 (s, 3H), 2.27 (s, 6H). ^13^C NMR (101 MHz, CDCl_3_): δ 160.23, 156.8, 146.3, 142.8, 140.4, 132.7, 132.4, 127.5 (q, *J* = 32.7 Hz), 126.0 (q, *J* = 3.8 Hz), 125.4, 124.3 (q, *J* = 272.3 Hz), 121.4, 116.7, 110.6, 13.9, 13.1, 11.3, 10.8. ^11^B NMR (96 MHz, CDCl_3_): δ 0.66 (t, *J* = 31.9 Hz). ^19^F NMR (282 MHz, CDCl_3_): δ −62.31, −146.11 (q, *J* = 32.0 Hz). HRMS (EI). Calcd for C_20_H_17_BBrF_5_N_2_S [(M)^+^]: *m*/*z* 503.0382. Found: *m*/*z* 502.7831.

##### 6-Bromo-2-(4-methoxyphenyl)thio-1,3,5,7-tetramethyl-4,4-difluoro-4-bora-3a,4a-diaza-*s*-indacene (**2b**)

^1^H NMR (400 MHz, CDCl_3_): δ 7.12 (s, 1H), 7.06 (d, *J* = 8.9 Hz, 2H), 6.79 (d, *J* = 8.9 Hz, 2H), 3.76 (s, 3H), 2.58 (s, 3H), 2.56 (s, 3H), 2.28 (s, 3H), 2.23 (s, 3H). ^13^C NMR (101 MHz, CDCl_3_): δ 161.1, 158.3, 155.0, 146.2, 139.3, 132.7, 132.0, 129.1, 127.6, 121.0, 115.0, 109.7, 55.5, 13.7, 13.6, 13.3, 11.2, 10.8. ^11^B NMR (96 MHz, CDCl_3_): δ 0.65 (t, *J* = 32.3 Hz). ^19^F NMR (377 MHz, CDCl_3_): δ −146.21 (q, *J* = 32.0 Hz).

##### 6-Bromo-2-(4-cyanophenyl)thio-1,3,5,7-tetramethyl-4,4-difluoro-4-bora-3a,4a-diaza-*s*-indacene (**2c**)

^1^H NMR (400 MHz, CDCl_3_): δ 7.48 (d, *J* = 8.6 Hz, 2H), 7.21 (s, 1H), 7.07 (d, *J* = 8.6 Hz, 2H), 2.62 (s, 3H), 2.53 (s, 3H), 2.28 (s, 3H), 2.26 (s, 3H). ^13^C NMR (101 MHz, CDCl_3_): δ 159.8, 157.5, 146.0, 145.1, 140.8, 132.9, 132.7, 132.3, 125.5, 121.5, 118.9, 115.7, 110.9, 108.5, 13.9, 13.0, 11.3, 10.8. ^11^B NMR (96 MHz, CDCl_3_): δ 0.65 (t, *J* = 31.8 Hz). ^19^F NMR (377 MHz, CDCl^3^): δ −146.16 (q, *J* = 31.9 Hz). HRMS (EI). Calcd for C_20_H_17_BBrF_2_N_3_S [(M + H)^+^]: *m*/*z* 460.0460. Found: *m*/*z* 460.0461.

## Supporting Information Available

Materials and characterization methods, spectroscopic measurements, LFP and computational details, absorption spectra, steady-state, and time-resolved emission spectra, transient absorption spectra, NMR spectra of synthesized compounds, and additional computational results and Cartesian coordinates (PDF)
