# Supplementary material for: Asymmetric BODIPY Dyes Enabling Triplet–Triplet Annihilation Upconversion
Source: ACS Appl Opt Mater. 2024 Jul 13;2(9):1780–9. doi: 10.1021/acsaom.4c00285 (PMC11448374; doi:10.1021/acsaom.4c00285)
Supplement: Supplementary file 1 — ot4c00285_si_001.pdf [file ot4c00285_si_001.pdf]

Supporting information

Asymmetric BODIPY Dyes Enabling Triplet-Triplet Annihilation Upconversion

Daniel Álvarez-Gutiérrez,[a] *Diego Sampedro,*[b] *M. Consuelo Jiménez*[a]* *and Raúl Pérez-Ruiz*[a]*

[a]Departamento de Química, Universitat Politècnica de València, Camino de Vera S/N, 46022, Valencia, Spain

[b]Departamento de Química, Instituto de Investigación en Química de la Universidad de La Rioja (IQUR), Universidad de La Rioja, Madre de Dios, 53, 26006, Logroño, Spain

mcjimene@qim.upv.es ; raupreru@upv.es

Table of contents

This page S1
1- Materials and characterization methods S2
2- Optical properties S3
3- Laser flash photolysis (LFP) S19
4- ^{1}H, ^{13}C, ^{11}B and ^{19}F NMR spectra S30
5- Computational details S50
6- References S58

1- Materials and characterization methods

Reagents and solvents used in this work were purchased from suppliers (Sigma-Aldrich, Indagoo, Fluorochem, TCI), stored properly and employed directly from the container. The BODIPY dyes were synthesized according to the described procedure, while the acceptor **TBPe,** 99 % purity, was bought from Sigma-Aldrich.

Determination of purity and structure confirmation of the literature known products was performed by ^{1}H, ^{13}C, ^{11}B and ^{19}F NMR and high-resolution mass spectrometry (HRMS) in case of unknown products. Nuclear Magnetic Resonance (NMR) spectral data were measured on a Bruker Advance 400 (400 MHz for ^{1}H, 101 MHz for ^{13}C, 128 MHz for ^{11}B and 376 MHz for ^{19}F) spectrometer at 20 °C. Chemical shifts are reported in δ/ppm, coupling constants *J* are given in hertz. Solvent residual peaks were used as internal standard for all NMR measurements. The quantification of ^{1}H cores was obtained from integrations of appropriate resonance signals. The multiplet analysis was done assuming only first-order coupling. Abbreviations used in NMR spectra: s = singlet, d = doublet and q = quartet. High Resolution Mass Spectrometry (HRMS) was performed in the mass facility of SCSIE University of Valencia. Abbreviations used in MS spectra: M = molar mass of target compound, EI = electron impact ionization.

2- Optical properties

Absorption. Ultraviolet–visible spectra (UV–Vis) of the liquid samples were obtained by a JASCO V-650 spectrometer. The samples were placed into quartz cells of 1 cm path length and compound concentrations were fixed as indicated.

Fluorescence. Emission spectra were carried out using an Edinburgh FS5 spectrofluorometer with a standard cuvette holder. The samples were placed into quartz cells of 1 cm path length and compound concentrations were fixed as indicated.

Absolute fluorescence quantum yields (QY_{true}) were measured by using an Edinburgh FS5 spectrofluorometer with a SC-30 integration sphere module. The absolute method requires two measurements: the number of absorbed photons and the number of emitted photons. The number of absorbed photons of a sample is determined by the reduction of the light scatter compared to a blank measurement. The quantum yield calculation is made using a wizard within the operating software. The obtained observed quantum yield (QY_{obs}) was then corrected with emission spectra of a low absorbing solution employing the standard cuvette holder in order to account for reabsorption, according to equation 1:

$$QY^{true} = \frac{QY^{obs}}{1 - a + a \cdot QY^{obs}/100} \quad \text{S1)}$$

where a is the portion of the reabsorbed area when comparing the two emission spectra (concentrated sample with integration sphere and diluted sample with standard cuvette holder).

Fluorescence lifetimes were carried out in an EasyLife X Lifetime Fluorescence Spectrometer connected to a temperature control system working with liquid refrigeration. Samples were placed

in a 3 mL quartz cuvette and irradiated with a 407 nm LED as energy source. The wavelength under 475 nm was filtered with a long pass filter. Lifetimes were obtained using a wizard within the software, employing a reconvolution fit with the corresponding signal decay and IRF (instrument response function).

Phosphorescence spectrum was obtained using an Edinburgh FS5 spectrofluorometer with a SC-70 Liquid Nitrogen Dewar module in order to record samples in quartz tubes (4 mm I.D.) under crystalized EtOH at 77 K.

Absorption and emission spectra

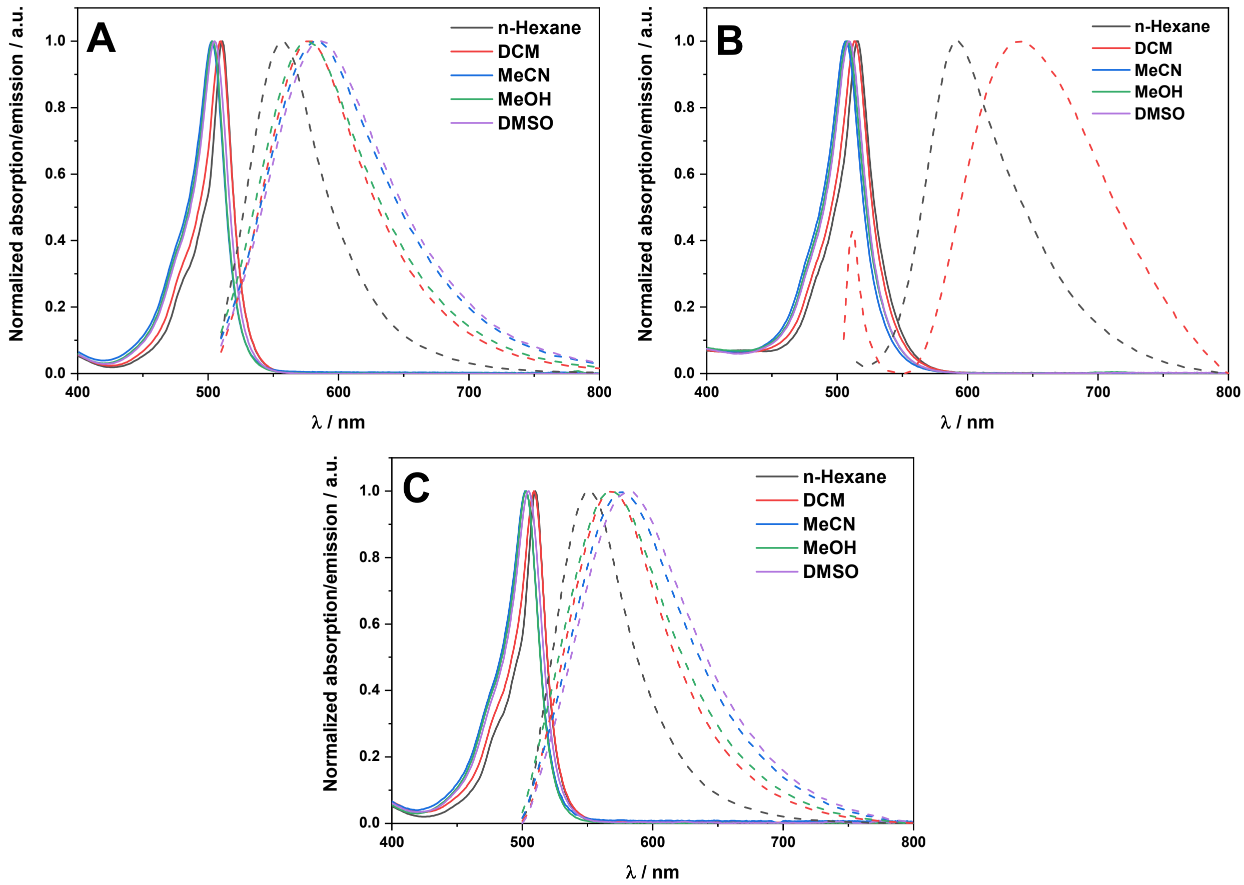

Figure S*1*. Normalized absorption (solid lines) and emission (dashed lines) spectra of 10 μM **1a** (**A**), **1b** (**B**) and **1c** (**C**) in different solvents. Black: n-hexane, red: DCM, blue: MeCN, green: MeOH and purple: DMSO.

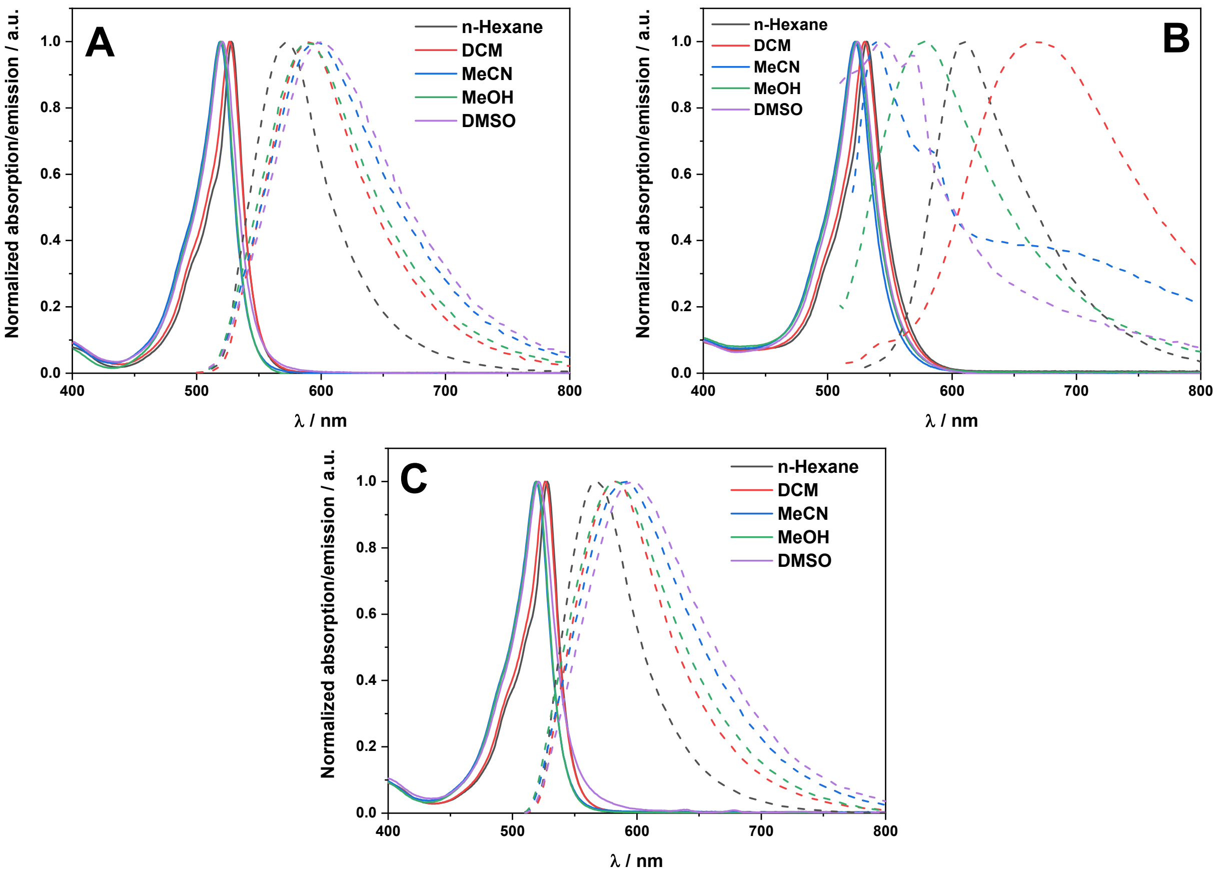

Figure S2. Normalized absorption (solid lines) and emission (dashed lines) spectra of 10 μM **2a** (**A**), **2b** (**B**) and **2c** (**C**) in different solvents. Black: n-hexane, red: DCM, blue: MeCN, green: MeOH and purple: DMSO.

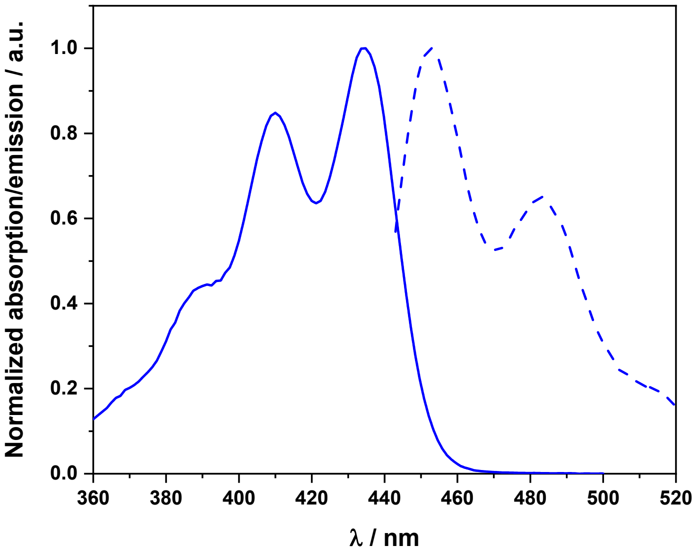

Figure S3. Normalized absorption (solid line) and emission (dashed line) spectra of 10 μM **TBPe** in MeCN.

Phosphorescence spectra

The wavelength at which the emission is 10 % of the maximum intensity is employed to calculate the triplet energy of the sensitizers.

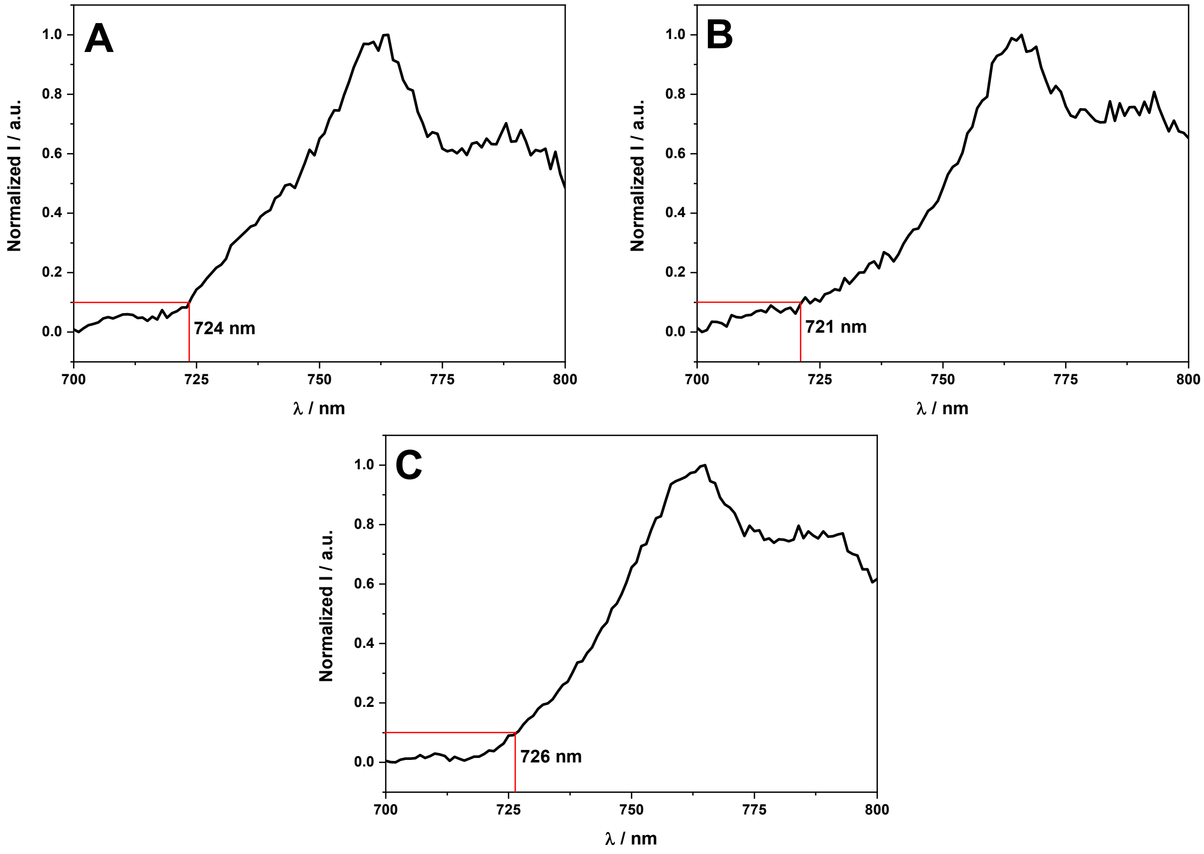

Figure S4. Normalized emission spectra of 10 μM **2a** (**A**), **2b** (**B**) and **2c** (**C**) in EtOH matrix at 77 K. The wavelength at 10 % of emission intensity is indicated.

Absolute fluorescence quantum yields

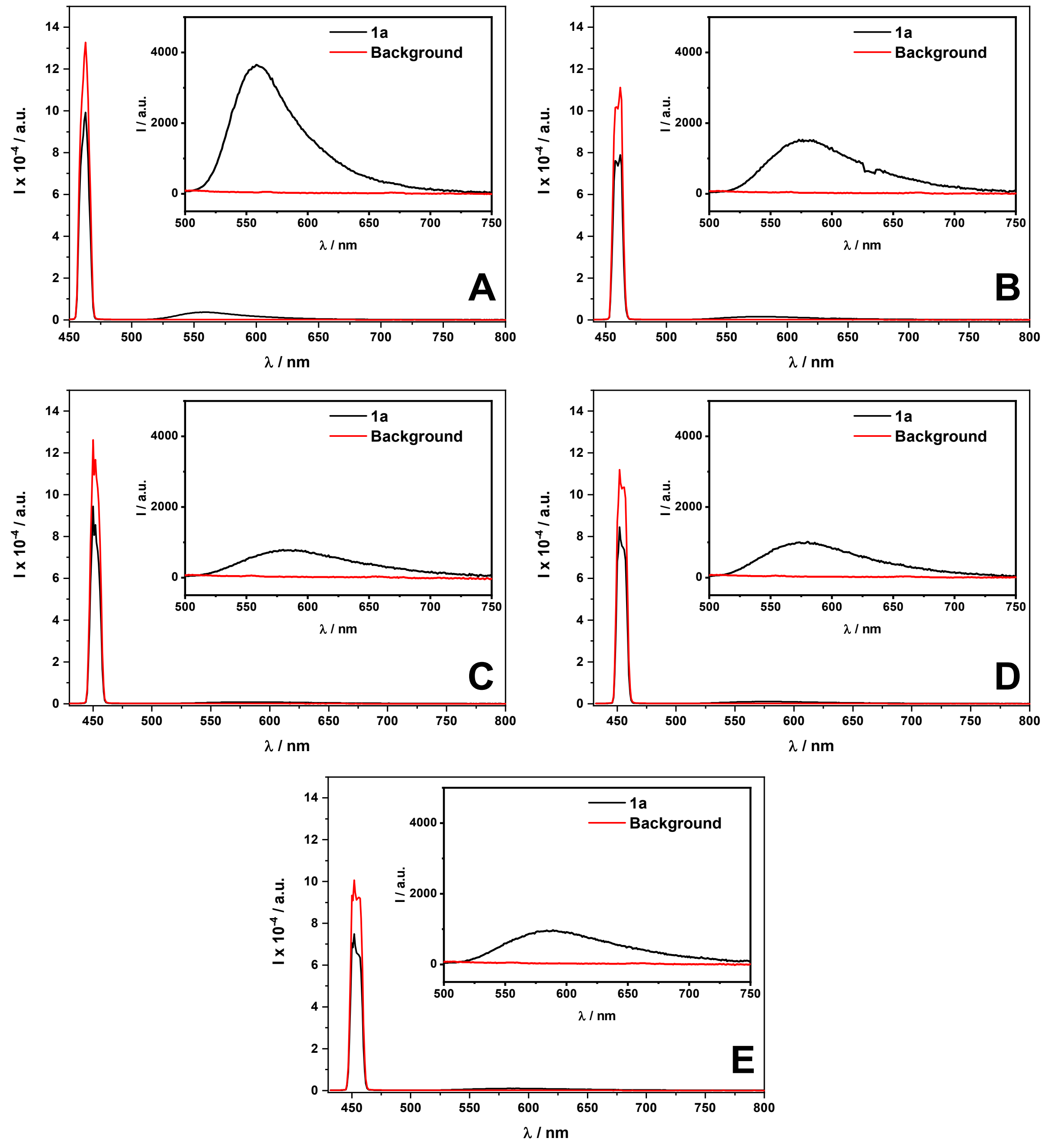

Figure S5. Measurements of absolute fluorescence quantum yield of 10 μM solution of **1a** in different solvents. A: n-hexane, B: DCM, C: MeCN, D: MeOH and E: DMSO. The scatter of the

lamp with the blank (cuvette with solvent) is shown in red and the scatter and emission of **1a** in black.

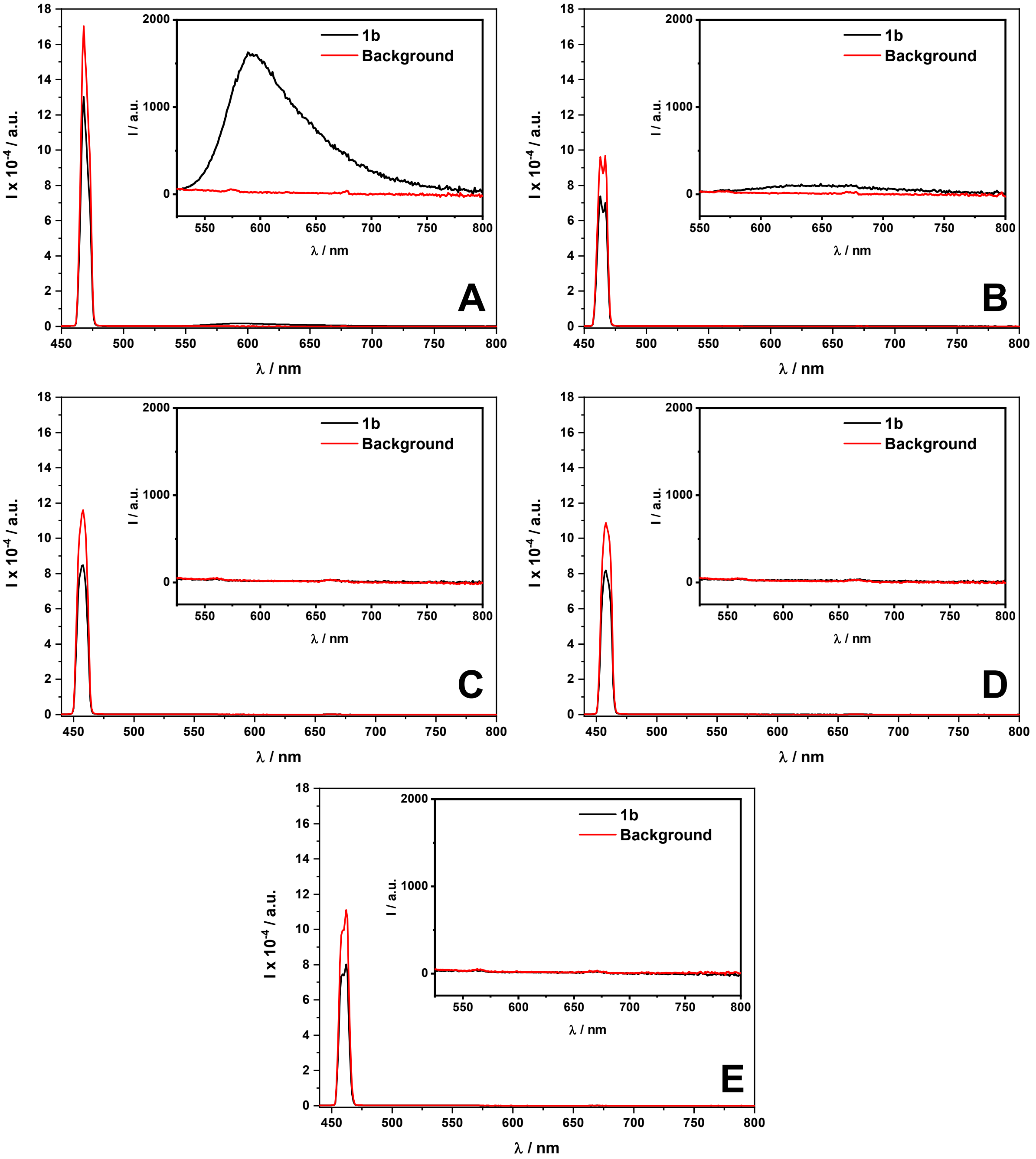

Figure S6. Measurements of absolute fluorescence quantum yield of 10 μM solution of **1b** in different solvents. A: n-hexane, B: DCM, C: MeCN, D: MeOH and E: DMSO. The scatter of the

lamp with the blank (cuvette with solvent) is shown in red and the scatter and emission of **1b** in black.

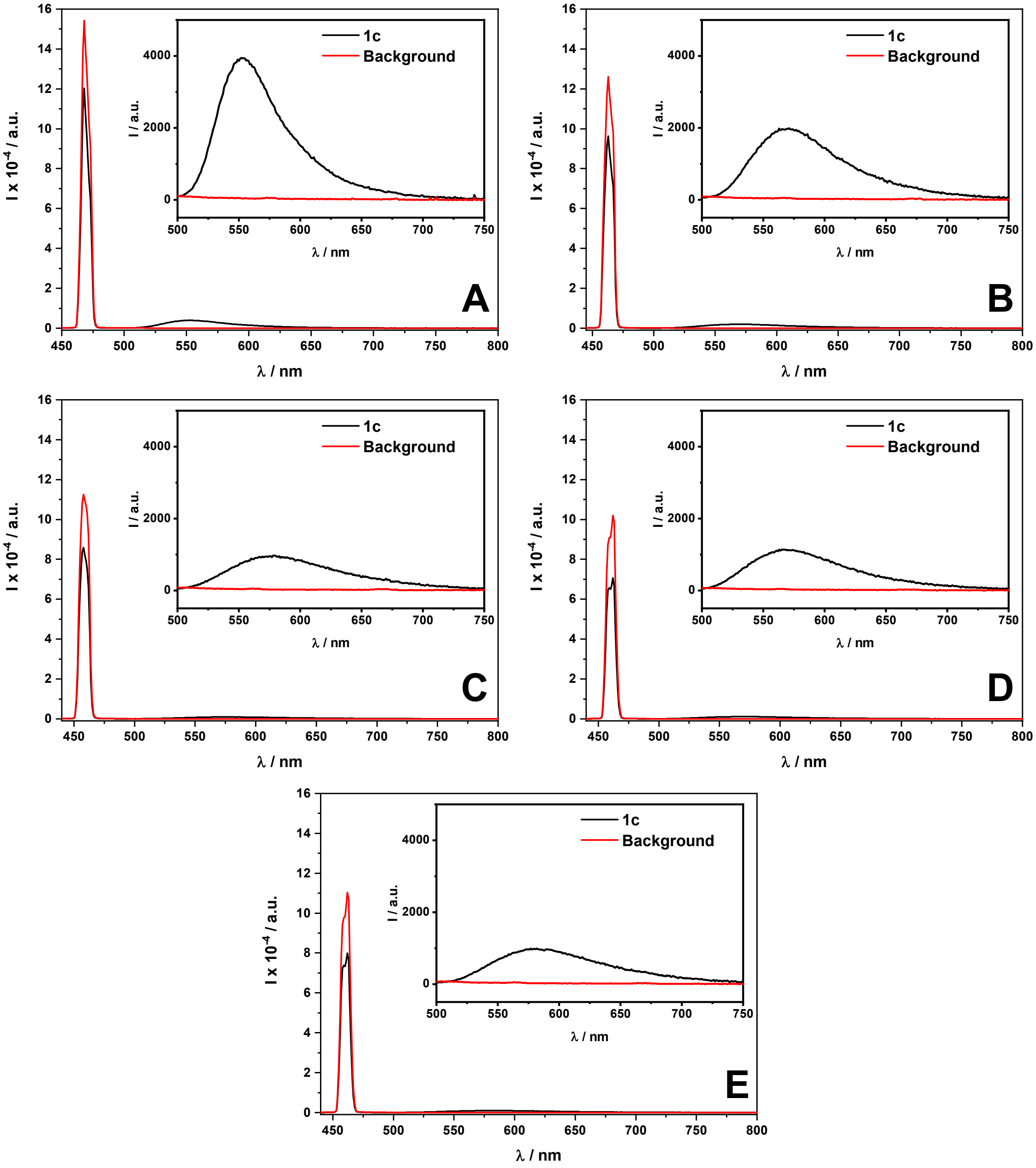

Figure S7. Measurements of absolute fluorescence quantum yield of 10 μM solution of **1c** in different solvents. A: n-hexane, B: DCM, C: MeCN, D: MeOH and E: DMSO. The scatter of the lamp with the blank (cuvette with solvent) is shown in red and the scatter and emission of **1c** in black.

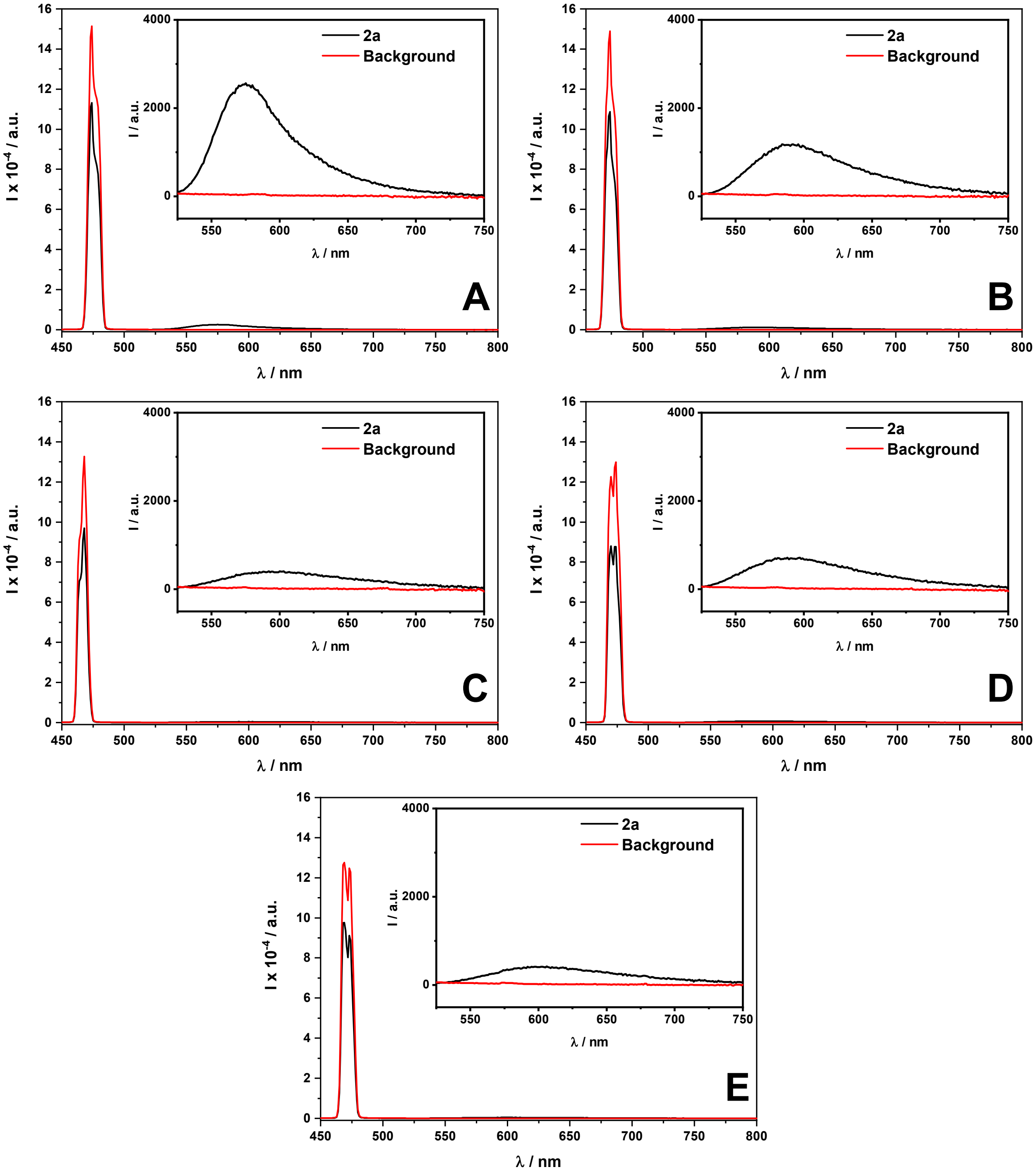

Figure S8. Measurements of absolute fluorescence quantum yield of 10 μM solution of **2a** in different solvents. A: n-hexane, B: DCM, C: MeCN, D: MeOH and E: DMSO. The scatter of the lamp with the blank (cuvette with solvent) is shown in red and the scatter and emission of **2a** in black.

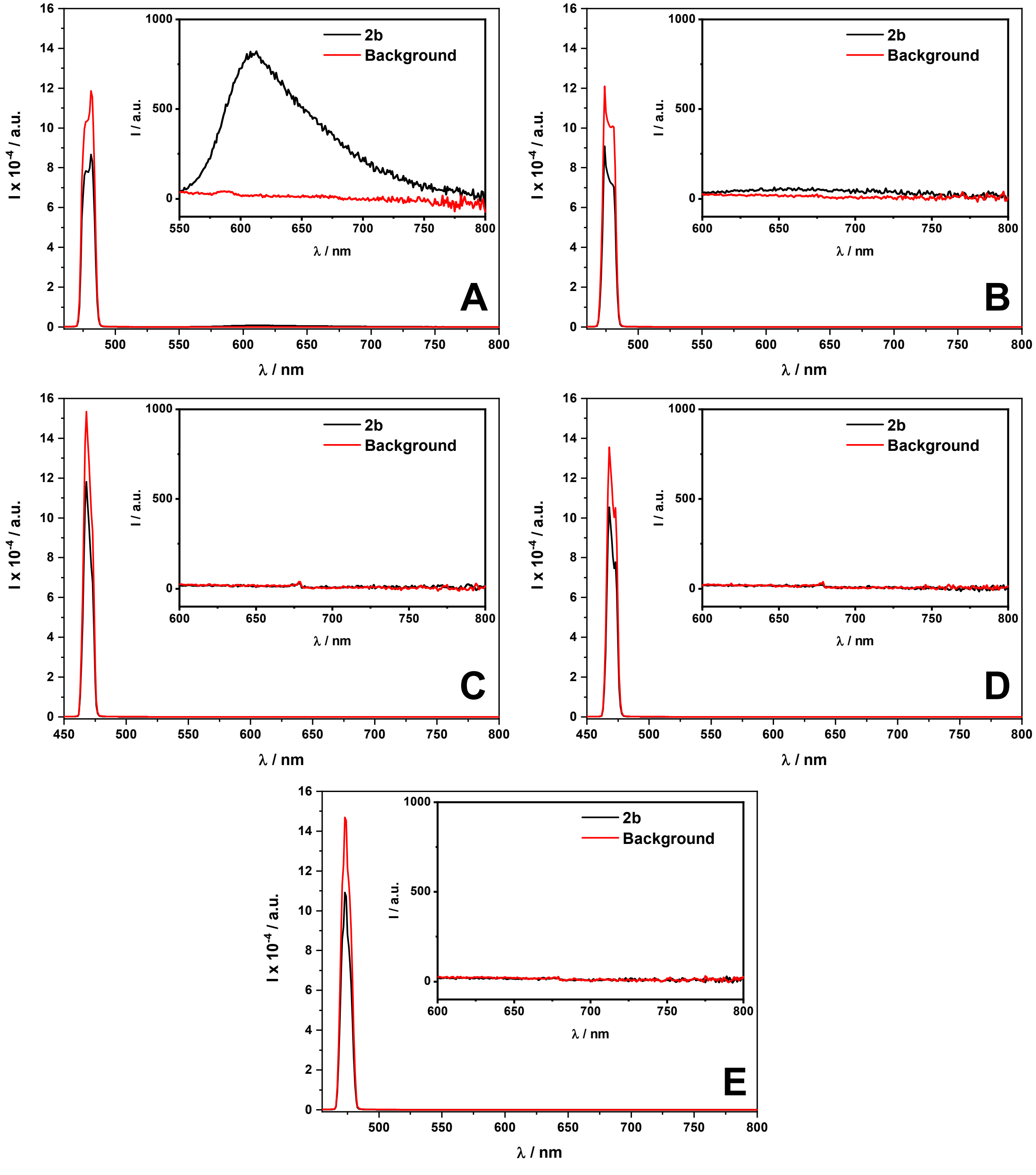

Figure S9. Measurements of absolute fluorescence quantum yield of 10 μM solution of **2b** in different solvents. A: n-hexane, B: DCM, C: MeCN, D: MeOH and E: DMSO. The scatter of the lamp with the blank (cuvette with solvent) is shown in red and the scatter and emission of **2b** in black.

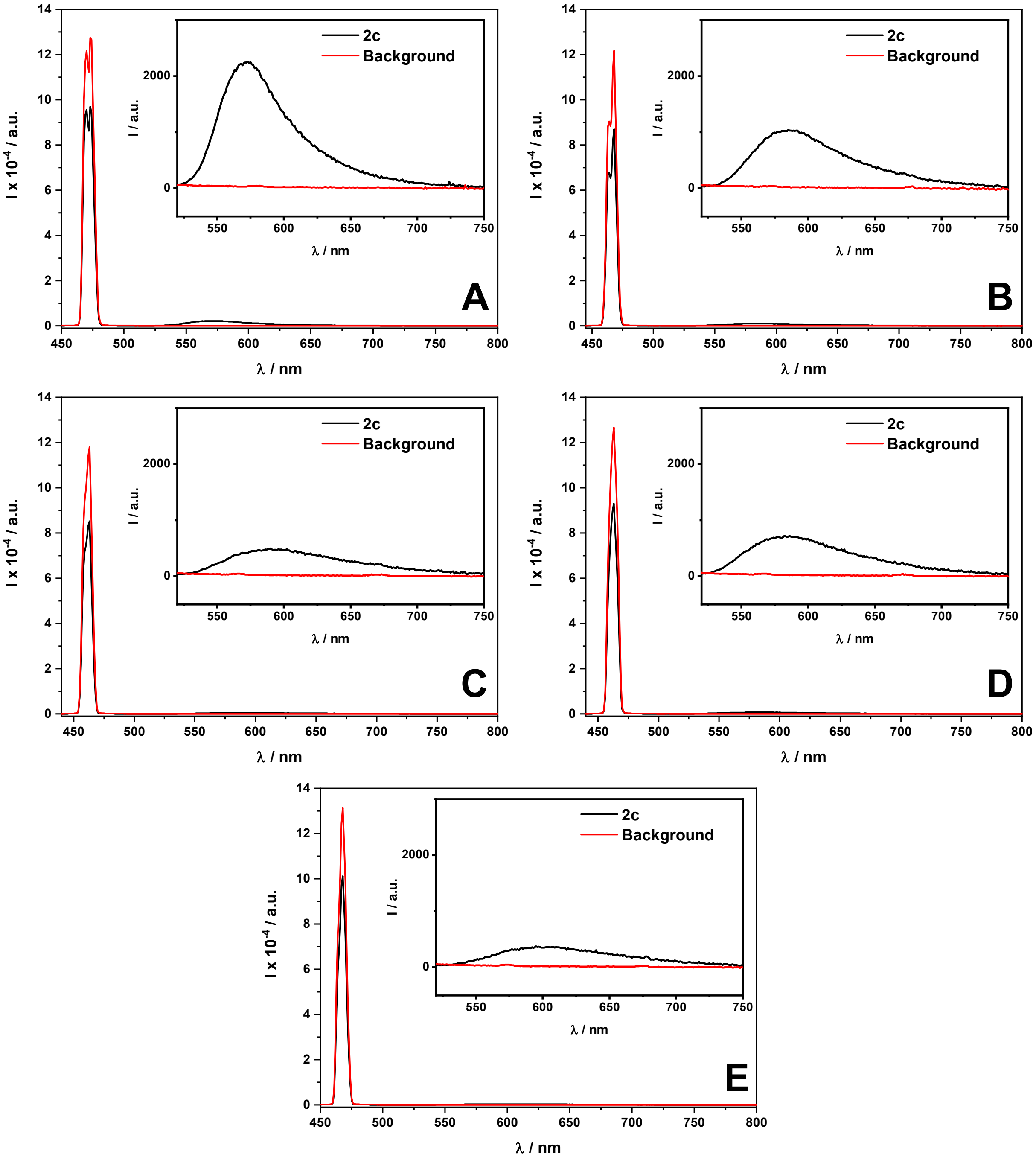

Figure S10. Measurements of absolute fluorescence quantum yield of 10 μM solution of **2c** in different solvents. A: n-hexane, B: DCM, C: MeCN, D: MeOH and E: DMSO. The scatter of the lamp with the blank (cuvette with solvent) is shown in red and the scatter and emission of **2c** in black.

Fluorescence lifetimes

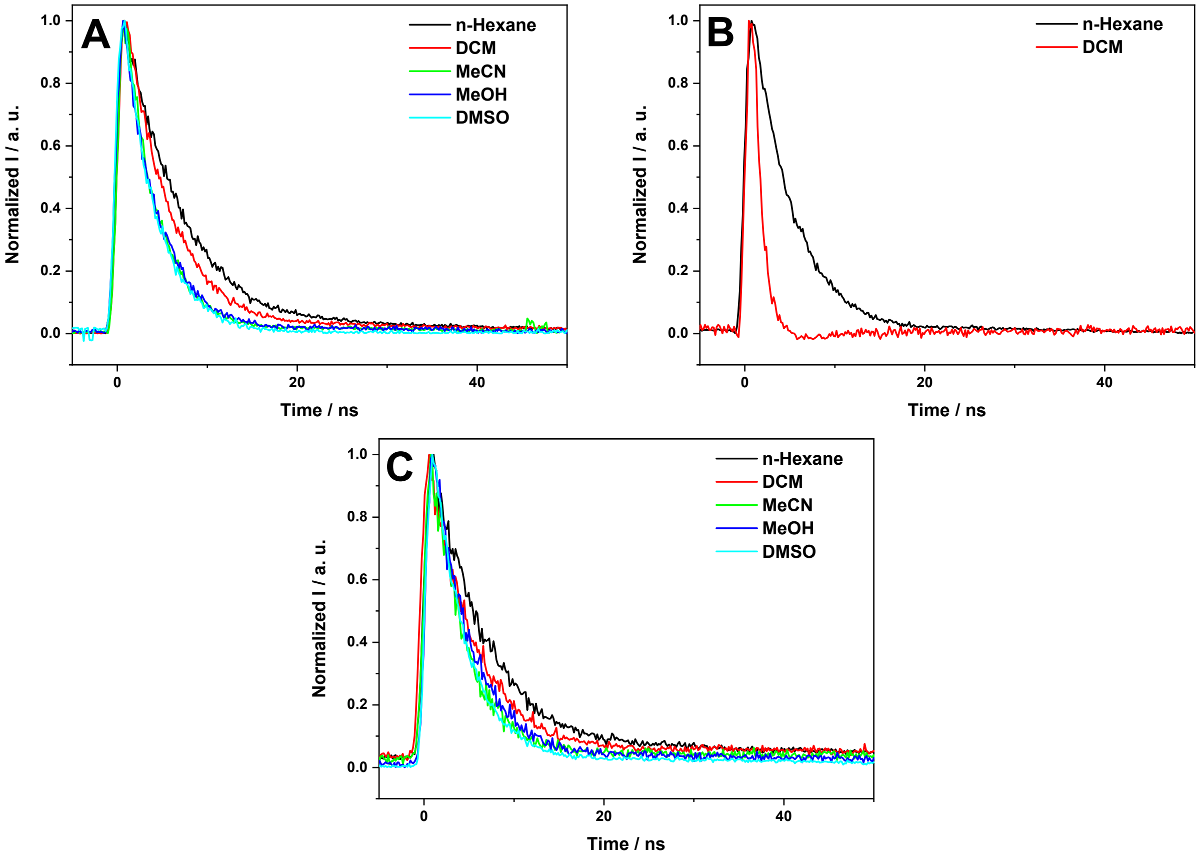

Figure S11. Fluorescence decay traces of 10 μM **1a** (**A**), **1b** (**B**) and **1c** (**C**) in different solvents.

Black: n-hexane, red: DCM, blue: MeCN, green: MeOH and purple: DMSO.

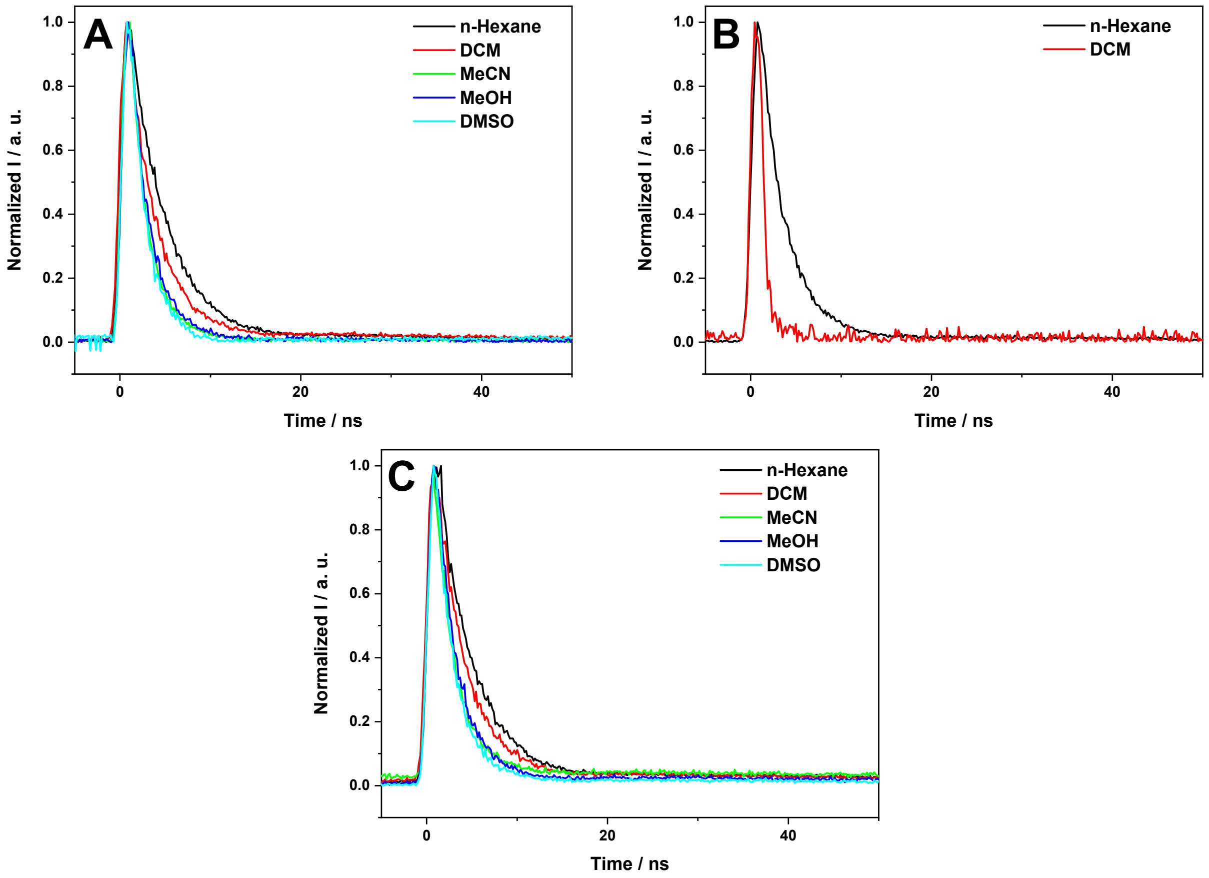

Figure S12. Fluorescence decay traces of 10 μM **2a** (**A**), **2b** (**B**) and **2c** (**C**) in different solvents. Black: n-hexane, red: DCM, blue: MeCN, green: MeOH and purple: DMSO.

Photostability

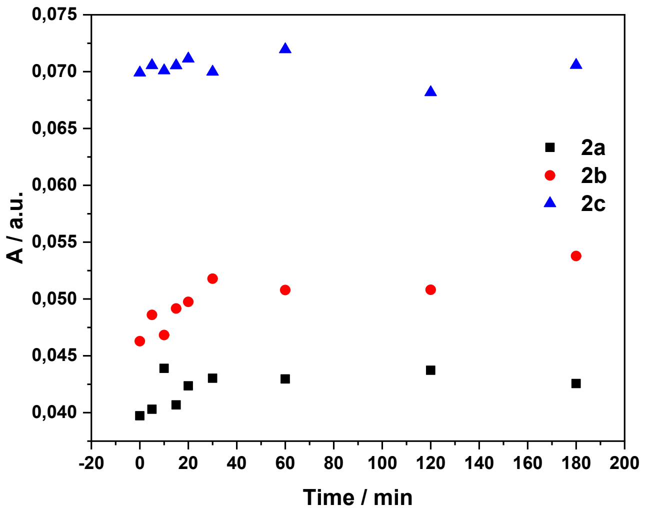

Figure S13. Absorption at the maximum peak of 10 µM solutions of **2a** (black squares), **2b** (red circles) and **2c** (blue triangles) in degassed MeCN after irradiation at 522 nm in a photoreactor at different times.

3- Laser flash photolysis (LFP)

Transient absorption spectra, delayed fluorescence and time-resolved spectra were performed by LFP using The LP980–KS Laser Flash Photolysis Spectrometer (from Edinburgh Instruments) which is a combined system for the measurement of laser induced transient absorption, emission kinetics and spectra, with the ability to automatically convert and fully analyse the kinetic and spectral information. The pump is an INDI Quanta-Ray Nd:YAG laser equipped with a primoSCAN BB optical parametric oscillator (OPO) from SPECTRA PHYSICS®. The probe pulse is longer than the recorded time window of a measurement, and a monochromator (TMS302-A, grating 150 lines mm^{-1}) disperses the probe light after it passed the sample. The probe light can be then passed on to a PMT detector (spectral S5 range 200–870 nm) to obtain the temporal resolved picture. All components are controlled by the software L900 provided by Edinburgh. For our delayed emission measurements, the probe shutter is closed so that no light from the Xe lamp is exciting the sample, and the laser is only used as a light source. To photolyze our samples, a 532 nm monowavelength was employed, ensuring that only the 2 chromophore absorbs the excited photons. The data have been acquired as an average of several shots to improve the signal-to-noise ratio.

Stern-Volmer quenching experiments were conducted to determine the triplet energy transfer rate (k_{TTEnT}, also kq) of **2** to **TBPe** according to equation S2, with τ as the lifetime, [Q] as the quencher concentration and K_{SV} as the Stern-Volmer constant. The index 0 is used in the absence of quencher.

$$\frac{\tau^0}{\tau} = 1 + k_q\tau^0[Q] = 1 + K_{SV}[Q]\text{S2)}$$

ISC quantum yields (ϕ_{ISC})

A previously reported method was used to calculate the ISC quantum yields (ϕ_{ISC}) of the sensitizers **2a, 2b** and **2c**.[1] This method can only be applied where the triplet state does not absorb (triplets of **2a, 2b** and **2c** absorb around 425 nm, and the ground state bleach signal used was integrated at 500-550 nm, where the triplet states don't absorb). The amount of the triplet state formed at a given time after excitation corresponds to the amount of the ground state which was consumed via intersystem crossing (a ground state bleach signal). The magnitude of the ground state bleach signal (A transient (λ), a negative signal) equals to that of the ground state absorption (A(λ), a positive signal) when the quantum yield of intersystem crossing (ϕ_{ISC}) equals to 1. Their sum equals to 0. If ϕ_{ISC} equals to 0, no ground state bleach is observed. Having this into account, Equation S3 can be derived.

$$A(\lambda) \times \phi_{ISC} = -A^{transient}(\lambda) \text{S3)}$$

The ISC quantum yield can be obtained by the resolution of the integral of Equation S4, the sum of residuals.

$$\int_{\lambda_1}^{\lambda_2} \left[\frac{-A^{transient}(\lambda)}{\phi_{ISC}} - A(\lambda) \right] d\lambda = 0 \text{S4)}$$

Detailed procedure. Steady-state absorption spectra of 10 μM solutions of **2a, 2b** and **2c** in MeCN (black line in Figure 3 of the main text, also green solid line in Figure S2) were measured in a spectrofotometer. The transient spectra of **2a, 2b** and **2c** were measured in the LFP setup, 50 ns

after the laser pulse and 3 averages (red line in Figure 3 of the main text). Then, the ϕ_{ISC} calculated as follows:

1. Multiply the transient spectra ($A^{transient}(\lambda)$) by -1 (blue line in Figure 3 of the main text).
2. Subtract the steady state absorption spectra $-A(\lambda)$ from $-A^{transient}(\lambda)$, which gives $A^{residuals}(\lambda)$.
3. Integrate $A^{residuals}(\lambda)$ to obtain its absolute area in the range where the triplet state does not absorb (500-550 nm). The result is the sum of residuals.
4. Minimize numerically the sum of residuals (Equation S4) by dividing $-A^{transient}(\lambda)$ by a variable between 0 and 1 (green line in Figure 3 of the main text), obtaining the ISC quantum yield (ϕ_{ISC}) at the minimum value (Figure S14).

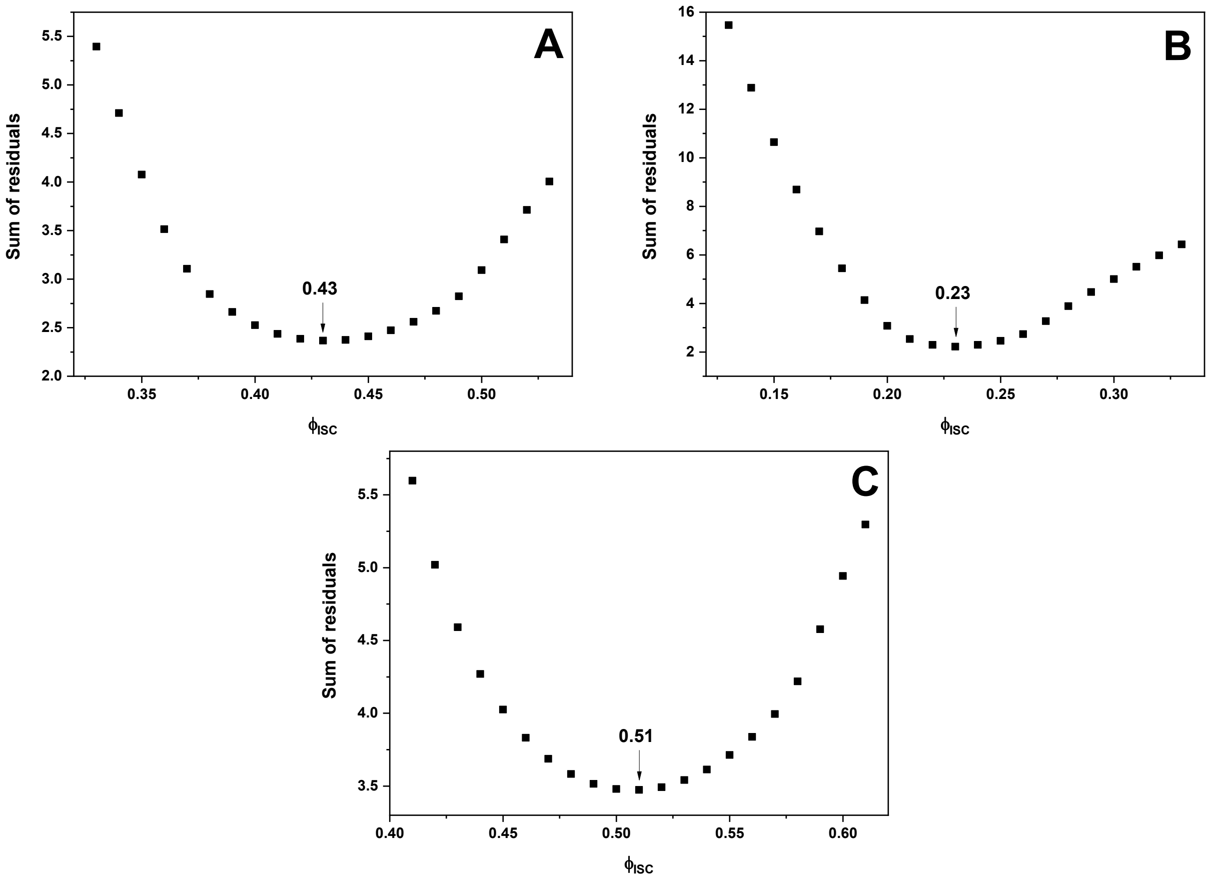

Figure S14. Optimization of the sum of residuals vs ϕ_{ISC} for sensitizers **2a** (**A**), **2b** (**B**) and **2c** (**C**).

The minimum value of the sum of residuals is pointed at, at which ϕ_{ISC} is obtained.

Triplet-triplet energy transfer (TTEnT)

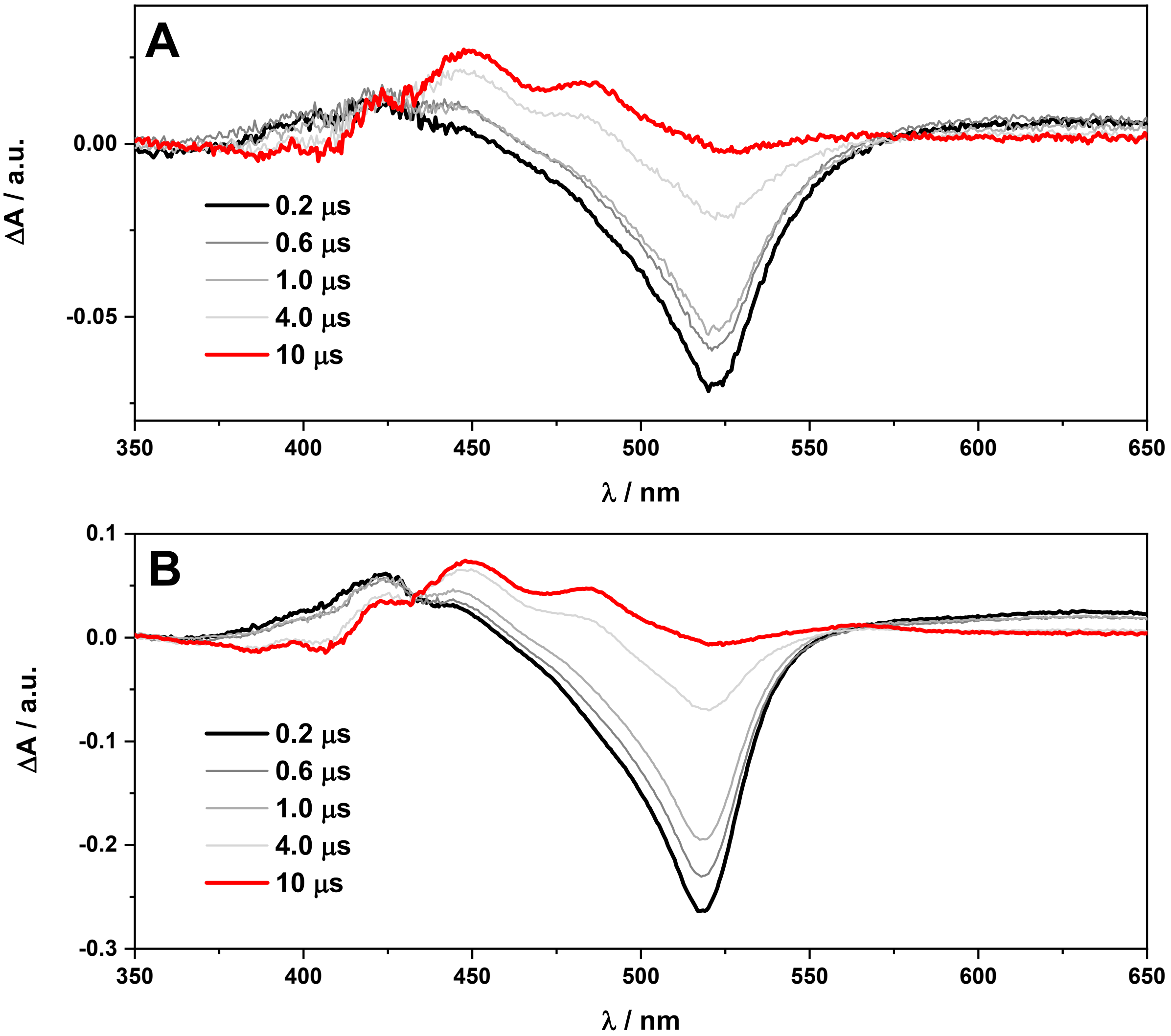

Figure S15. Transient spectra obtained upon LFP (λ_{exc} = 532 nm, 685 mW/cm^2) of **A**: a mixture of **2b** (10 μM) and **TBPe** (100 μM) and **B**: a mixture of **2c** (10 μM) and **TBPe** (100 μM) in degassed MeCN recorded at different times after the laser pulse.

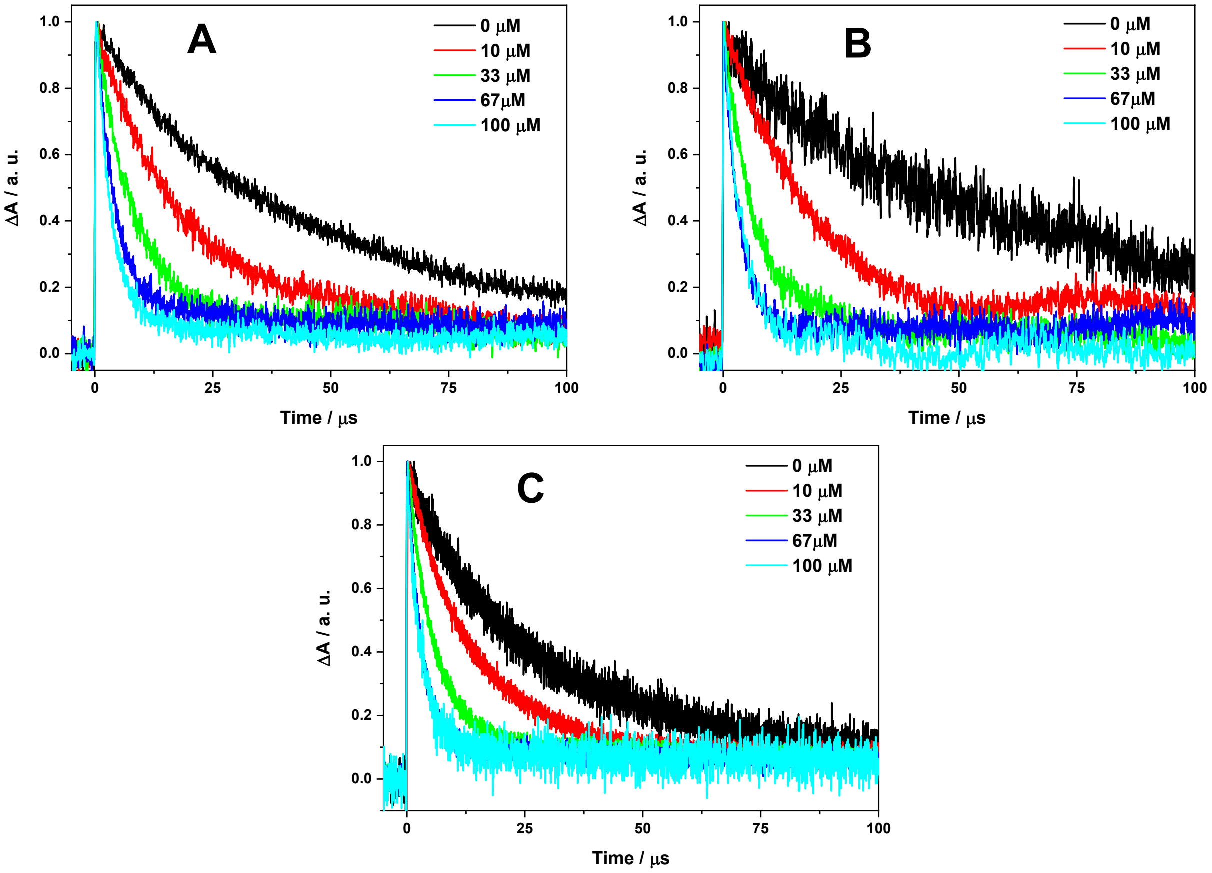

Figure S16. Absorption decay traces at 630 nm of the triplet of 10 μM sensitizers **2a** (**A**), **2b** (**B**) and **2c** (**C**) with increasing concentrations of TBPe in degassed MeCN. Black: 0 μM, red: 10 μM, green: 33 μM, blue: 67 μM, cyan: 100 μM.

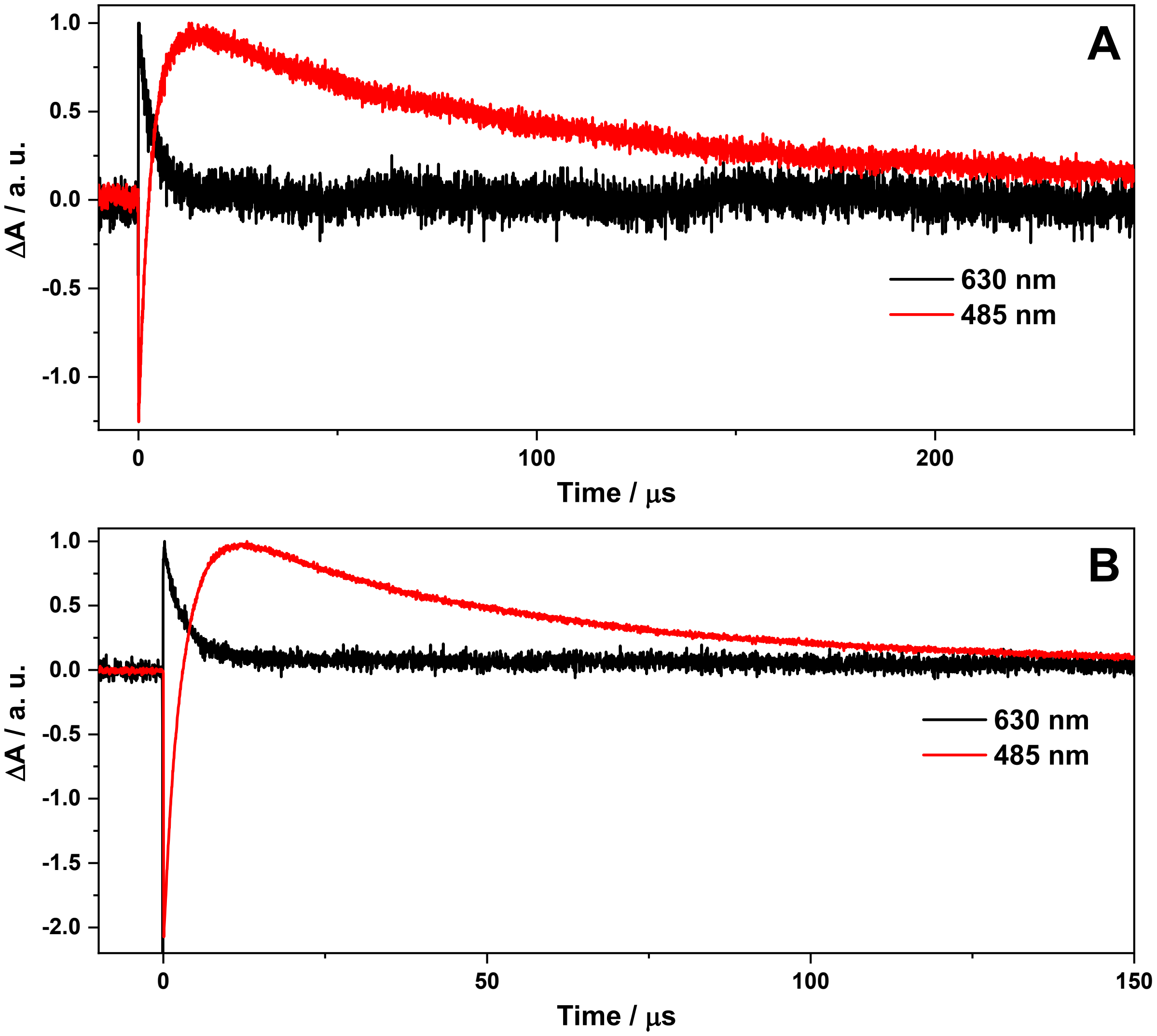

Figure S17. Decay kinetics monitored at 630 nm (black) and 485 nm (red) of 10 μM **2b** (**A**) and **2c** (**B**) in the presence of 100 μM **TBPe** in degassed MeCN after 532 nm LFP.

Triplet-triplet annihilation (TTA)

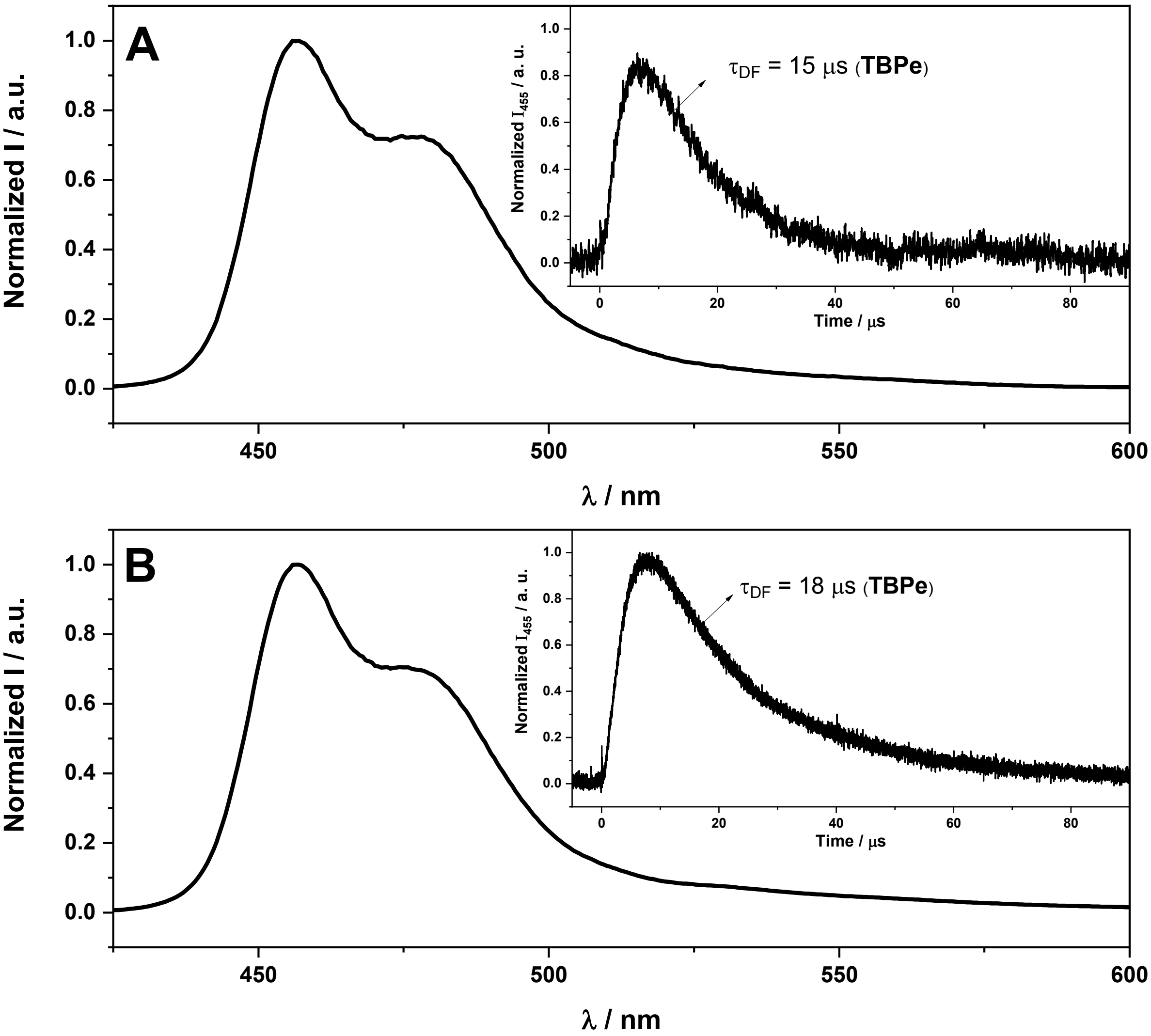

Figure S18. Emission spectrum (λ_{exc} = 532 nm) of a mixture of 10 μM **2b** (**A**) and **2c** (**B**) with 100 μM **TBPe** in N_2/MeCN recorded at 1 μs after the laser pulse. Inset: Kinetic decay of the delayed ^{1}**TBPe*** at 455 nm.

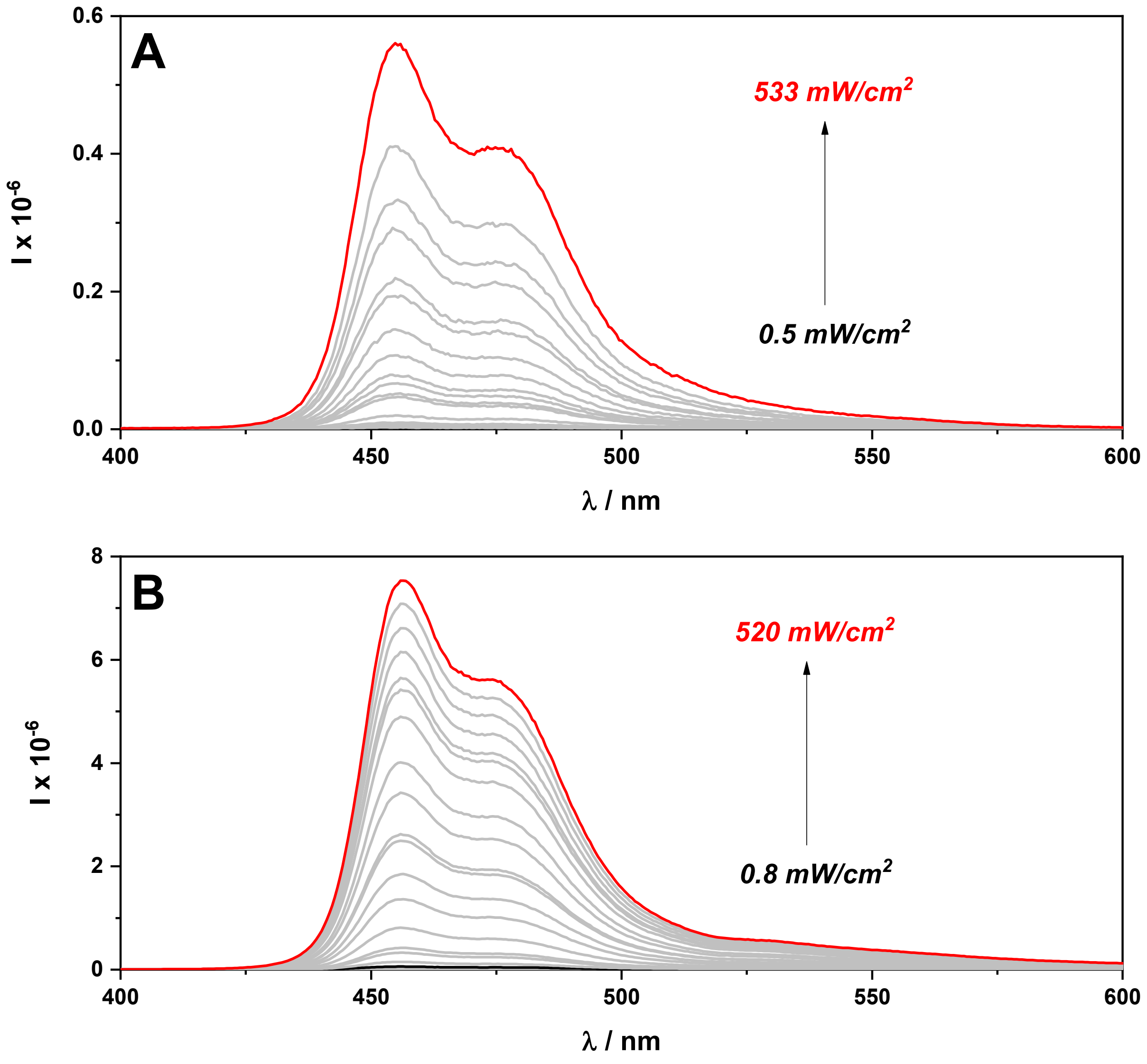

Figure S19. Emission spectra (λ_{exc} = 532 nm) of a mixture of 10 µM **2b** (**A**) and 10 µM **2c** (**B**) with 100 µM **TBPe** (100 µM) in N_2/MeCN recorded at 1 µs after the laser pulse with increasing laser intensity.

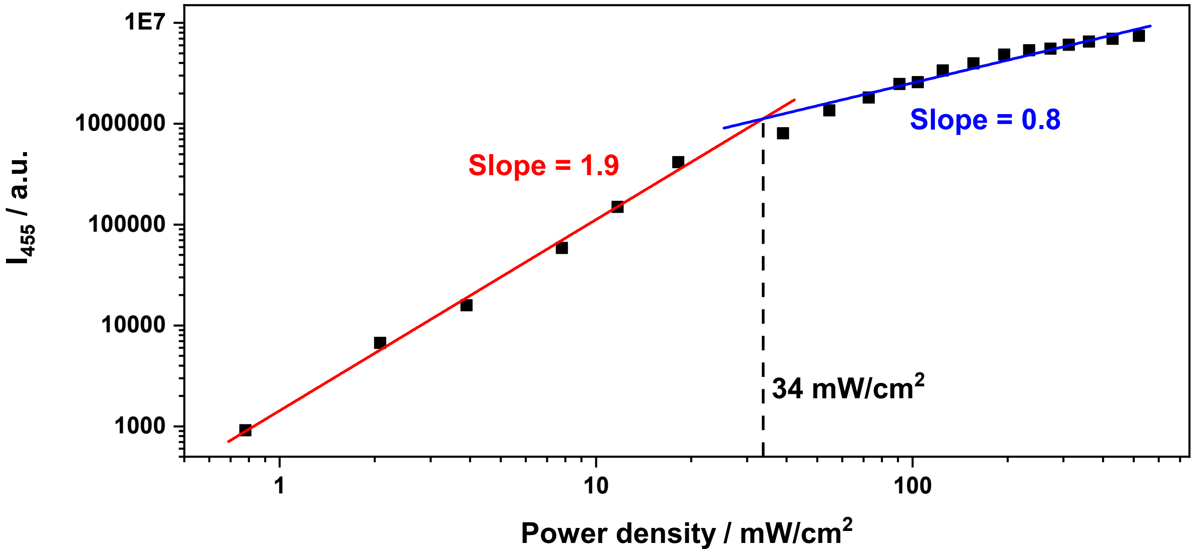

Figure S20. A double logarithmic plot of the delayed fluorescence intensity as a function of the excitation power for the case of **2c/TBPe** pair.

4- ^{1}H, ^{13}C, ^{11}B and ^{19}F NMR spectra

^{1}H NMR spectra of **3a**

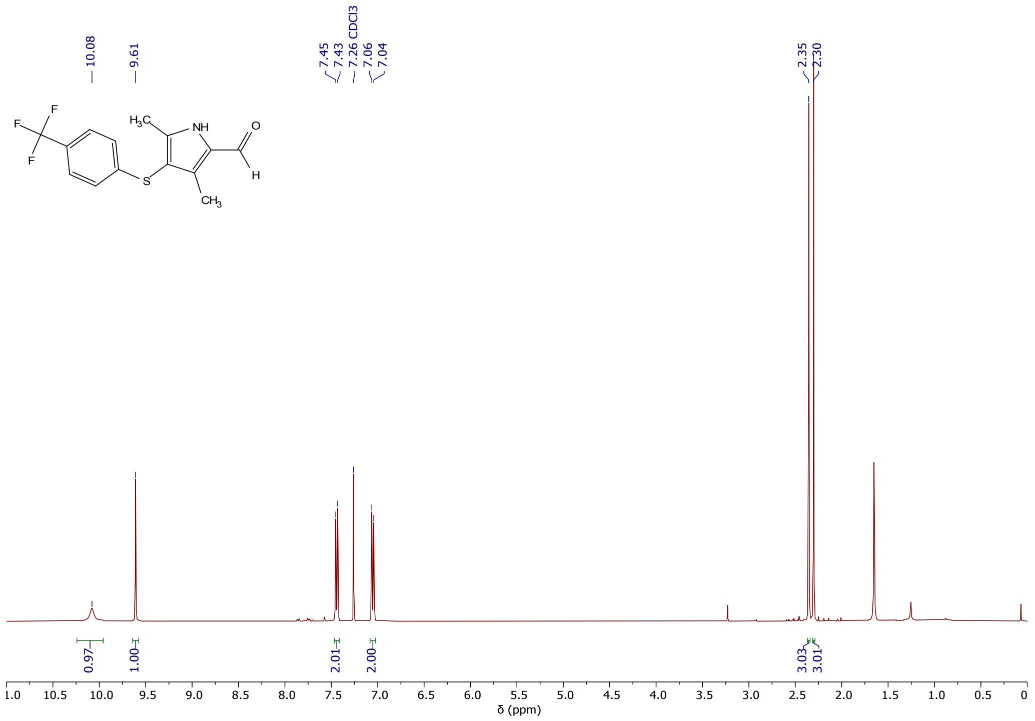

^{13}C NMR spectra of **3a**

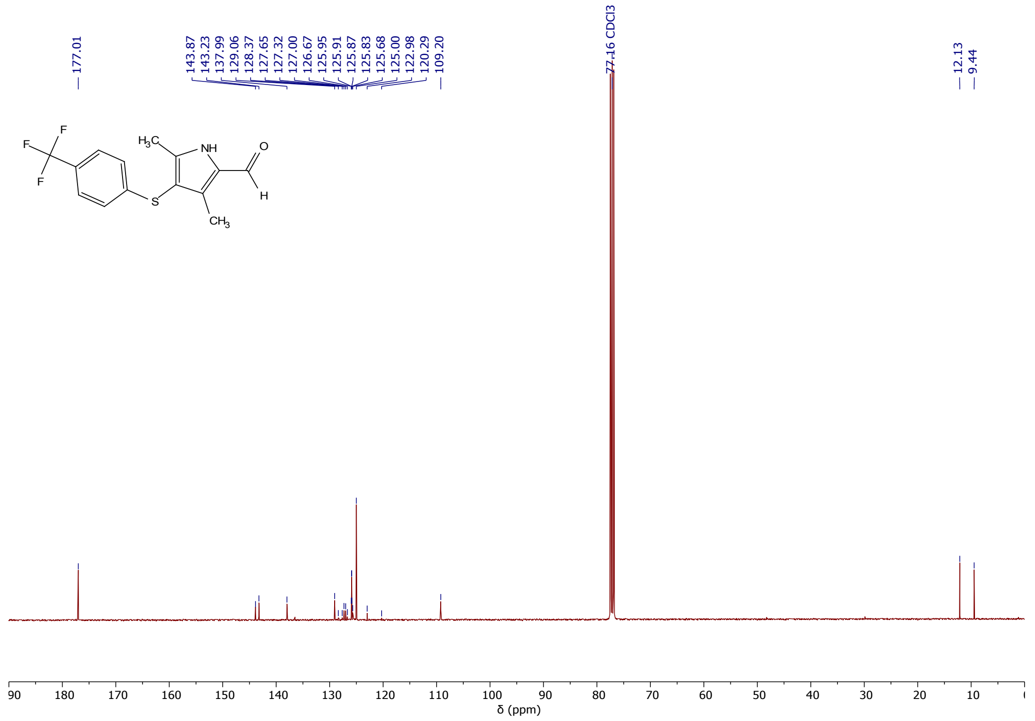

^{19}F NMR spectra of **3a**

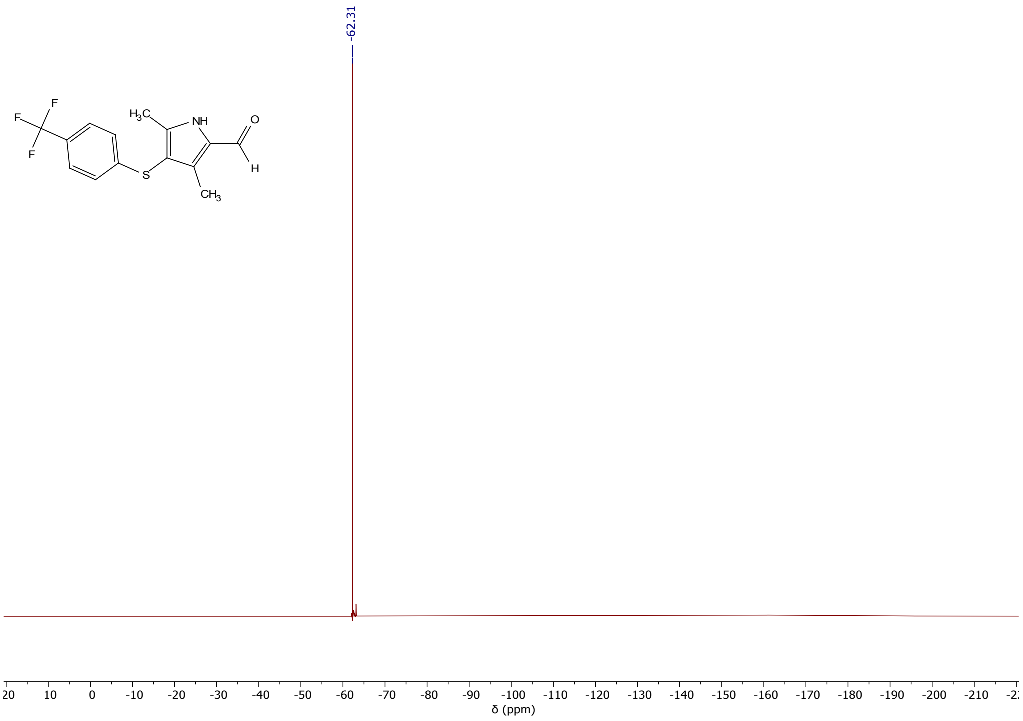

^{1}H NMR spectra of **3b**

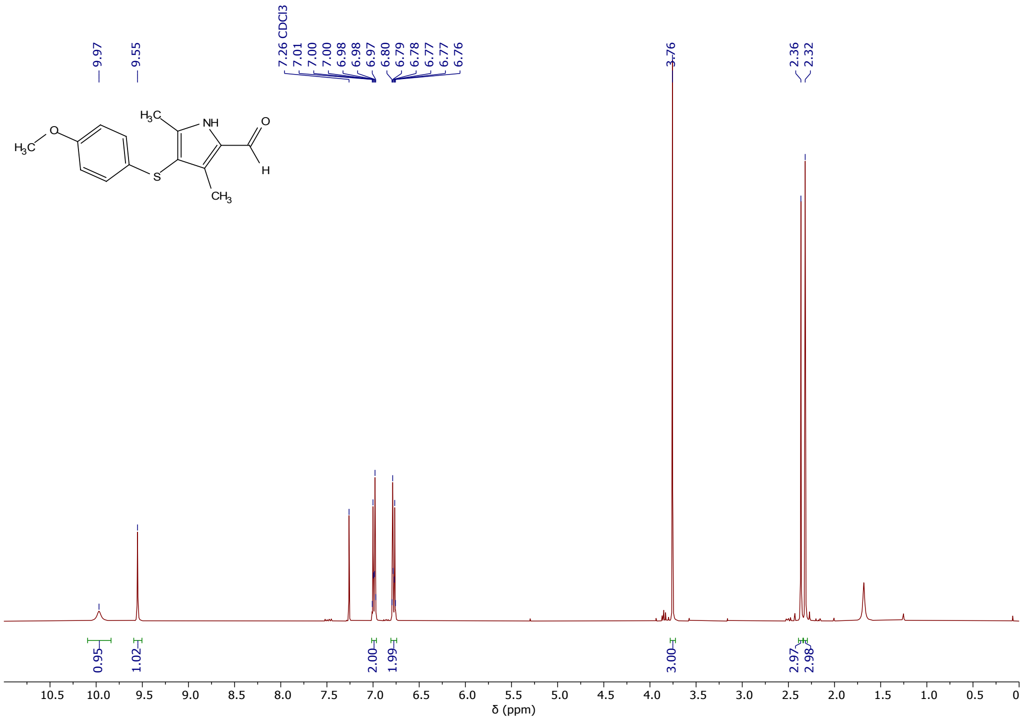

^{13}C NMR spectra of **3b**

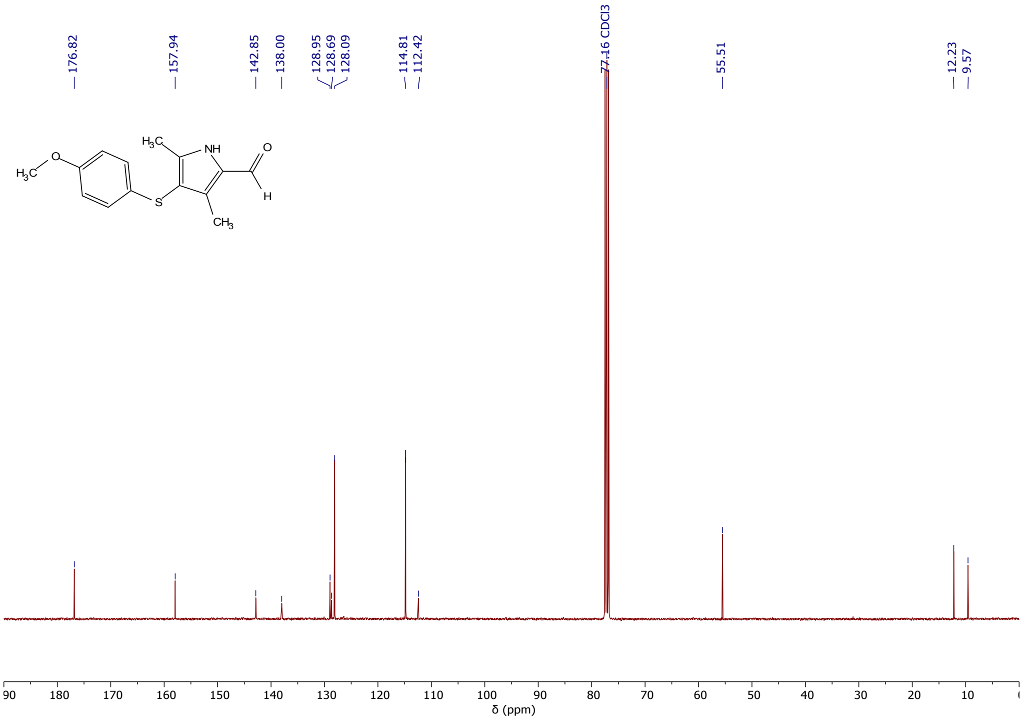

^{1}H NMR spectra of **3c**

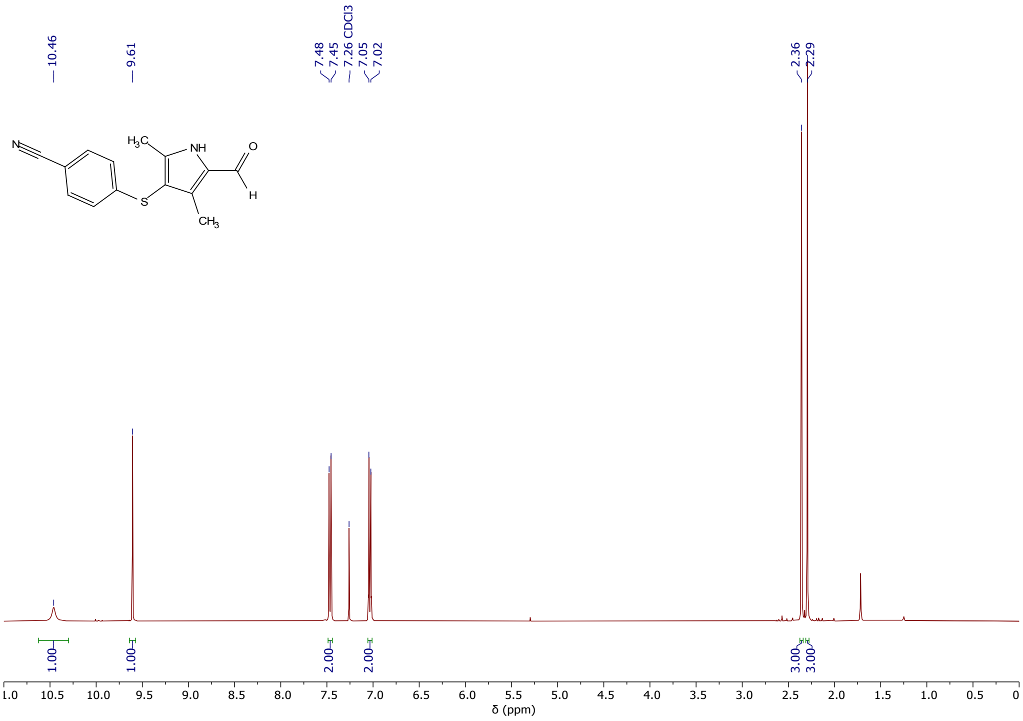

^{13}C NMR spectra of **3c**

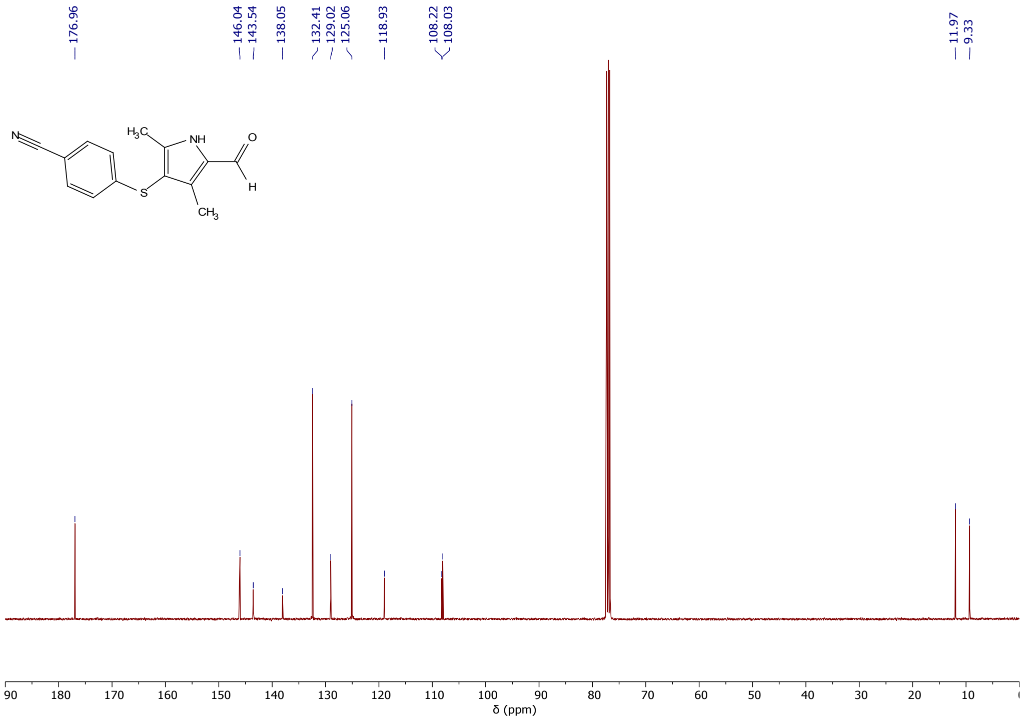

^{1}H NMR spectra of **4a**

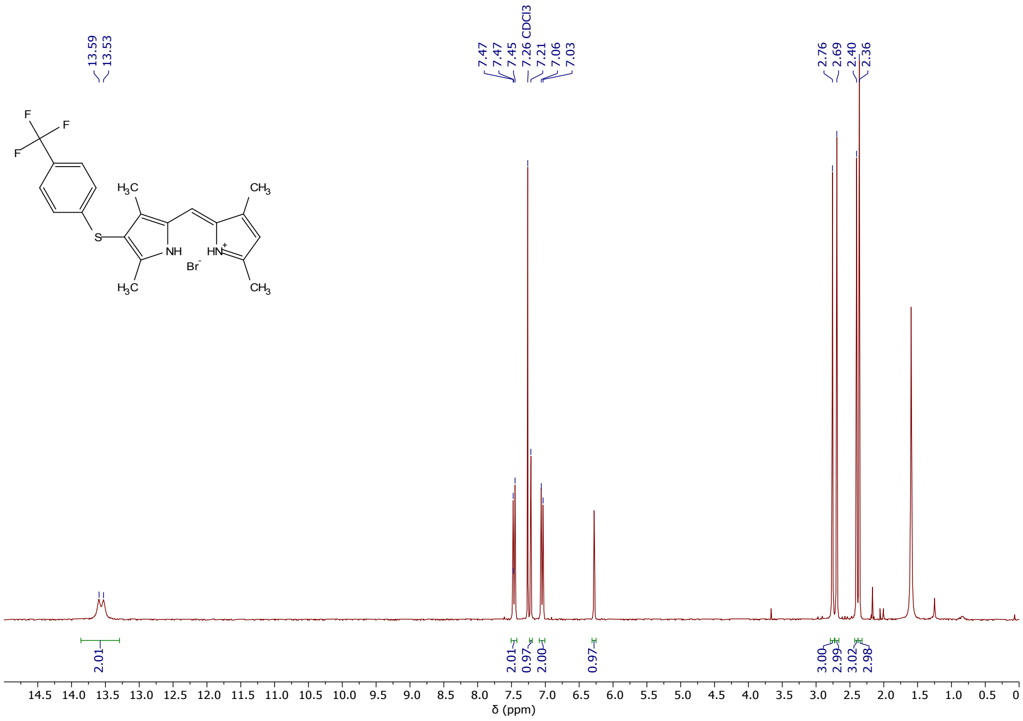

^{13}C NMR spectra of **4a**

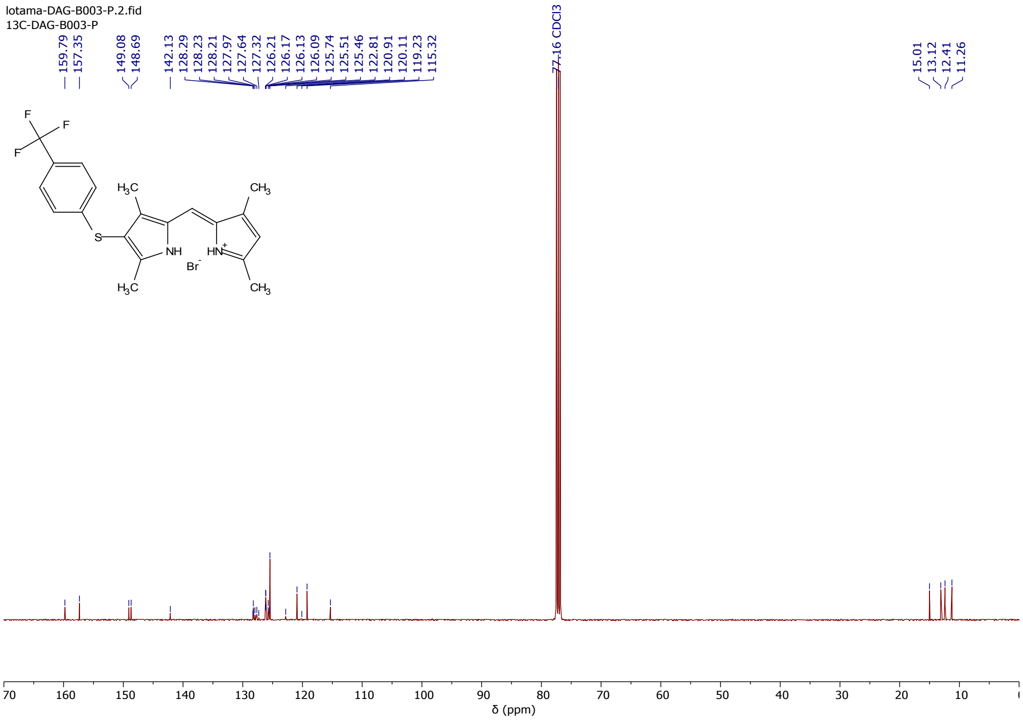

^{19}F NMR spectra of **4a**

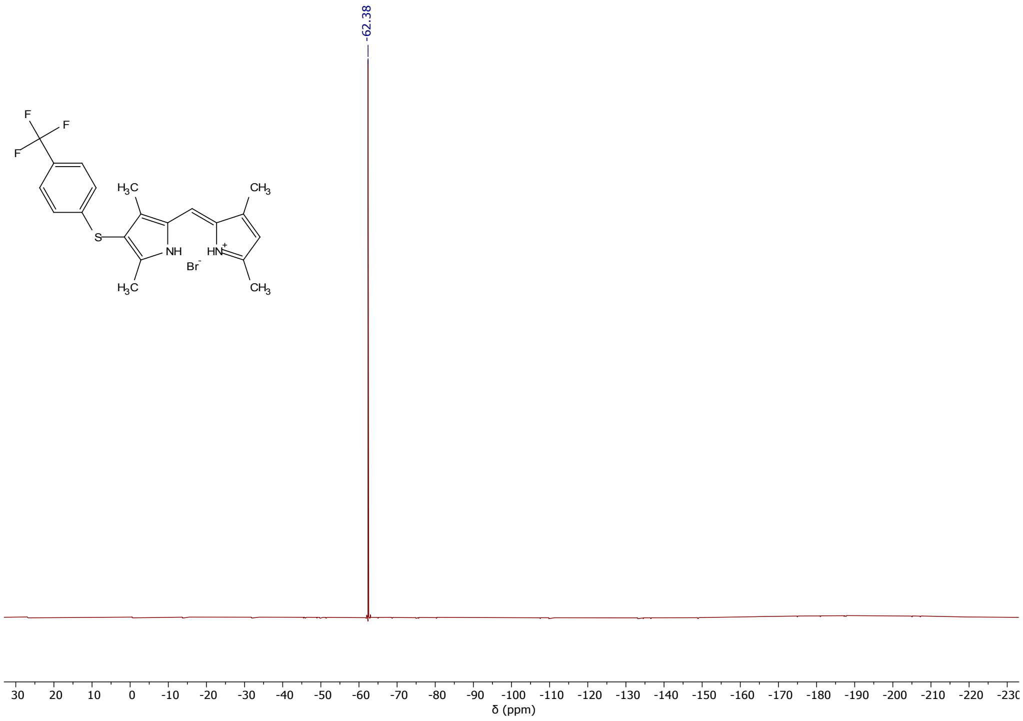

^{1}H NMR spectra of **4b**

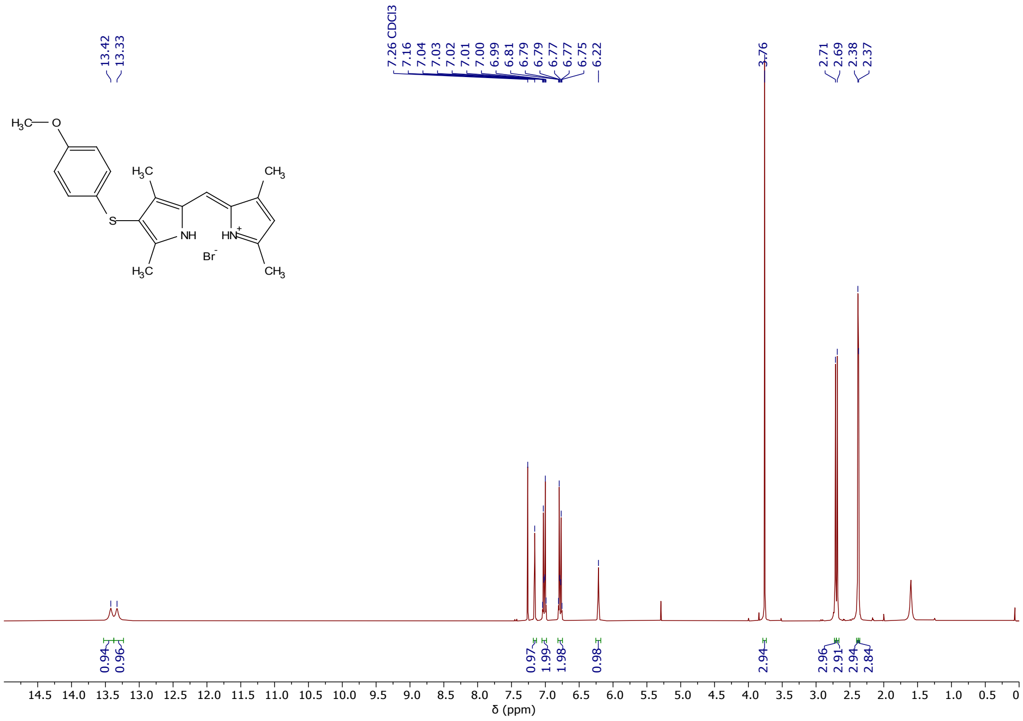

^{13}C NMR spectra of **4b**

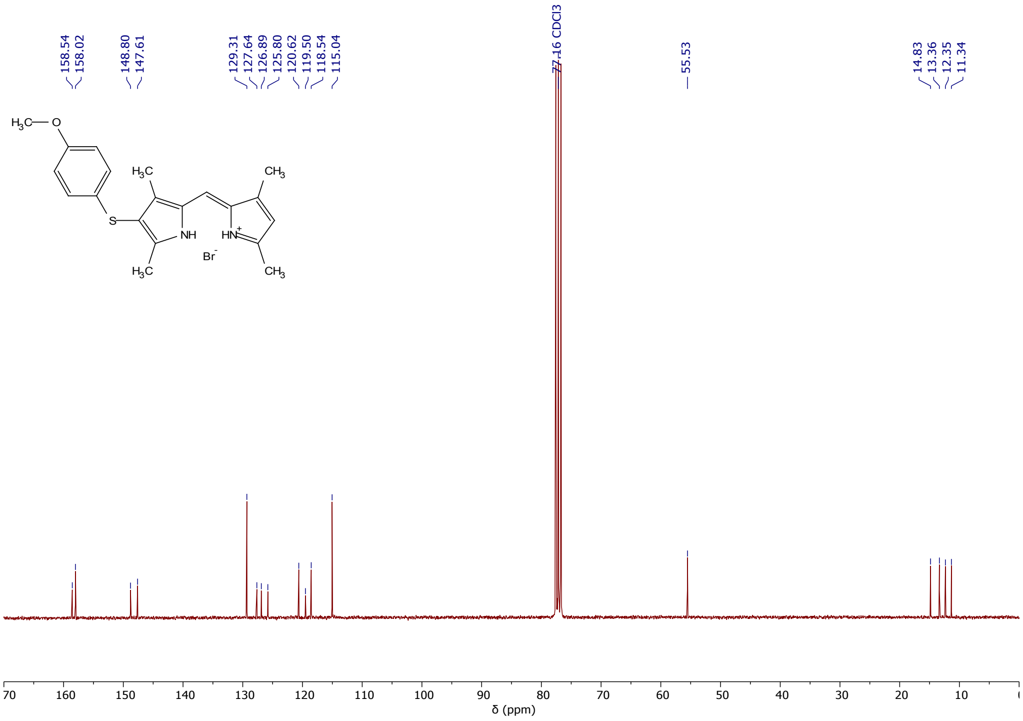

^{1}HNMR spectra of **4c**

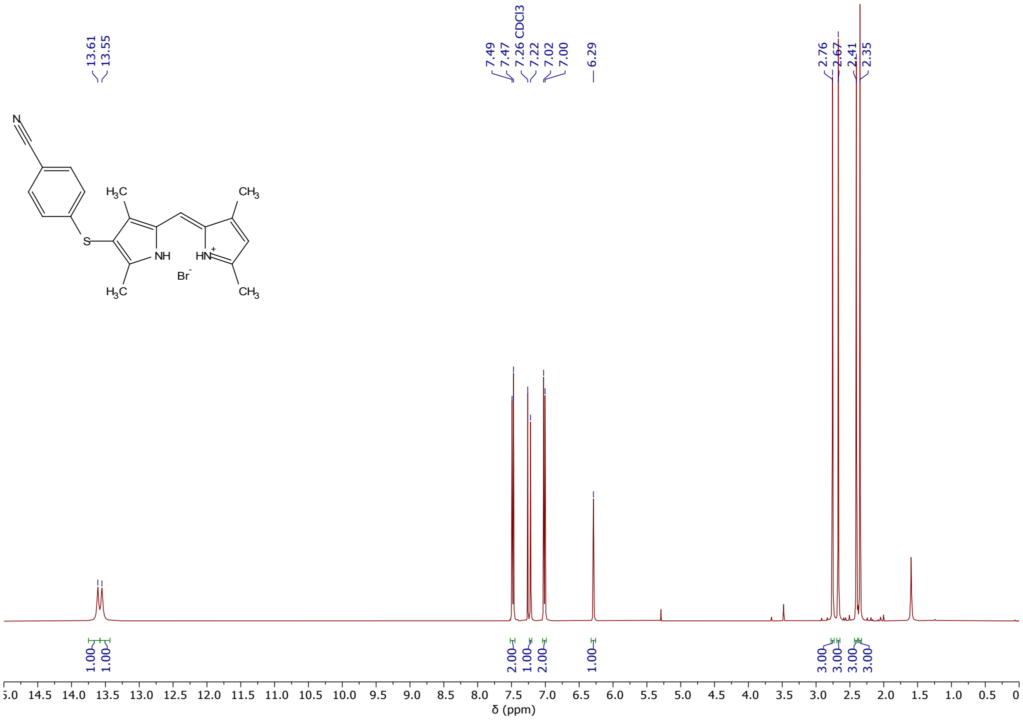

^{13}C NMR spectra of **4c**

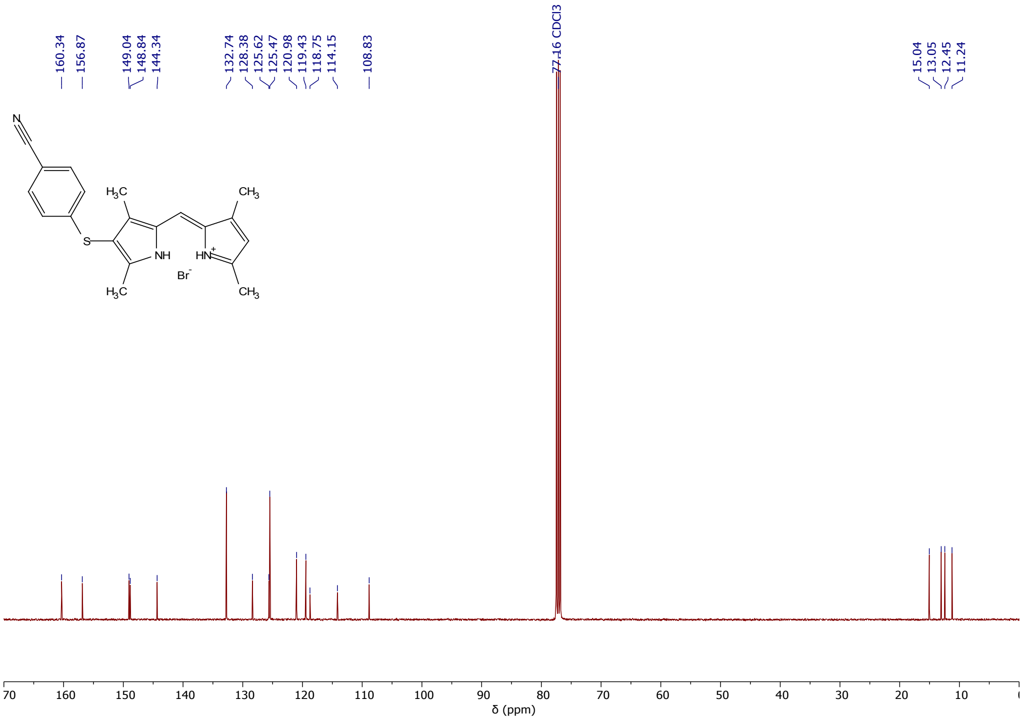

^{1}H NMR spectra of **1a**

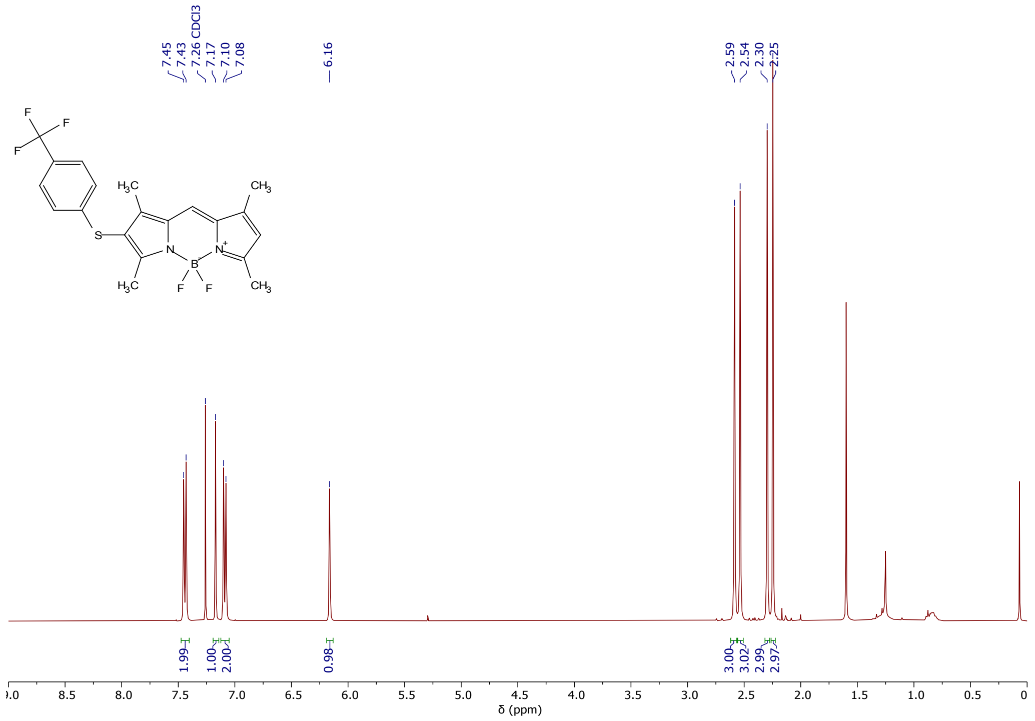

^{13}C NMR spectra of **1a**

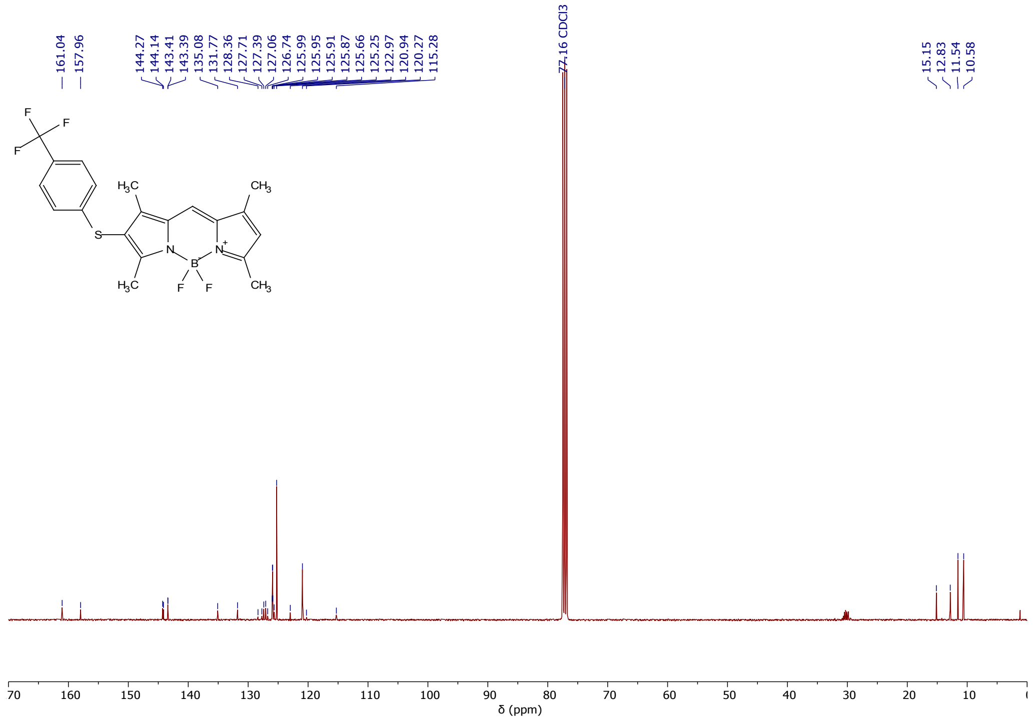

^{11}B NMR spectra of **1a**

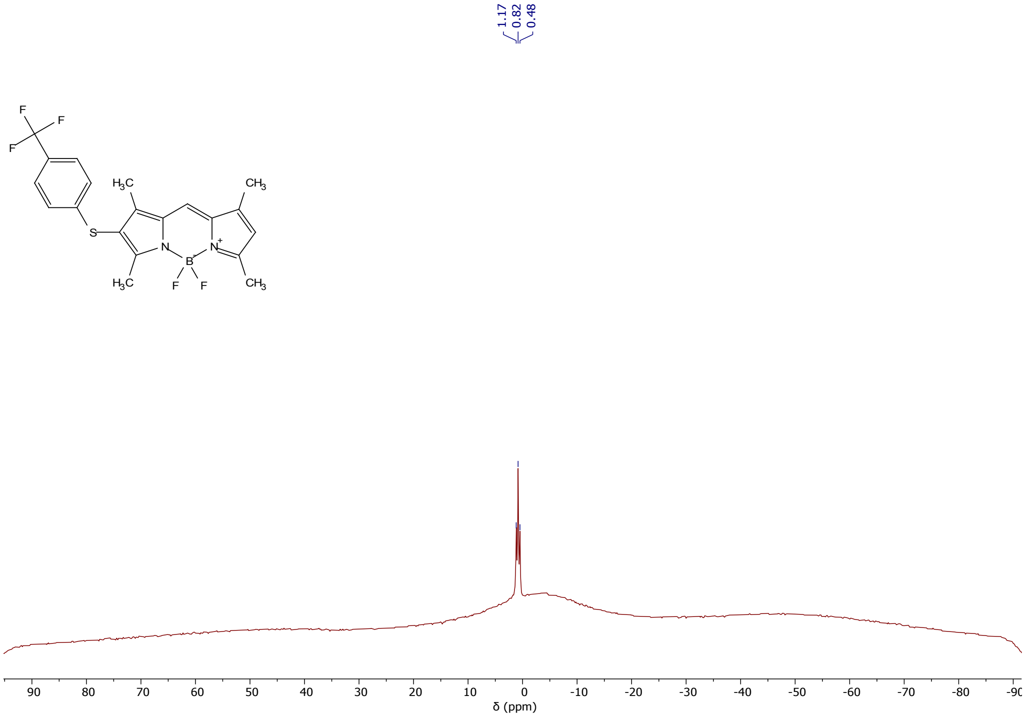

^{19}F NMR spectra of **1a**

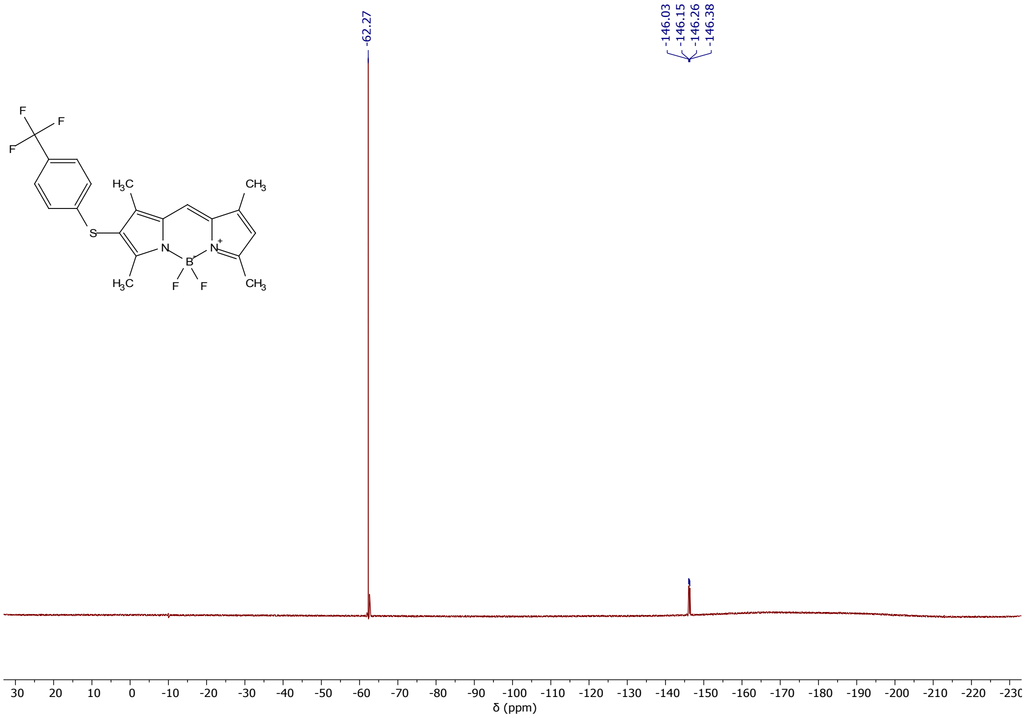

^{1}H NMR spectra of **1b**

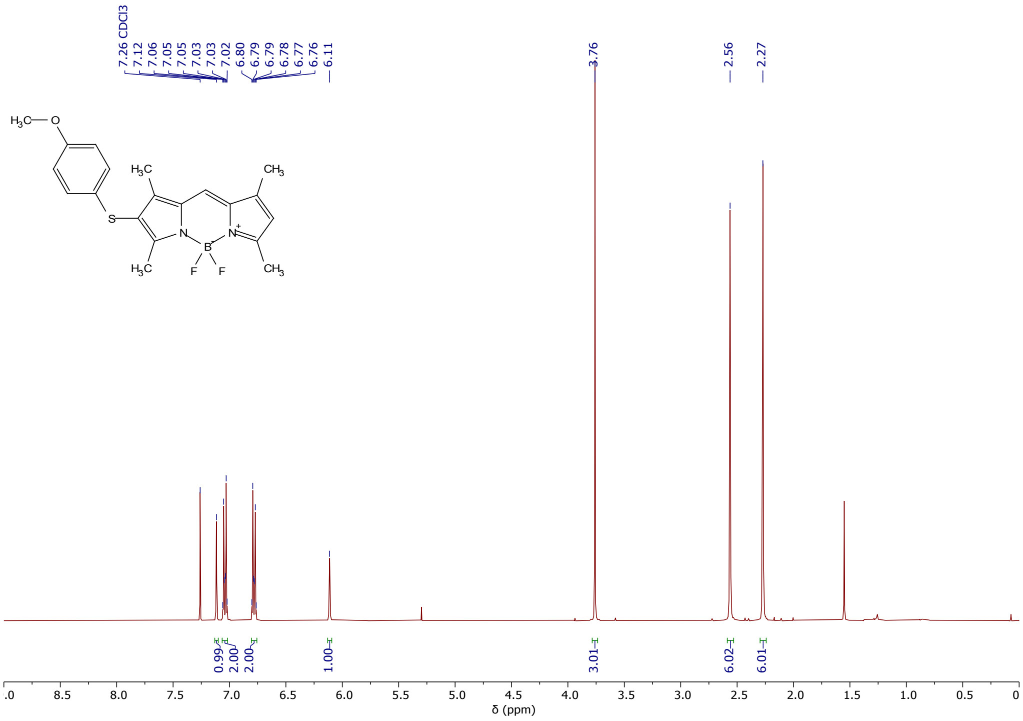

^{13}C NMR spectra of **1b**

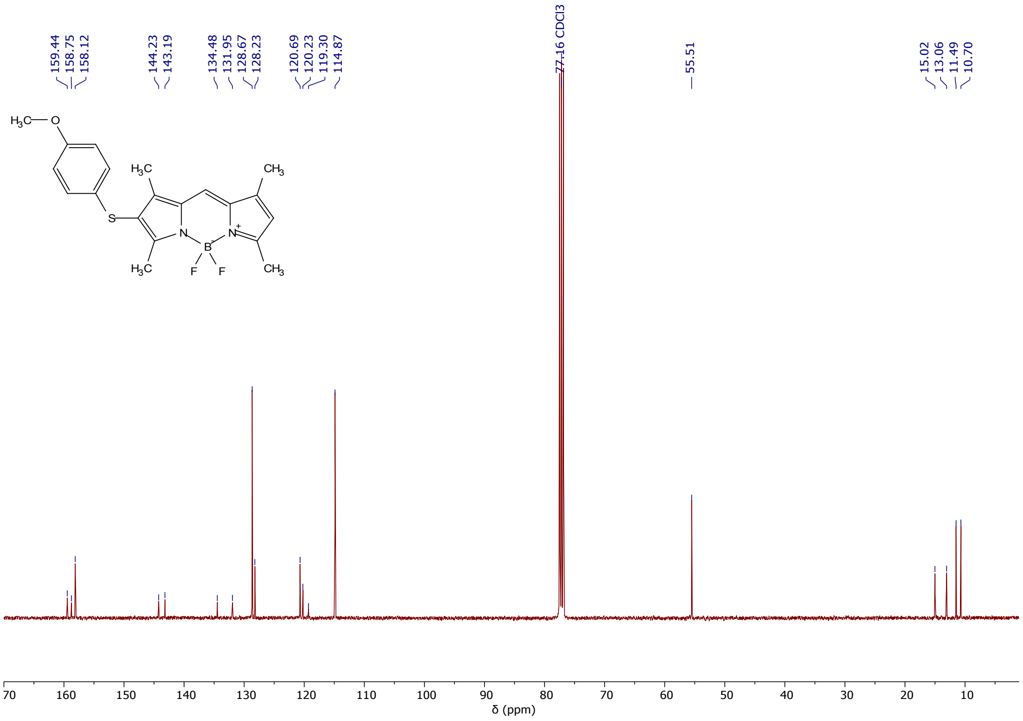

^{11}B NMR spectra of **1b**

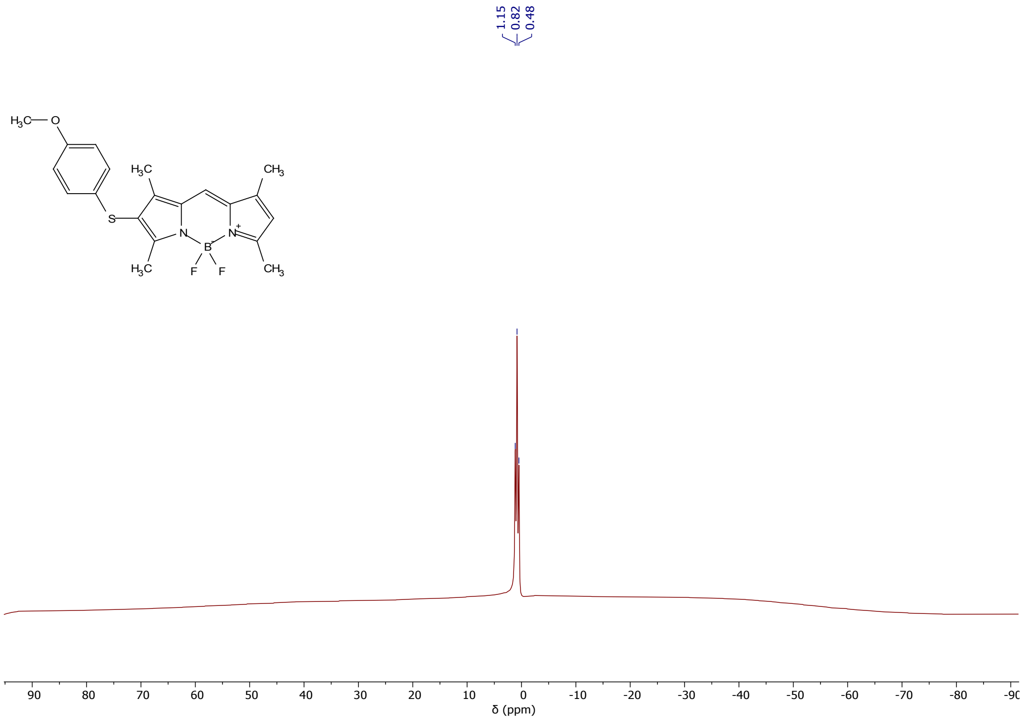

^{19}F NMR spectra of **1b**

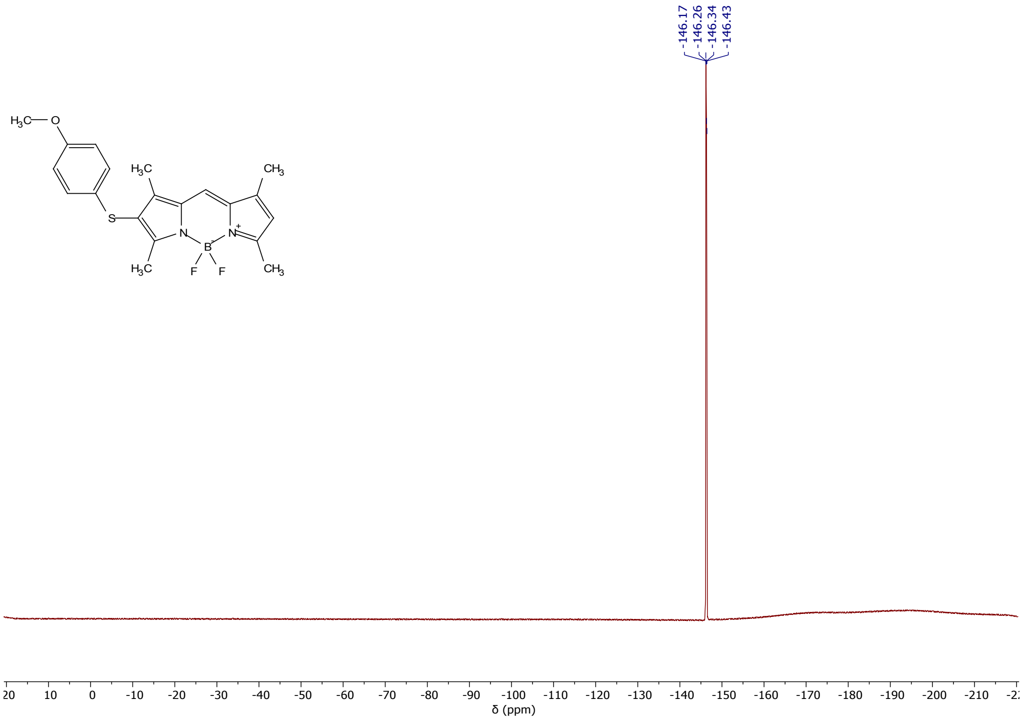

^{1}H NMR spectra of **1c**

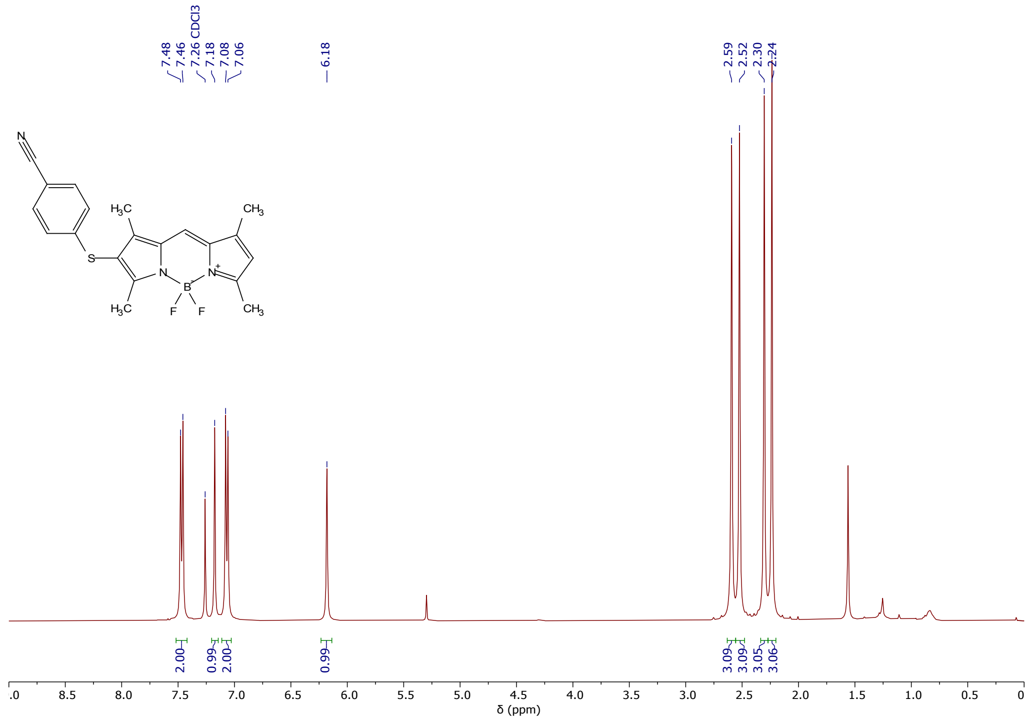

^{13}C NMR spectra of **1c**

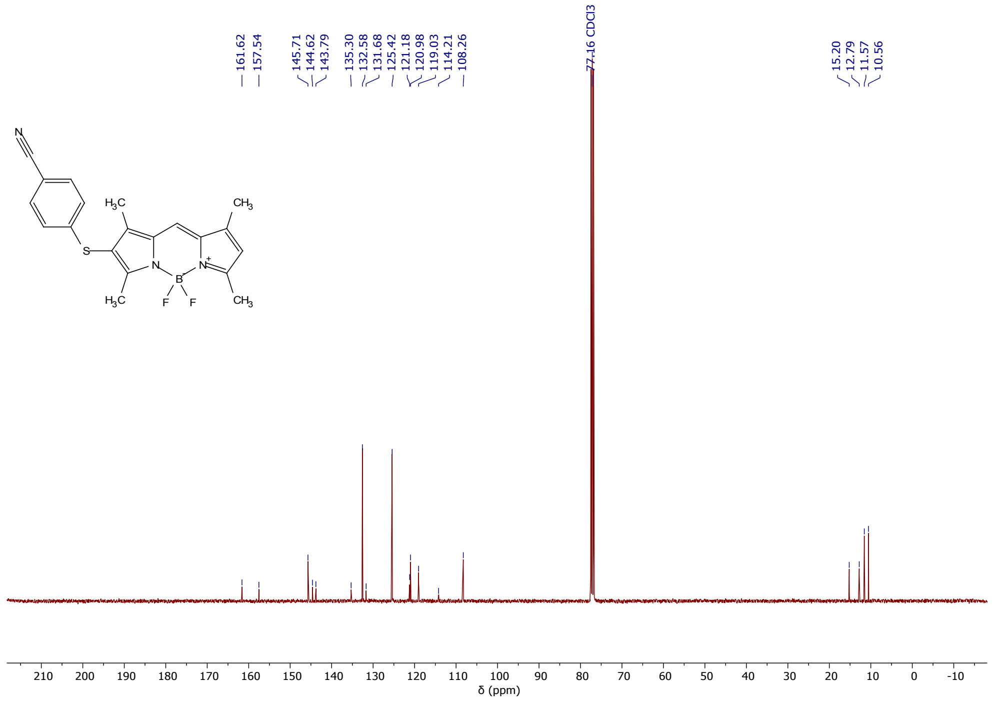

^{11}B NMR spectra of **1c**

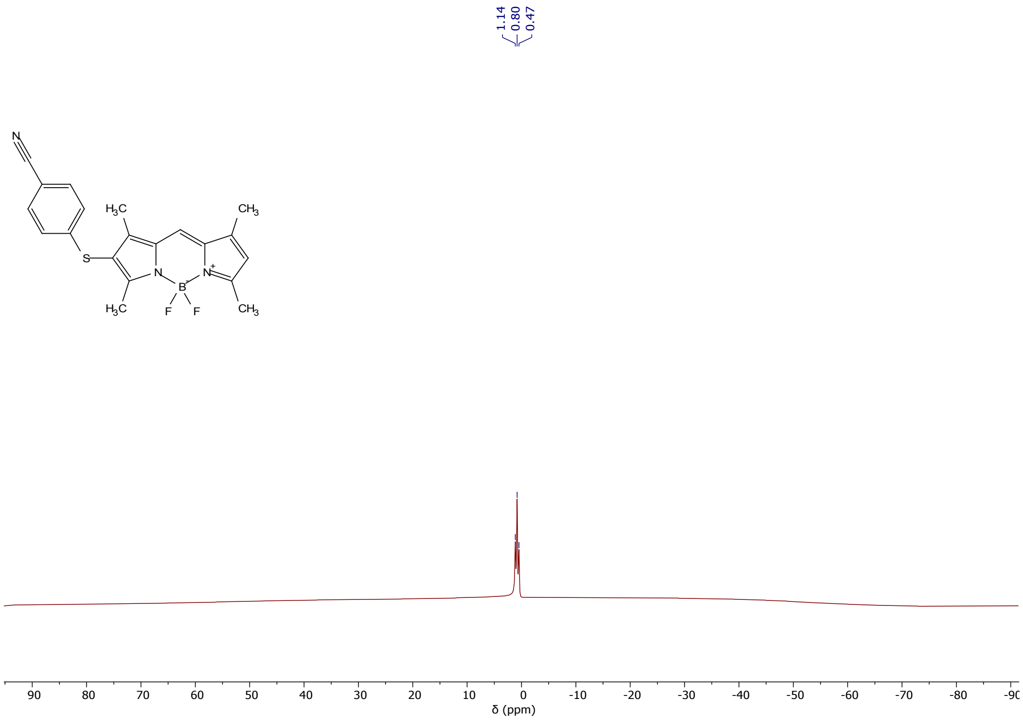

^{19}F NMR spectra of **1c**

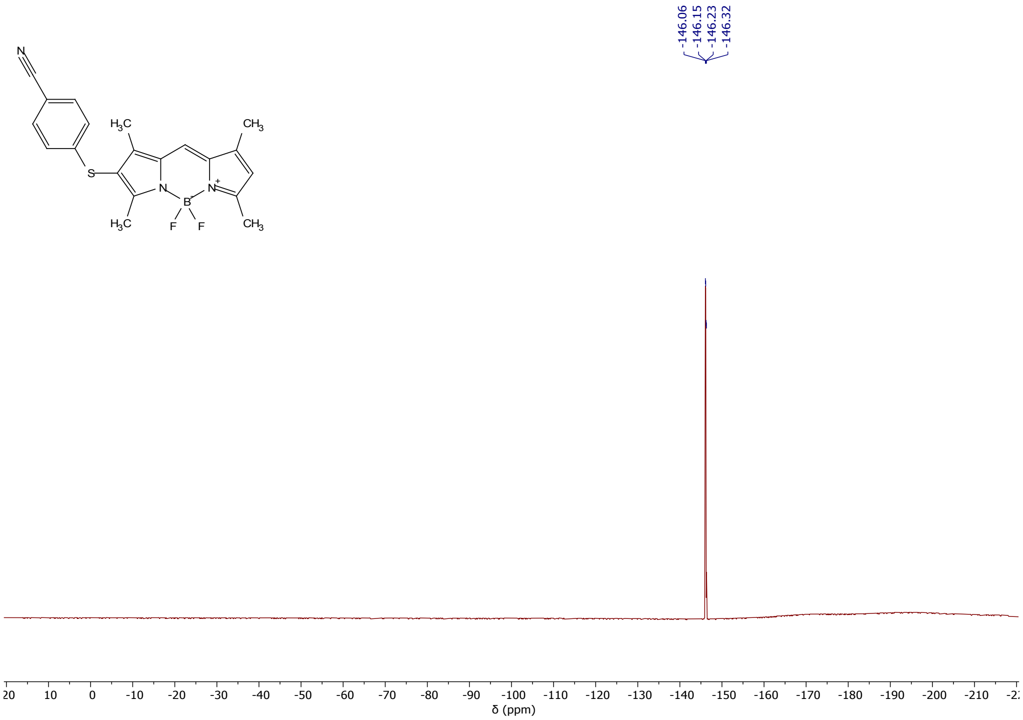

^{1}H NMR spectra of **2a**

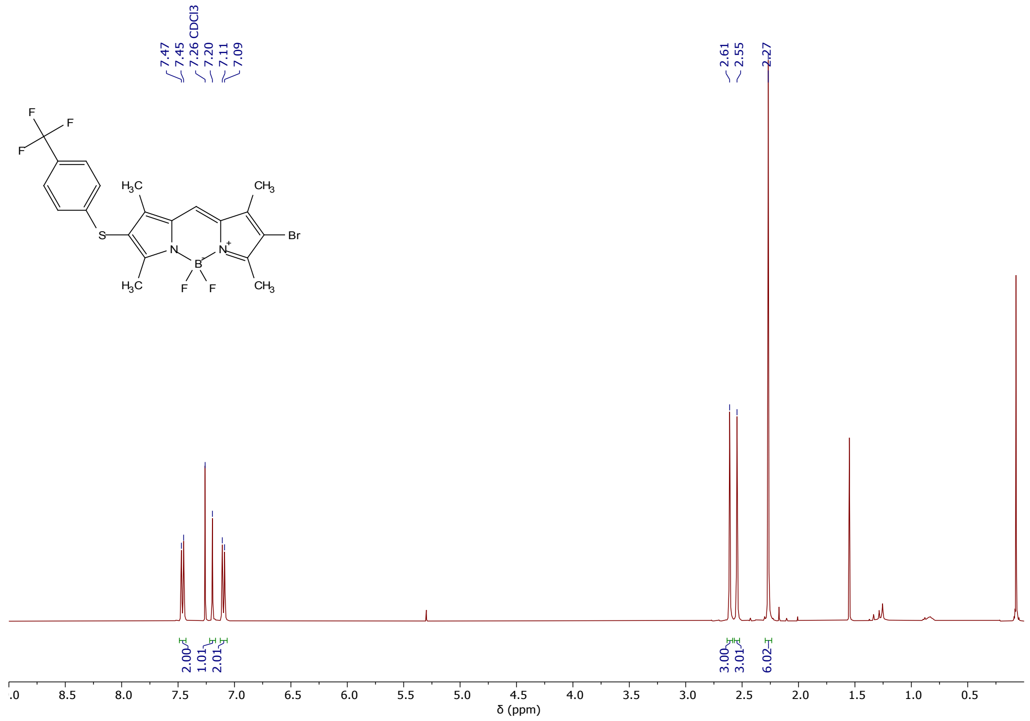

^{13}C NMR spectra of **2a**

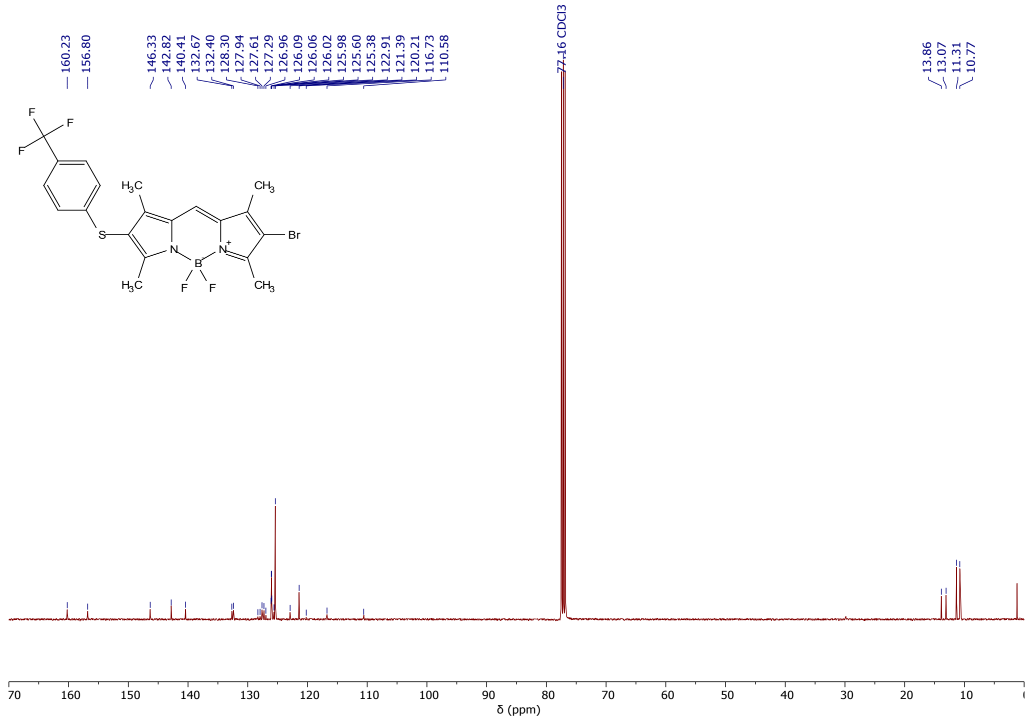

^{11}B NMR spectra of **2a**

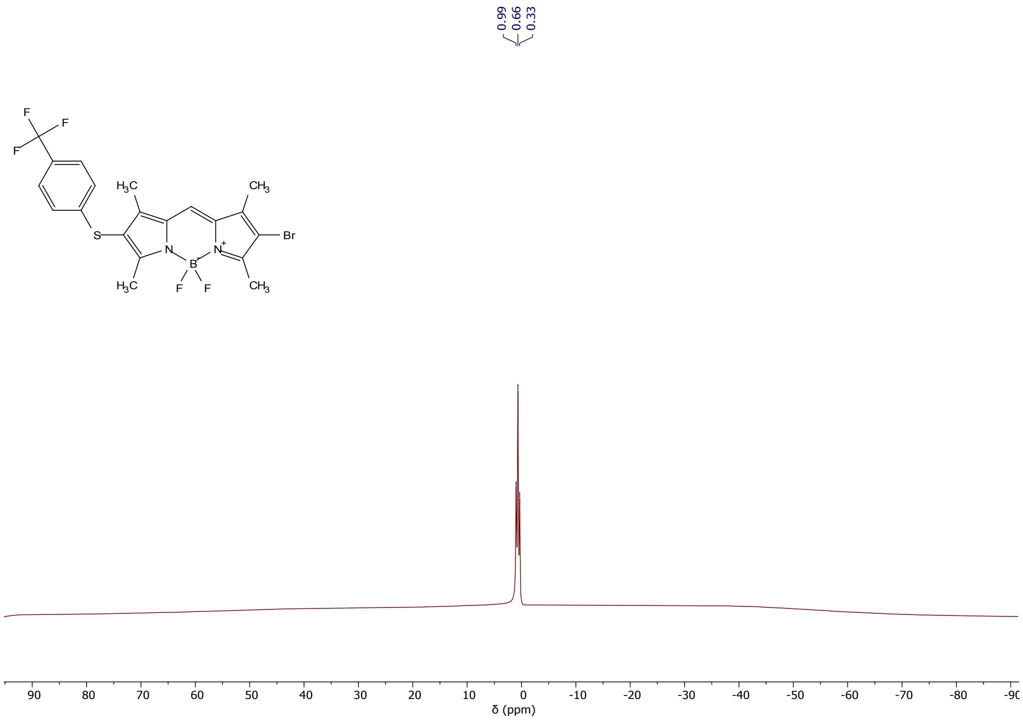

^{19}F NMR spectra of **2a**

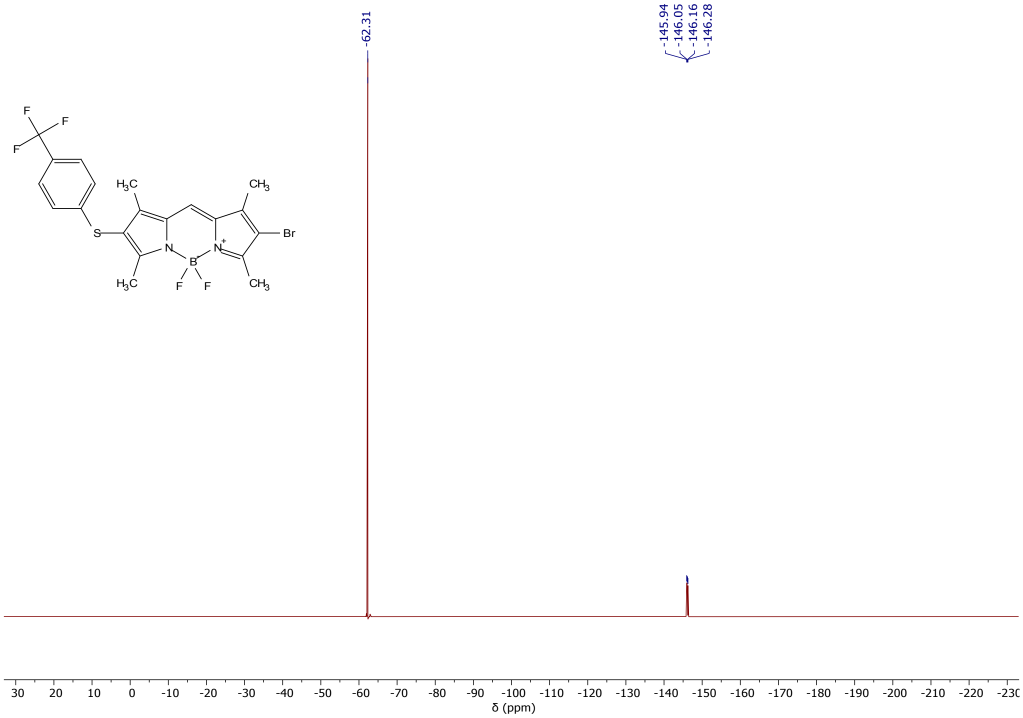

^{1}H NMR spectra of **2b**

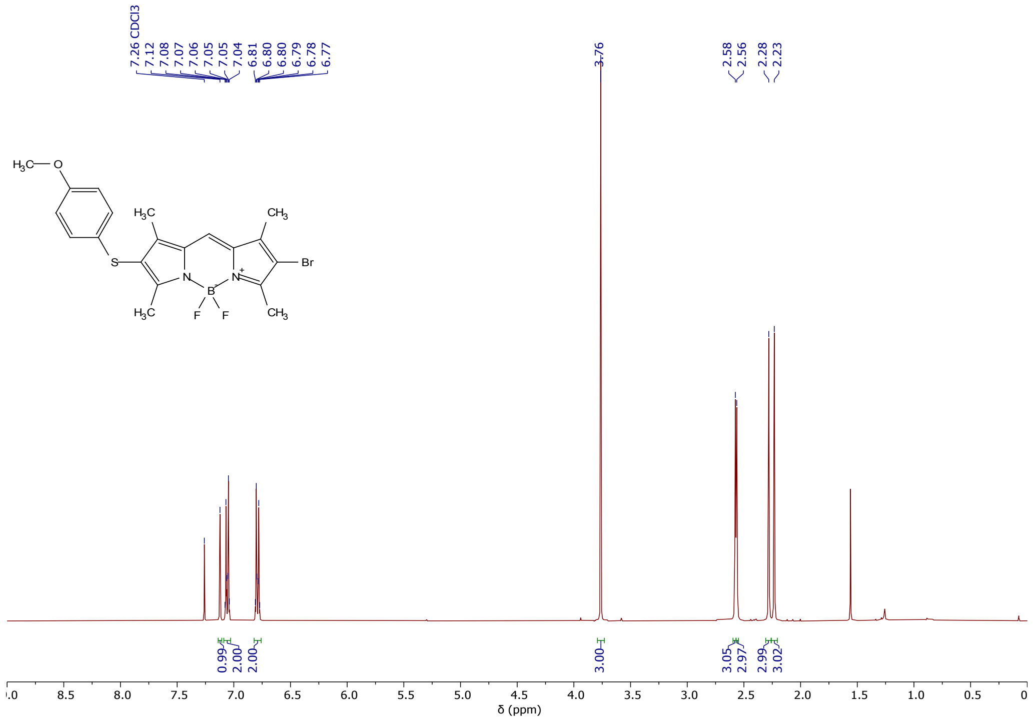

^{13}C NMR spectra of **2b**

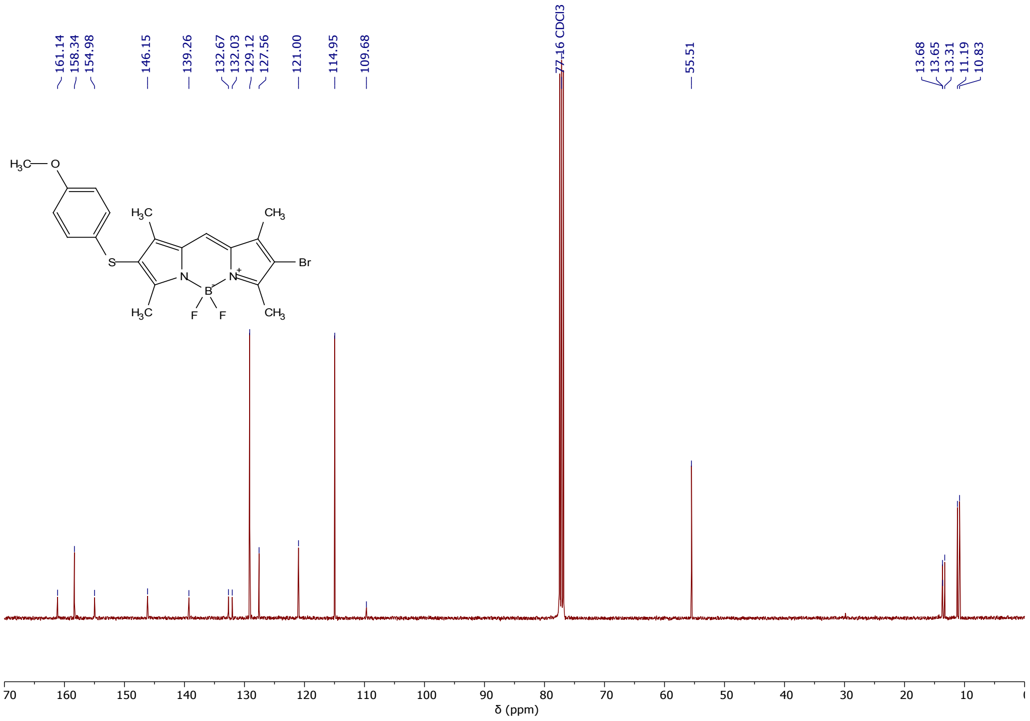

^{11}B NMR spectra of **2b**

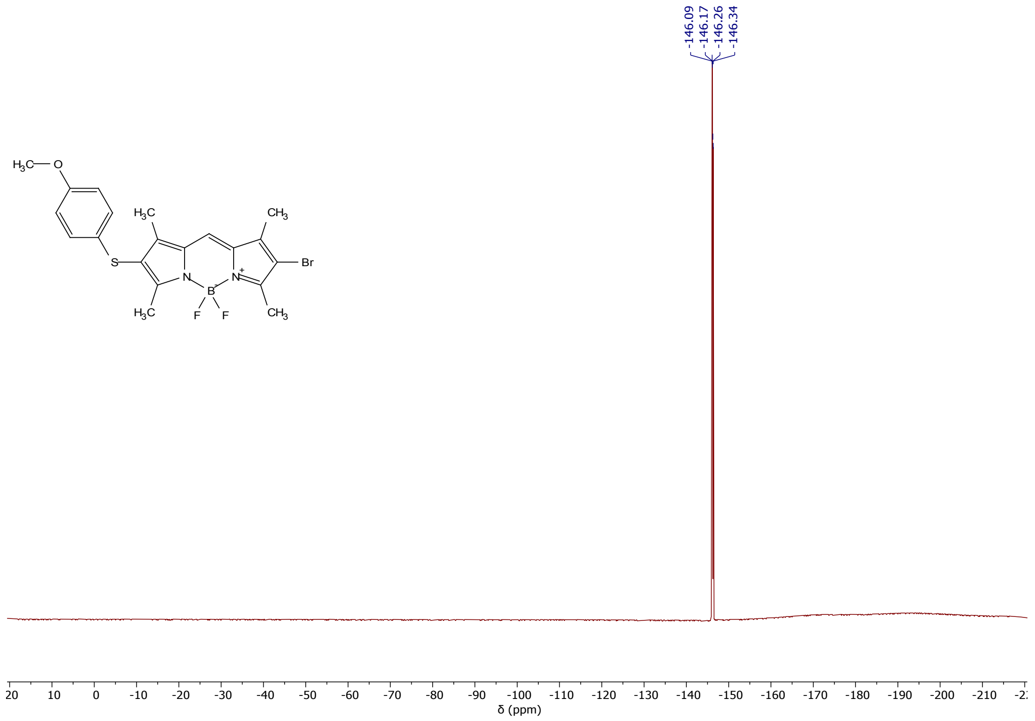

^{19}F NMR spectra of **2b**

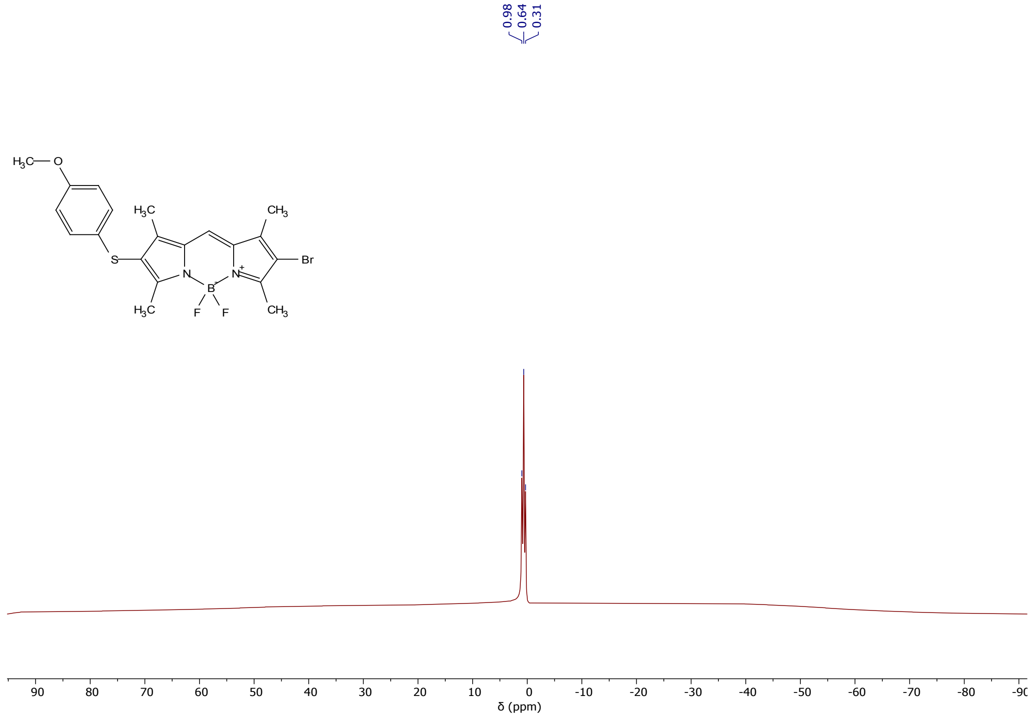

^{1}H NMR spectra of **2c**

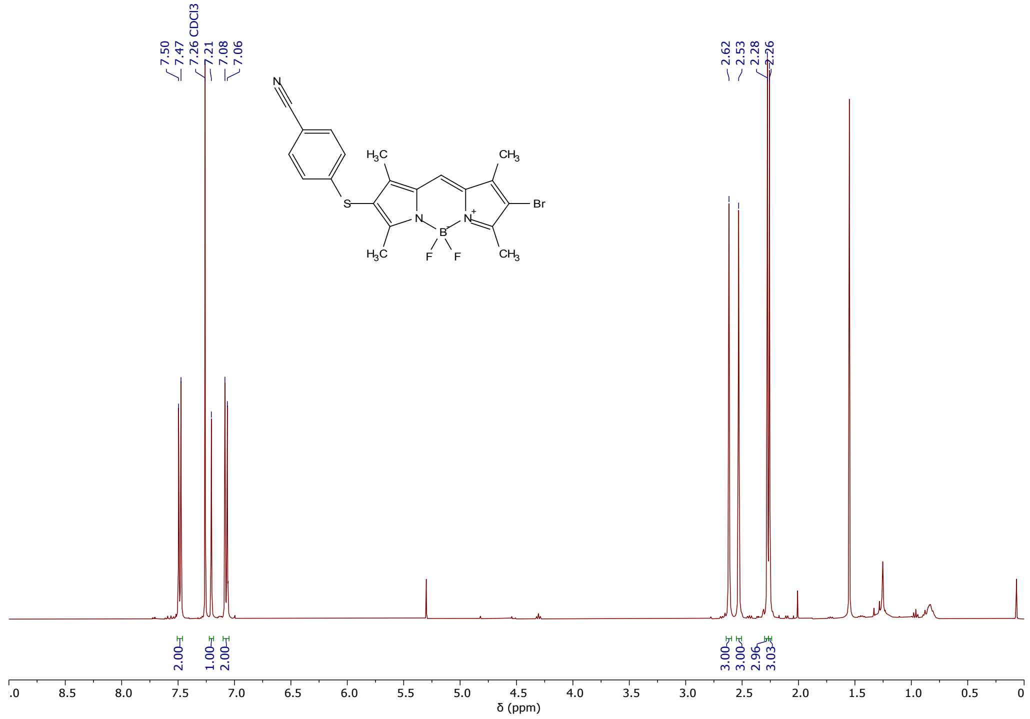

^{13}C NMR spectra of **2c**

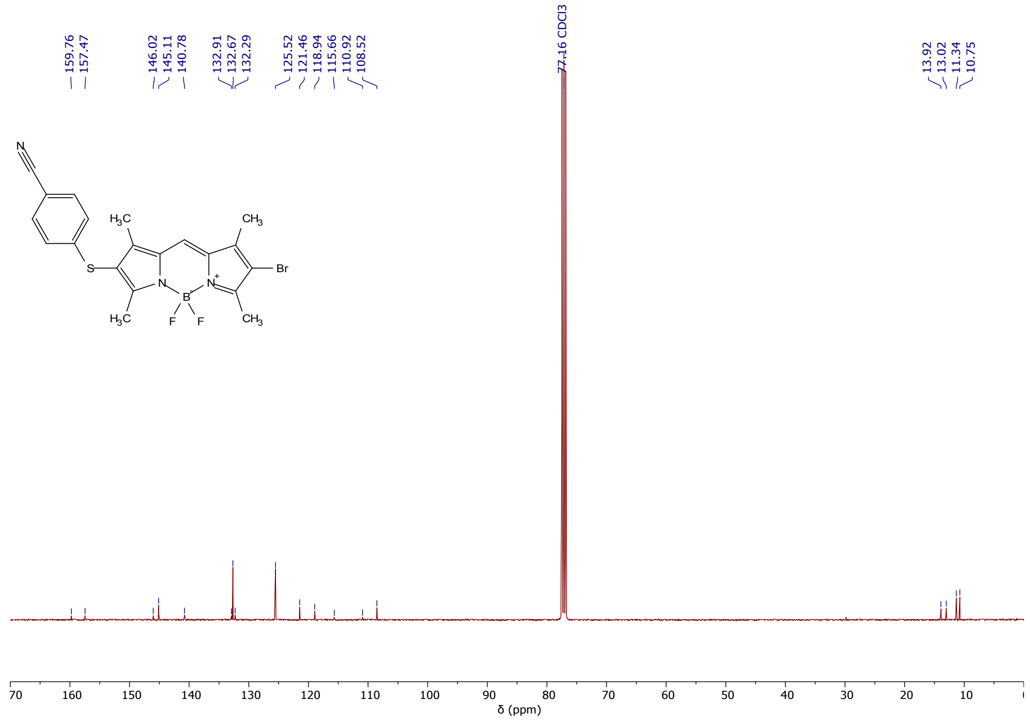

^{11}B NMR spectra of **2c**

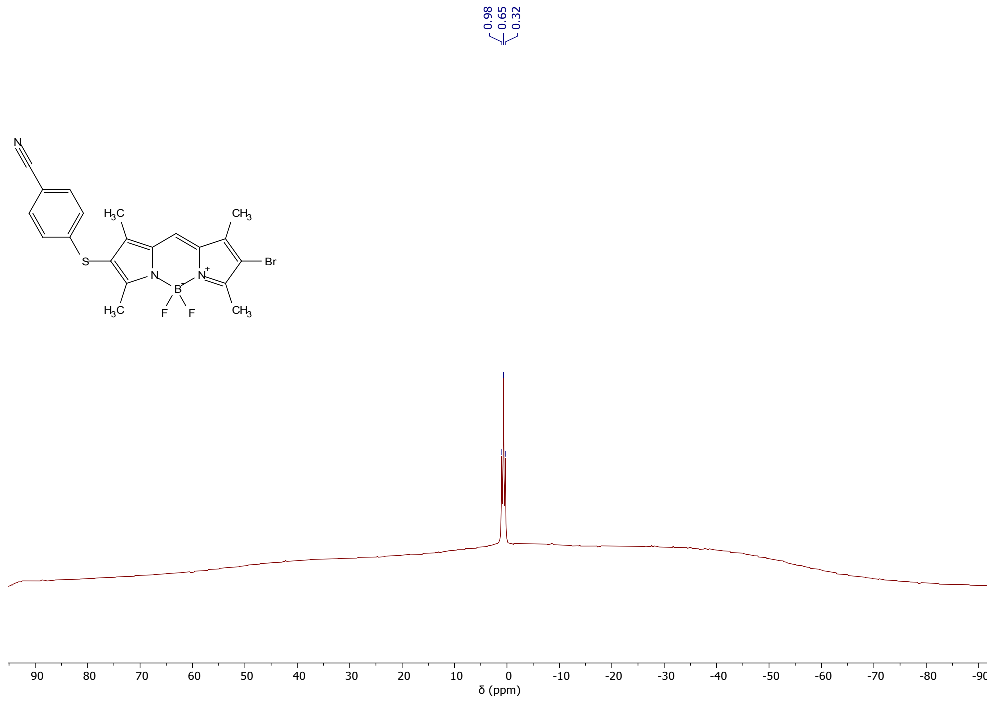

^{19}F NMR spectra of **2c**

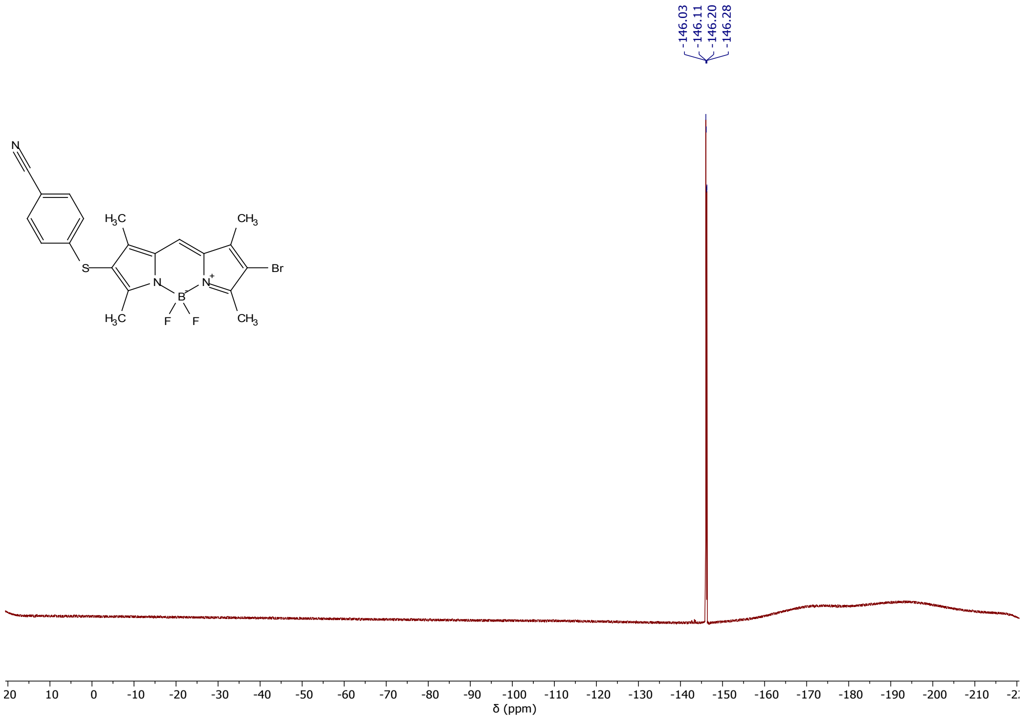

5- Computational details

Quantum chemical calculations have been performed using the ORCA 5.0.4[2] and Gaussian16[3] program packages. The ground state equilibrium geometries of all compounds have been optimized without symmetry constraints using DFT with the wB97XD,[4] CAM-B3LYP,[5] B3LYP,[6] B2PLYP[7] and PBE0 functionals[8] and the Ahlrichs def2-TZVP[9] basis set. To compute the absorption spectra, the lowest 10 singlet vertical excited states were calculated. TD-DFT calculations in ORCA were performed without the Tamm-Dancoff approximation (TDA).[10] The effect of the solvent was included using the linear-response conductor-like polarizable continuum model (LR-CPCM)[11] with hexane or DMSO as solvents. TD-DFT calculations were performed using the resolution-of-the-identity (RI) approximation under the RIJCOSX procedure.[12]

Table S1. Experimental and benchmark of computed absorption maxima (in nm) for BODIPY dye **2a** using the def2-TZVP basis set.

wB97XD	CAM-B3LYP	B3LYP	B2PLYP	PBE0	Experimental
440	437	446	466	447	528

Table S2. Experimental and computed absorption maxima (in nm) for BODIPY dyes **2a** and **2b** using the B2PLYP functional and the def2-TZVP basis set.

	Hexane	Hexane Exp.	DMSO	DMSO Exp.
**2a**	466	528	465	521
**2b**	480	531	476	524

Table S3. Computed energies in eV in hexane and DMSO for the lowest singlet and triplet states for **2a** and **2b** at the Franck-Condon geometries computed using the B2PLYP functional and the def2-TZVP basis set.

	S_1	S_2	T_1	T_2
**2a Hexane**	2.66	3.09	1.77	3.50
**2a DMSO**	2.67	3.30	1.78	3.50
**2b Hexane**	2.53	2.63	1.77	3.51
**2b DMSO**	2.60	2.70	1.78	3.50

2a S_0

C	-3.082443757265	1.288687088740	0.415043493169
C	-3.920569101245	0.144009446767	0.098467641590
C	-3.092705991469	-0.928919536913	-0.279166518900
C	-1.787618858093	0.831518072375	0.221456596822
C	1.992102429675	1.328227760906	0.239898529274
C	0.649540075895	0.856795977953	0.149132007685
C	1.955630057043	-0.872959981503	-0.415022947456
C	2.779948496919	0.232618376795	-0.120150727926
C	-0.548461917411	1.523184910696	0.372378127635
H	-0.548738507676	2.568961262885	0.674173560396
N	-1.817168133841	-0.502688430464	-0.181905822518
N	0.670964161293	-0.481466030250	-0.244173248833
B	-0.562984582103	-1.376564018424	-0.476828752689
F	-0.604916577008	-1.822580670729	-1.798316249318
F	-0.547132655143	-2.479407652779	0.378440656284
C	2.446171305924	2.689710088538	0.626758486021
H	3.022954904597	3.165849371747	-0.183672029215
H	1.599319047210	3.346450521473	0.867947977375
H	3.108533074229	2.655354185089	1.507695126257
C	2.339121262474	-2.239114295576	-0.838451291292
H	1.886900971586	-2.484276735542	-1.812660176146
H	3.430369103530	-2.324880667553	-0.914082778651
H	1.965157115744	-2.987981662423	-0.122091388465
C	-3.486605088216	2.676024114800	0.776212551044
H	-2.823708292024	3.412124459846	0.296928962023
H	-4.514585801219	2.897861625569	0.456630502447
H	-3.438261389771	2.854916111669	1.863833433284
C	-3.463049473766	-2.306484982003	-0.693474467258
H	-3.077427968875	-2.525672836362	-1.700890298968
H	-3.013083739748	-3.048353111800	-0.015637137077
H	-4.553224850826	-2.437954592115	-0.690186045283
Br	4.647674102420	0.227305558463	-0.200626697230
S	-5.619306624983	0.114286275974	-0.130273292623
C	-6.317086834820	0.874518139778	1.282327960666
C	-5.661650451614	0.927917761164	2.523190540569
C	-7.615434154709	1.396578012629	1.139096948232
C	-6.296775340515	1.524756720875	3.603598213669
H	-4.668205966352	0.488503175130	2.634433496642
C	-8.241478492321	1.986541427090	2.225907259199
H	-8.123027050350	1.343975254557	0.172884437965
C	-7.579460650471	2.061010829356	3.456037376723

H	-5.798383756844	1.561197313915	4.574194211566
H	-9.249354003497	2.392527134070	2.121125035405
C	-8.244658727331	2.762642723766	4.611561606336
F	-7.796664561679	2.318652956709	5.786063008644
F	-9.568679602631	2.595285416094	4.589594669577
F	-8.013076204721	4.079497129018	4.569247453349

2b S_0

C	-3.155800786842	1.102668388152	0.020017873098
C	-3.769271894910	-0.147453254050	-0.141540408961
C	-3.011394796160	-1.309525455375	-0.194144291768
C	-1.617541515700	-1.250819839513	-0.076471353886
C	-1.005676606422	-0.006620157282	0.089808817086
C	-1.762020531642	1.165152301033	0.133829766093
S	-0.551920317888	-2.678540720406	-0.155942962357
C	-1.660306837685	-4.027730394286	-0.014606465959
C	-2.215284973420	-4.531538550809	1.167482525447
C	-2.990749048228	-5.653243782077	0.774050417862
N	-2.889626133514	-5.813992234605	-0.602336007794
C	-2.097024399884	-4.850900665256	-1.090228414271
C	-2.049513410987	-3.983729198706	2.537907811808
C	-1.764083401128	-4.721138431440	-2.527966099962
C	-3.757572233954	-6.525535570751	1.537503681642
C	-4.446610093103	-7.586233847359	0.961790932096
C	-5.280745910369	-8.569726569916	1.561752745504
C	-5.702423974297	-9.368264589893	0.503535317233
C	-5.136994787023	-8.883223864111	-0.701882691461
N	-4.386168564013	-7.811529354712	-0.406140560011
C	-5.627555074136	-8.714981027843	2.999122247077
C	-5.297824565057	-9.412590473801	-2.076255714718
Br	-6.837776457209	-10.845471826122	0.643337414486
B	-3.573073845261	-6.949169320594	-1.425009952138
F	-4.432538048096	-6.406174851591	-2.370312743901
O	-3.975965499712	2.173676474573	0.056228238738
F	-2.606413388492	-7.728305184657	-2.043860860095
H	-3.818718601385	-6.372495867620	2.616506452777
H	-5.300331898287	-9.695042479194	3.381936575485
H	-5.164704989086	-7.934161931837	3.616748385236
H	-6.718676300655	-8.666125960850	3.143580305148
H	-4.317898883702	-9.685641060794	-2.496314312870
H	-5.952049634643	-10.292787914506	-2.076175902638
H	-5.722583030714	-8.640289772830	-2.735037224560
H	-2.089848158868	-4.768827368808	3.306243978520
H	-1.087868529239	-3.455848441216	2.616477425676
H	-2.840836318706	-3.249764105203	2.766094148350
H	-1.254834098869	-5.628663894614	-2.886482454887
H	-2.681615581512	-4.614633498322	-3.126066568992
H	-1.115626811848	-3.850196277014	-2.685034620159
H	-3.509583554802	-2.272550116426	-0.327283239444
H	0.081505792875	0.057839201534	0.189774044809
H	-4.857275697319	-0.184968604550	-0.230294029752
H	-1.249900397350	2.119217121818	0.263935243849
C	-3.414814511870	3.451162763489	0.202592091216
H	-4.249439900160	4.163690552919	0.200292914110
H	-2.863252755661	3.550730125139	1.153994726337
H	-2.733069043063	3.697461530285	-0.630346199098

2a Min S_1

C	-3.082443757265	1.288687088740	0.415043493169
C	-3.920569101245	0.144009446767	0.098467641590
C	-3.092705991469	-0.928919536913	-0.279166518900
C	-1.787618858093	0.831518072375	0.221456596822
C	1.992102429675	1.328227760906	0.239898529274
C	0.649540075895	0.856795977953	0.149132007685
C	1.955630057043	-0.872959981503	-0.415022947456
C	2.779948496919	0.232618376795	-0.120150727926
C	-0.548461917411	1.523184910696	0.372378127635
H	-0.548738507676	2.568961262885	0.674173560396
N	-1.817168133841	-0.502688430464	-0.181905822518
N	0.670964161293	-0.481466030250	-0.244173248833
B	-0.562984582103	-1.376564018424	-0.476828752689
F	-0.604916577008	-1.822580670729	-1.798316249318
F	-0.547132655143	-2.479407652779	0.378440656284
C	2.446171305924	2.689710088538	0.626758486021
H	3.022954904597	3.165849371747	-0.183672029215
H	1.599319047210	3.346450521473	0.867947977375
H	3.108533074229	2.655354185089	1.507695126257
C	2.339121262474	-2.239114295576	-0.838451291292
H	1.886900971586	-2.484276735542	-1.812660176146
H	3.430369103530	-2.324880667553	-0.914082778651
H	1.965157115744	-2.987981662423	-0.122091388465
C	-3.486605088216	2.676024114800	0.776212551044
H	-2.823708292024	3.412124459846	0.296928962023
H	-4.514585801219	2.897861625569	0.456630502447
H	-3.438261389771	2.854916111669	1.863833433284
C	-3.463049473766	-2.306484982003	-0.693474467258
H	-3.077427968875	-2.525672836362	-1.700890298968
H	-3.013083739748	-3.048353111800	-0.015637137077
H	-4.553224850826	-2.437954592115	-0.690186045283
Br	4.647674102420	0.227305558463	-0.200626697230
S	-5.619306624982	0.114286275974	-0.130273292623
C	-6.317086834820	0.874518139778	1.282327960666
C	-5.661650451614	0.927917761164	2.523190540569
C	-7.615434154709	1.396578012629	1.139096948232
C	-6.296775340515	1.524756720875	3.603598213669
H	-4.668205966352	0.488503175130	2.634433496642
C	-8.241478492321	1.986541427090	2.225907259199
H	-8.123027050350	1.343975254557	0.172884437965
C	-7.579460650471	2.061010829356	3.456037376723
H	-5.798383756844	1.561197313915	4.574194211566
H	-9.249354003497	2.392527134070	2.121125035405
C	-8.244658727331	2.762642723766	4.611561606336
F	-7.796664561679	2.318652956709	5.786063008644
F	-9.568679602631	2.595285416094	4.589594669577
F	-8.013076204721	4.079497129018	4.569247453349

2b Min S_1

C	-3.069681333130	1.168747434733	0.084521525629
C	-3.760634651122	-0.072202416745	0.067711274716
C	-3.068601627598	-1.252144951123	-0.015121026785
C	-1.651126810385	-1.234335236905	-0.087207299967
C	-0.962589648776	0.009423750935	-0.075372185354
C	-1.654983282965	1.194554849063	0.008743530219
S	-0.689987387846	-2.652016691234	-0.190059282301
C	-1.792184506446	-3.988374793763	-0.045674903297
C	-2.363826572904	-4.475188232728	1.171098698793
C	-3.076321769292	-5.618035357451	0.788660653230
N	-2.936297599142	-5.805955390741	-0.576413585739
C	-2.150886501236	-4.831845069240	-1.098783862331
C	-2.217201612815	-3.936651253717	2.553705842685
C	-1.810553506239	-4.750714789447	-2.545129611250
C	-3.843222265847	-6.516900811824	1.571165346509
C	-4.477262859466	-7.611974717751	0.971888457349
C	-5.278875027775	-8.632231734163	1.543398728999
C	-5.631785435200	-9.458581918185	0.464091599226
C	-5.069923949873	-8.962051619664	-0.716692053243
N	-4.370224578911	-7.839145234814	-0.392376999985
C	-5.656951566935	-8.794297416202	2.973331543038
C	-5.167665954952	-9.486790049885	-2.101466544555
Br	-6.699760203484	-10.997097177192	0.592062862736
B	-3.600505582904	-6.951043314978	-1.389078480818
F	-4.474282558475	-6.409263542455	-2.341703285334
O	-3.824833734963	2.243364988787	0.169817855119
F	-2.612735446645	-7.688206240209	-2.054320716124
H	-3.932524711970	-6.354196614385	2.643957894189
H	-5.267837092757	-9.737963245904	3.391681517521
H	-5.268273355905	-7.971069653200	3.588760264887
H	-6.752012439716	-8.821440189234	3.100772303304
H	-4.169113164390	-9.708163662818	-2.508832806116
H	-5.773495401433	-10.401939354395	-2.118814804748
H	-5.624457832469	-8.741883957790	-2.771793490240
H	-1.633275283910	-4.614290274356	3.199943679651
H	-1.704676921794	-2.963529931404	2.559788949919
H	-3.195643586250	-3.797663197144	3.042340033117
H	-1.338425449485	-5.684423591759	-2.884574031586
H	-2.717764354339	-4.617700551406	-3.154722004776
H	-1.127635372074	-3.913140853548	-2.742237008399
H	-3.586329921862	-2.212982285141	-0.031838745412
H	0.128704594228	0.017886647374	-0.130928779239
H	-4.850530768005	-0.053310796423	0.117368342768
H	-1.111963443100	2.139245815669	0.016914251642
C	-3.251449973403	3.540835385864	0.189286418560
H	-4.090785565757	4.240393907744	0.263224283741
H	-2.591783356617	3.663195087886	1.061562046648
H	-2.691120623665	3.734260251272	-0.737845396596

6- References

[1] Al Anshori, J.; Slanina, T.; Palao, E.; Klan, P. The internal heavy-atom effect on 3-phenylselanyl and 3-phenyltellanyl BODIPY derivatives studied by transient absorption spectroscopy. *Photochem. Photobiol. Sci.* **2016**, *15*, 250-259. DOI: 10.1039/c5pp00366k.

[2] Neese, F. Software update: The ORCA program system—Version 5.0. *WIREs Comput Mol Sci.* **2022**, *12*, e1606. DOI: 10.1002/wcms.1606.

[3] Gaussian 16, Revision C.01, Frisch, M. J.; Trucks, G. W.; Schlegel, H. B.; Scuseria, G. E.; Robb, M. A.; Cheeseman, J. R.; Scalmani, G.; Barone, V.; Petersson, G. A.; Nakatsuji, H.; Li, X.; Caricato, M.; Marenich, A. V.; Bloino, J.; Janesko, B. G.; Gomperts, R.; Mennucci, B.; Hratchian, H. P.; Ortiz, J. V.; Izmaylov, A. F.; Sonnenberg, J. L.; Williams-Young, D.; Ding, F.; Lipparini, F.; Egidi, F.; Goings, J.; Peng, B.; Petrone, A.; Henderson, T.; Ranasinghe, D.; Zakrzewski, V. G.; Gao, J.; Rega, N.; Zheng, G.; Liang, W.; Hada, M.; Ehara, M.; Toyota, K.; Fukuda, R.; Hasegawa, J.; Ishida, M.; Nakajima, T.; Honda, Y.; Kitao, O.; Nakai, H.; Vreven, T.; Throssell, K.; Montgomery, J. A., Jr.; Peralta, J. E.; Ogliaro, F.; Bearpark, M. J.; Heyd, J. J.; Brothers, E. N.; Kudin, K. N.; Staroverov, V. N.; Keith, T. A.; Kobayashi, R.; Normand, J.; Raghavachari, K.; Rendell, A. P.; Burant, J. C.; Iyengar, S. S.; Tomasi, J.; Cossi, M.; Millam, J. M.; Klene, M.; Adamo, C.; Cammi, R.; Ochterski, J. W.; Martin, R. L.; Morokuma, K.; Farkas, O.; Foresman, J. B.; Fox, D. J. Gaussian, Inc., Wallingford CT, **2016**.

[4] Chai, J.-D.; Head-Gordon, M. Long-range corrected hybrid density functionals with damped atom–atom dispersion corrections. ***Phys. Chem. Chem. Phys.*** **2008**, ***10***, 6615-6620. DOI: 10.1039/B810189B.

[5] Yanai, T.; Tew, D. P.; Handy, N. C. A new hybrid exchange–correlation functional using the Coulomb-attenuating method (CAM-B3LYP). *Chem. Phys. Lett.* **2004**, *393*, 51-57. DOI: 10.1016/j.cplett.2004.06.011.

[6] Decke, A. D. Density-functional thermochemistry. III. The role of exact exchange. *J. Chem. Phys.* **1993**, *98*, 5648-5652. DOI: 10.1063/1.464913.

[7] Grimme, S. Semiempirical hybrid density functional with perturbative second-order correlation. *J. Chem. Phys.* **2006**, *124*, 034108. DOI: 10.1063/1.2148954.

[8] Adamo, C.; Barone, V. Toward reliable density functional methods without adjustable parameters: The PBE0 model. *J. Chem. Phys.* 1999, *110*, 6158. DOI: 10.1063/1.478522.

[9] Weigend, F.; Ahlrichs, R. Balanced basis sets of split valence, triple zeta valence and quadruple zeta valence quality for H to Rn: Design and assessment of accuracy. *Phys. Chem. Chem. Phys.* **2005**, *7*, 3297-3305. DOI: 10.1039/B508541A.

[10] Hirata, S.; Head-Gordon, M. Time-dependent density functional theory within the Tamm–Dancoff approximation. *Chem. Phys. Lett.* **1999**, *314*, 291-299. DOI: 10.1016/S0009-2614(99)01149-5.

[11] Barone, V.; Cossi, M. Quantum calculation of molecular energies and energy gradients in solution by a conductor solvent model. *J. Phys. Chem. A* **1998**, *102*, 1995-2001. DOI: 10.1021/jp9716997.

[12] Izsák, R.; Neese, F. An overlap fitted chain of spheres exchange method. *J. Chem. Phys.* **2011**, *135*, 144105. DOI: 10.1063/1.3646921.
